# Building Programs to Eradicate Toxoplasmosis Part III: Epidemiology and Risk Factors

**DOI:** 10.1007/s40124-022-00265-0

**Published:** 2022-06-22

**Authors:** Mariangela Soberón Felín, Kanix Wang, Catalina Raggi, Aliya Moreira, Abhinav Pandey, Andrew Grose, Zuleima Caballero, Claudia Rengifo-Herrera, Margarita Ramirez, Davina Moossazadeh, Catherine Castro, José Luis Sanchez Montalvo, Karen Leahy, Ying Zhou, Fatima Alibana Clouser, Maryam Siddiqui, Nicole Leong, Perpetua Goodall, Morgan Michalowski, Mahmoud Ismail, Monica Christmas, Stephen Schrantz, Ximena Norero, Dora Estripeaut, David Ellis, Kevin Ashi, Samantha Dovgin, Ashtyn Dixon, Xuan Li, Ian Begeman, Sharon Heichman, Joseph Lykins, Delba Villalobos-Cerrud, Lorena Fabrega, Connie Mendivil, Mario R. Quijada, Silvia Fernández-Pirla, Valli de La Guardia, Digna Wong, Mayrene de LadrónGuevara, Carlos Flores, Jovanna Borace, Anabel García, Natividad Caballero, Maria Theresa Moreno de Saez, Michael Politis, Stephanie Ross, Mimansa Dogra, Vishan Dhamsania, Nicholas Graves, Marci Kirchberg, Kopal Mathur, Ashley Aue, Carlos M. Restrepo, Alejandro Llanes, German Guzman, Arturo Rebollon, Kenneth Boyer, Peter Heydemann, A. Gwendolyn Noble, Charles Swisher, Peter Rabiah, Shawn Withers, Teri Hull, Chunlei Su, Michael Blair, Paul Latkany, Ernest Mui, Daniel Vitor Vasconcelos-Santos, Alcibiades Villareal, Ambar Perez, Carlos Andrés Naranjo Galvis, Mónica Vargas Montes, Nestor Ivan Cardona Perez, Morgan Ramirez, Cy Chittenden, Edward Wang, Laura Lorena Garcia-López, Juliana Muñoz-Ortiz, Nicolás Rivera-Valdivia, María Cristina Bohorquez-Granados, Gabriela Castaño de-la-Torre, Guillermo Padrieu, Juan David Valencia Hernandez, Daniel Celis-Giraldo, John Alejandro Acosta Dávila, Elizabeth Torres, Manuela Mejia Oquendo, José Y. Arteaga-Rivera, Dan Nicolae, Andrey Rzhetsky, Nancy Roizen, Eileen Stillwaggon, Larry Sawers, Francois Peyron, Martine Wallon, Emanuelle Chapey, Pauline Levigne, Carmen Charter, Migdalia De Frias, Jose Montoya, Cindy Press, Raymund Ramirez, Despina Contopoulos-Ioannidis, Yvonne Maldonado, Oliver Liesenfeld, Carlos Gomez, Kelsey Wheeler, Ellen Holfels, David Frim, David McLone, Richard Penn, William Cohen, Samantha Zehar, James McAuley, Denis Limonne, Sandrine Houze, Sylvie Abraham, Raphael Piarroux, Vera Tesic, Kathleen Beavis, Ana Abeleda, Mari Sautter, Bouchra El Mansouri, Adlaoui El Bachir, Fatima Amarir, Kamal El Bissati, Alejandra de-la-Torre, Gabrielle Britton, Jorge Motta, Eduardo Ortega-Barria, Isabel Luz Romero, Paul Meier, Michael Grigg, Jorge Gómez-Marín, Jagannatha Rao Kosagisharaf, Xavier Sáez Llorens, Osvaldo Reyes, Rima McLeod

**Affiliations:** 1 Toxoplasmosis Programs and Initiatives in Panamá, Ciudad de Panamá, Panama; 2 Institute for Genomics and Systems Biology, The University of Chicago, Chicago, IL, USA; 3 Pritzker School of Medicine, The University of Chicago, Chicago, IL, USA; 4 Instituto de Investigaciones Científicas Y Servicios de Alta Tecnología AIP (INDICASAT-AIP), Ciudad de Panamá, Panama; 5 Department of Pediatrics Infectious Diseases/Department of Neonatology, Hospital del Niño Doctor José Renán Esquivel, Ciudad de Panamá, Panama; 6 Department of Ophthalmology and Visual Sciences, The University of Chicago, Chicago, IL, USA; 7 The College, The University of Chicago, Chicago, IL, USA; 8 The Global Health Center, The University of Chicago, Chicago, IL, USA; 9 Universidad de Panamá, Ciudad de Panamá, Panama; 10 Department of Statistics, The University of Chicago, Chicago, IL, USA; 11 Rush University Medical School/Rush University Medical Center, Chicago, IL, USA; 12 Academia Interamericana de Panamá, Ciudad de Panamá, Panama; 13 Hospital Santo Tomás, Ciudad de Panamá, Panama; 14 Hospital San Miguel Arcángel, Ciudad de Panamá, Panama; 15 Capstone Program, Global Health Center, The University of Chicago, Chicago, IL, USA; 16 Harris School of Public Policy, The University of Chicago, Chicago, IL, USA; 17 Sanofi Aventis de Panamá S.A, University of South Florida, Ciudad de Panamá, Panama; 18 Northwestern University Feinberg School of Medicine, Chicago, IL, USA; 19 NorthShore Evanston Hospital, Evanston, IL, USA; 20 Department of Microbiology, The University of Tennessee, Knoxville, TN, USA; 21 Universidad de Federal de Minas Gerais, Minas Gerais, Brazil; 22 Universidad Autónoma de Manizales, Manizales, Colombia; 23 Universidad del Quindío, Armenia, Colombia; 24 Grupo de Investigación en Neurociencias, Universidad del Rosario, Bogotá, Colombia; 25 The University of South Florida College of Public Health, Tampa, FL, USA; 26 Department of Economics, Gettysburg College, Gettysburg, PA, USA; 27 Department of Economics, American University, Washington, D.C, USA; 28 Institut Des Agents Infectieux, Hôpital de La Croix-Rousse, Lyon, France; 29 Remington Specialty Laboratory, Palo Alto, CA, USA; 30 Department of Pediatrics, Division of Infectious Diseases, Stanford University College of Medicine, Stanford, CA, USA; 31 Roche Molecular Diagnostics, Pleasanton, CA, USA; 32 LDBioDiagnostics, Lyon, France; 33 Laboratory of Parasitologie, Bichat-Claude Bernard Hopital, Paris, France; 34 INH, Rabat, Morocco; 35 Faculty of Sciences Aïn Chok, University Hassan II, Casablanca, Morocco; 36 Sistema Nacional de Investigadores de Panamá (SNI), Clayton, Panama; 37 Tecnología E Innovación (SENACYT), Secretaría Nacional de Ciencia, Ciudad de Panamá, Panama; 38 GSK Vaccines, Panamá, Panama; 39 Molecular Parasitology Section Laboratory of Parasitic Diseases, National Institutes of Health, NIAID, Bethesda, MD, USA; 40 Toxoplasmosis Center, The University of Chicago, and Toxoplasmosis Research Institute, Chicago, IL, USA; 41 Department of Pediatrics (Infectious Diseases), The University of Chicago, Chicago, IL, USA

**Keywords:** *Toxoplasma*, Toxoplasmosis, Prevalence, Incidence, Screening, Risk factors, Spatial epidemiology

## Abstract

**Purpose of Review:**

Review comprehensive data on rates of toxoplasmosis in Panama and Colombia.

**Recent Findings:**

Samples and data sets from Panama and Colombia, that facilitated estimates regarding seroprevalence of antibodies to *Toxoplasma* and risk factors, were reviewed.

**Summary:**

Screening maps, seroprevalence maps, and risk factor mathematical models were devised based on these data. Studies in Ciudad de Panamá estimated seroprevalence at between 22 and 44%. Consistent relationships were found between higher prevalence rates and factors such as poverty and proximity to water sources. Prenatal screening rates for anti-*Toxoplasma* antibodies were variable, despite existence of a screening law. Heat maps showed a correlation between proximity to bodies of water and overall *Toxoplasma* seroprevalence. Spatial epidemiological maps and mathematical models identify specific regions that could most benefit from comprehensive, preventive healthcare campaigns related to congenital toxoplasmosis and *Toxoplasma* infection.

## Introduction

The devastating effects of toxoplasmosis—especially the congenital form—are well documented, with a 2013 World Health Organization (WHO) report estimating that congenital toxoplasmosis (CT) creates 1.20 million disability-adjusted life years (DALYs) worldwide [1••, 2, 3]. However, in our primary country of focus—Panama—data on *Toxoplasma* is sparse, despite several indications of high prevalence. For example, a 1988 study estimated that Panama has one of the highest rates of *Toxoplasma* infection in Latin America, with seroprevalence of 50% in 10 year olds and 90% in 60 year olds [4•]. Meanwhile, the neighboring countries of Colombia and Costa Rica have estimated seroprevalences of 43–67% and 49–61%, respectively [5, 6, 7, 8, 9]. With respect to CT in Panama, annual incidence as estimated by the WHO is 1.8 cases/1000 live births/ year, associated with 840 DALYs [3].

A 2014 hospital-based study of *Toxoplasma* seroprevalence by Montenegro Vasquez et al. (see Part II) helped us define the scope of the problem in Panama, but we sought to better understand regional discrepancies in disease burden, compliance of screening for *Toxoplasma* among pregnant women (following passage of a mandatory gestational screening law; see part II), and particular risk factors associated with *Toxoplasma* acquisition.

As such, one priority of our wide-ranging public health project was generating more comprehensive data on rates of toxoplasmosis in Panama (and later, Colombia). We also sought to create spatial epidemiological maps and mathematical models that could help us identify risk factors associated with the disease as it takes place in Central and South America.

## Approach

### Overview and Chronology

As in the educational initiatives, our understanding of spatial epidemiology and risk factors grew from a foundation of student projects in a growing global health program during that time which has become the Kiphart School of Public Health at The University of Chicago. This involved students from other universities as well. Each student began with a hypothesis, a null hypothesis, and a systematic approach to answering a question. These summer programs provided a foundation for the spatial epidemiology understanding in this work. These studies took place over consecutive summers, each involving at least two undergraduate students, often a medical student and a mentor. This was done in collaboration with an in-country team and grew within each country subsequently. The evolution of this work is shown chronologically in the diagram in the Box.

Using samples from screening programs for *Toxoplasma* in Ciudad de Panamá, Panama, and Armenia, Colombia, estimates and trends regarding seroprevalence of antibodies to the parasite were compiled. Screening maps, seroprevalence maps, and risk factor mathematical models were devised based on these data. Most of the materials come from independent investigations conducted by students who were affiliated with global health research programs at the University of Chicago. As more student contributors were graciously invited to work with individuals and institutions in Panama and Colombia, the studies that these students completed and presented became part of a truly international public health initiative, one that quickly involved more institutions and collaborators than many of us had originally conceived. None of these projects would have been possible without the collaboration of numerous US and in-country partners. As such, each contributor’s principal partners are highlighted in supplementary materials that were presented by each of the Chicago students at the end of the summer.

For each student this included abstracts, presentations, papers, and posters at Global Health student programs. These contributions are included in the supplemental materials as the students themselves prepared and presented their work with in-country partners. From each country, parallel manuscripts were prepared utilizing the same data sets. The approach utilized in these studies also has subsequently extended to other countries as well. The approach for each summer is presented first. Then the updates of what the students learned and developed are presented next and discussed.

### Screening Rates and Prevalence of T. gondii in Panama (2016–2017)

In 2016, a chart review was conducted on a convenience sample of pregnant women at Hospital Santo Tomas (HST) and Hospital San Miguel Arcángel (HSMA) in Ciudad de Panamá. Information gathered included *Toxoplasma* screening data, health center/private clinic names, and demographics (age, race, education, literacy). Patient prenatal control cards were requested from outpatients at HST; data were collected anonymously from HST inpatients and all HSMA patients.

**Fig. 1:**
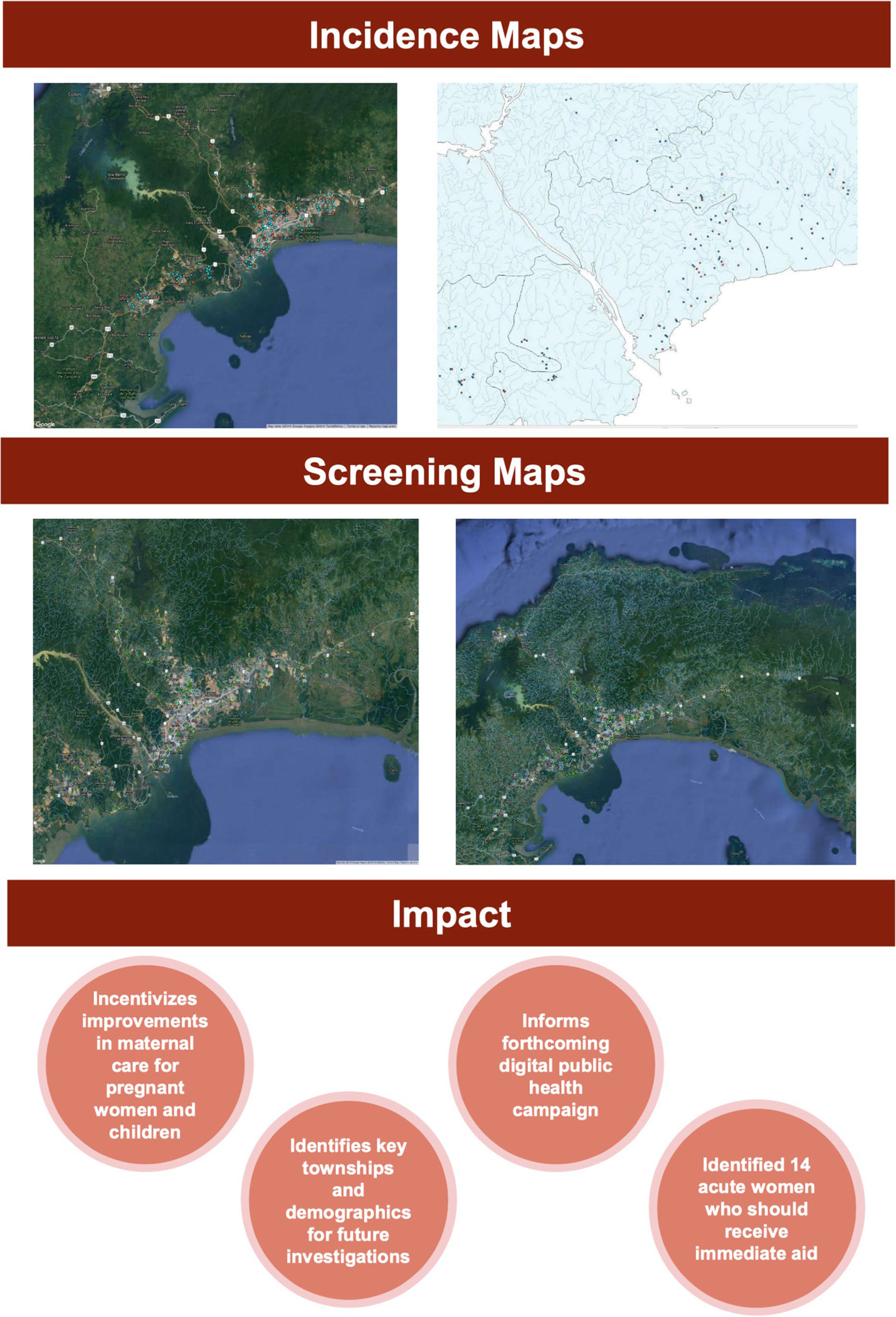
Sample slides from presentation by Pandey, Moreira, Wang, Rzhetsky, McLeod et al. that details their studies in Panama. One component of their research was a study on the effectiveness of using digital media to teach pregnant women about congenital toxoplasmosis. Pandey and Moreira also created incidence and screening maps for toxoplasmosis in Panama; these maps were based on screening data, IgG/IgM test results, demographic data, and addresses from prenatal control charts. See Supplement for complete presentation

Patient addresses were used to map screening rates and *T. gondii* prevalence in Ciudad de Panamá. Health center information was used to determine which health centers and private clinics provided mandatory screening. Data from patients who had been screened and tested positive for IgM and/or IgG were used to map *Toxoplasma* prevalence (Fig. 1; Supplement: Wang et al.; Moreira and Pandey et al.)

In 2017, a follow-up study was conducted with a convenience sample of pregnant women in HSTs maternity wing. Participants were administered questionnaires asking about additional demographic information (address, hometown size, education, age) and history of exposure to toxoplasmosis risk factors (contact with animals, water source, food hygiene). Five-milliliter blood samples were collected from participants and screened for detection of anti-*Toxoplasma* IgG/IgM. Data were analyzed using Pearson’s chi-squared and Fisher’s exact tests in R. Patient addresses were used to create maps of acute infection and relative screening rates (Supplement: Moossazadeh et al.; Ramirez et al.).

### Risk Prediction Model for T. gondii Infection in Panama (2017)

In 2017, prospective assessments of 341 pregnant women at HST’s hospital maternity ward were used to develop a predictive model for risk of exposure to *Toxoplasma gondii*. Sera from selected patients were tested for anti-*Toxoplasma* IgG; patients who had been tested for antibodies in the 2 weeks prior to their visit were excluded.

Additionally, seronegative and seropositive patients were screened for factors that included demographic information, contact with wild animals, food hygiene, and food and water sources, all referred to in a 2004 study of toxoplasmosis risk factors by Etheredge et al. [10•]. Past laboratory test results were obtained from patient charts. All statistical tests and analyses were performed in RStudio (Version 3.1.3, Vienna, 2015). See Supplement: Moossazadeh et al.

#### Spatial Analysis of Toxoplasmosis Screening and Seroprevalence in Panama (Supplement by Raggi et al. 2019)

In 2019, seroprevalence and screening rates in Panama were again mapped using data from previous studies by the University of Chicago and Panama’s Institute of Scientific Research and High Technology Services (INDICASAT). The analysis used both qualitative and quantitative spatial methods, as well as log odds regression. KDE heatmaps used available point data. When possible, data points were condensed into regional data for polygon-based analyses such as natural break and standard deviation heatmaps. Moran’s I cluster analysis was used to search for spatial clustering or outliers.

Locations and outlines of Panama’s hydraulic systems and water basins were found as digitized Tommy Guardia maps by the Smithsonian Tropical Research Institute. Similarly, water treatment plants run by Panama’s Institute of Aqueducts and Sewage Systems (IDAAN) were copied from the IDAAN website. Seroprevalence and screening rates of each township in Ciudad de Panamá were then used to make standard deviation, natural break, and Moran’s I cluster maps in Geoda that were overlaid with water system information in QGis (Supplement: Raggi et al.).

### Screening Rates, Prevalence, and Risk Factors Related to T. gondii in Colombia (2019)

In 2019, screening rates, seroprevalence, and risk factors for *T. gondii* infection were mapped using data from Quindío, Colombia. Physicians from the University of Quindío set up temporary clinics providing free eye exams and testing for *Toxoplasma* infection in the townships of Armenia, La Universal, and Guaduales de la Villa. While waiting to be attended, participants were interviewed about their behavior regarding suspected toxoplasmosis risk factors. Interview questions were used as indicator variables and added to a spreadsheet that included participants’ addresses and exam results. Data were analyzed in RStudio using a log odds linear regression to identify risk factors in each township and in Armenia as a whole. Mapping of *T. gondii* prevalence and screening involved KDE heatmaps, polygon-based analyses, and Moran’s I cluster analysis (Supplement: Raggi et al.).

## Update

### Screening Rates and Prevalence of T. gondii in Panama (2016–2017)

Among 665 pregnant women, Moreira and Pandey et al. found that 38.9% had received screening and, of those screened, 21.6% were seropositive for IgG and 5.5% for IgM. IgG seropositivity was found in all age and education groups; indigenous women were more likely to test positive for toxoplasmosis. Screening rates for *T. gondii* infection in pregnant women at public health centers and private clinics varied from 0 to 100%. Overall, however, private clinics had a much higher screening rate than public health centers.

**Fig. 2:**
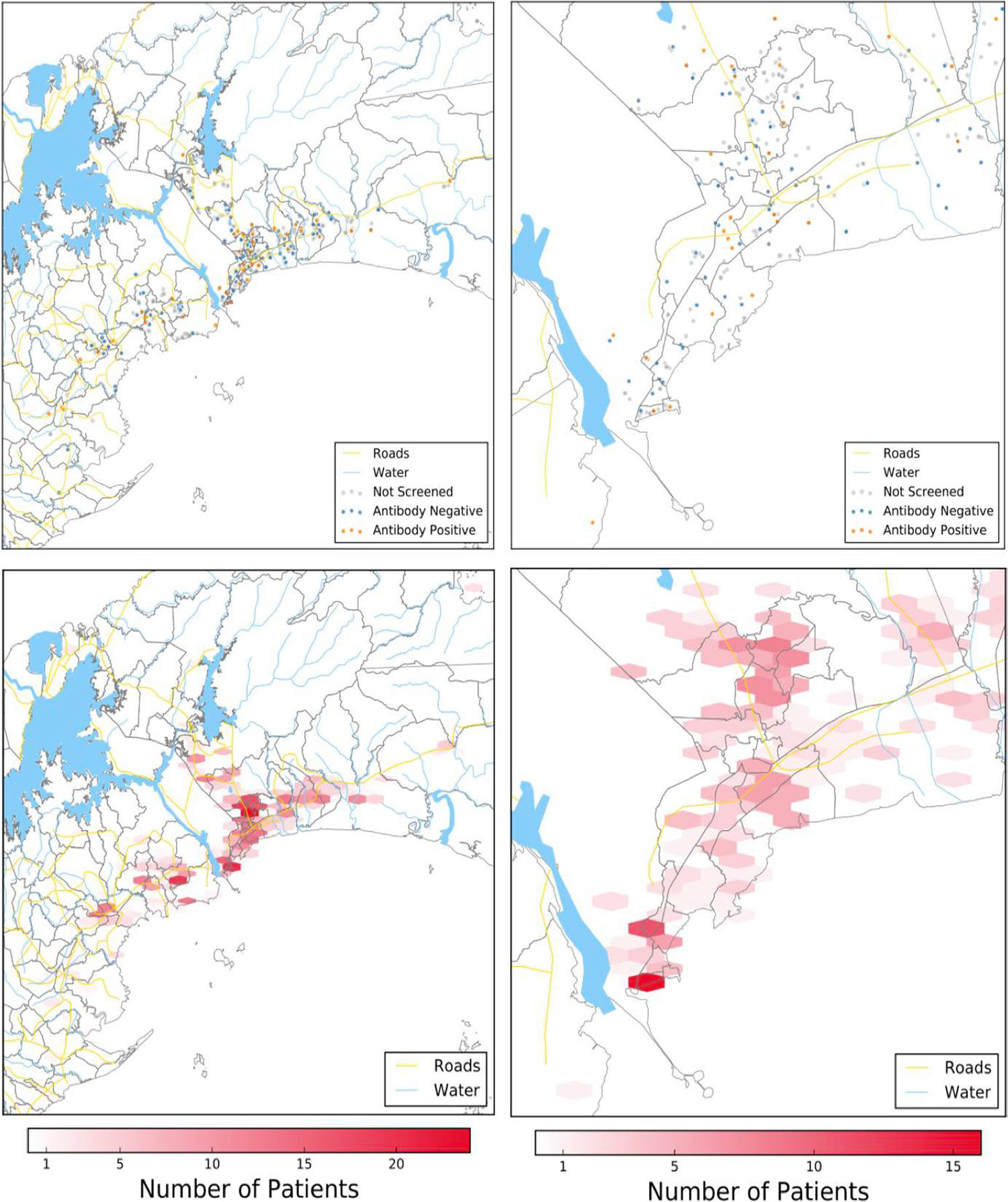
Dot and scatter hexbin maps based on Moreira and Pandey et al.’s data on relative screening frequencies of patients in the Ciudad de Panamá metropolitan region. In the top two maps, gray points represent patients not screened for toxoplasmosis, orange points represent patients positive for infection, and blue points represent seronegative patients. Across all four maps, major trends in screening and case frequency include higher frequency of screening from north (more rural) to south (more urban) and higher seropositivity rates along roads and waterways

**Fig. 3:**
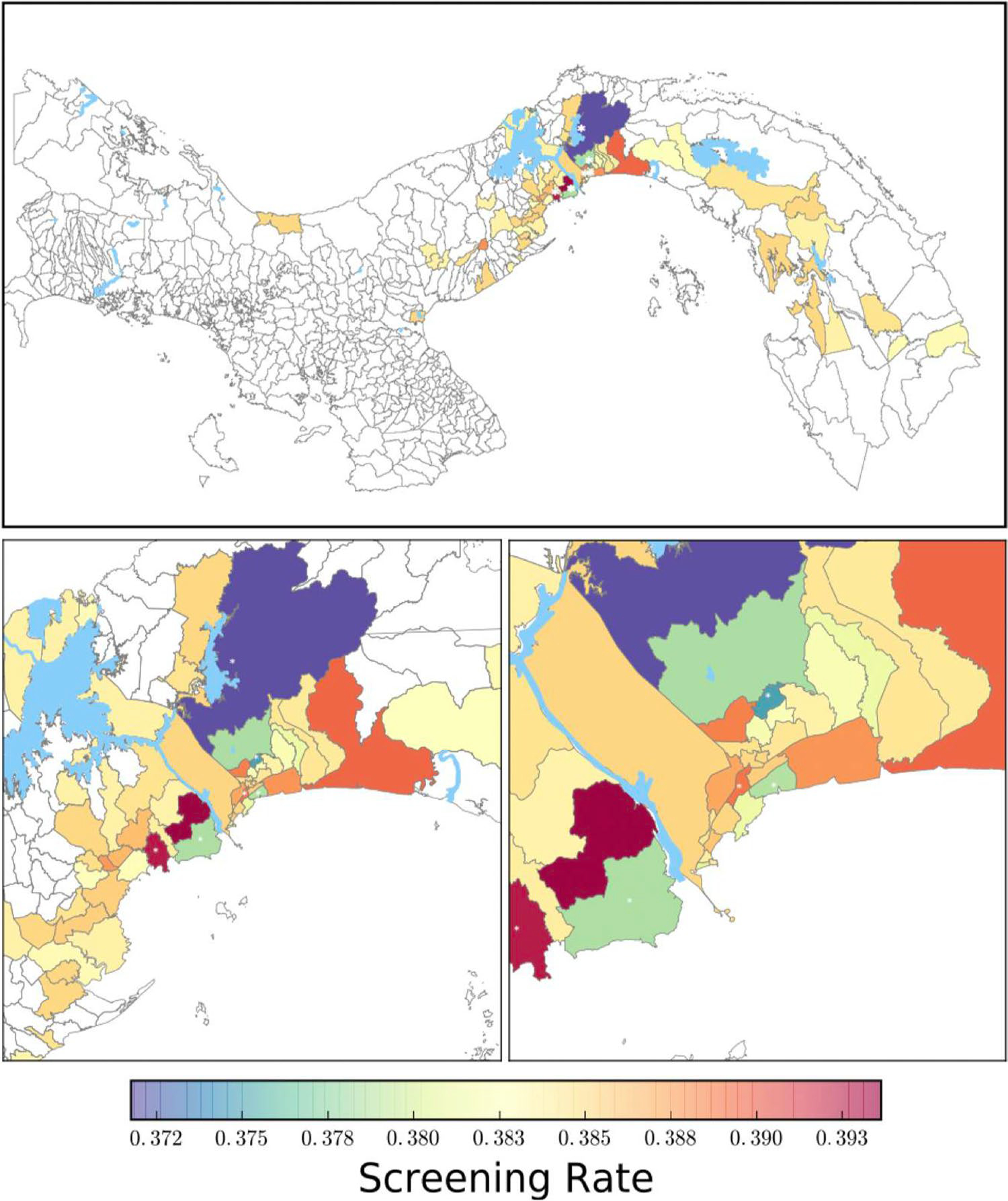
Maps of screening rates for CT by *corregimiento* (township) at three different scales, based on Wang et al.’s findings and Moreira and Pandey et al.’s data. Screening rates were calculated using a Bayesian prior of Beta(254, 411). Average screening rate among the townships represented by the women with available data was 38.2%. Townships with much lower or higher than average screening rates are marked with white asterisks

**Fig. 4:**
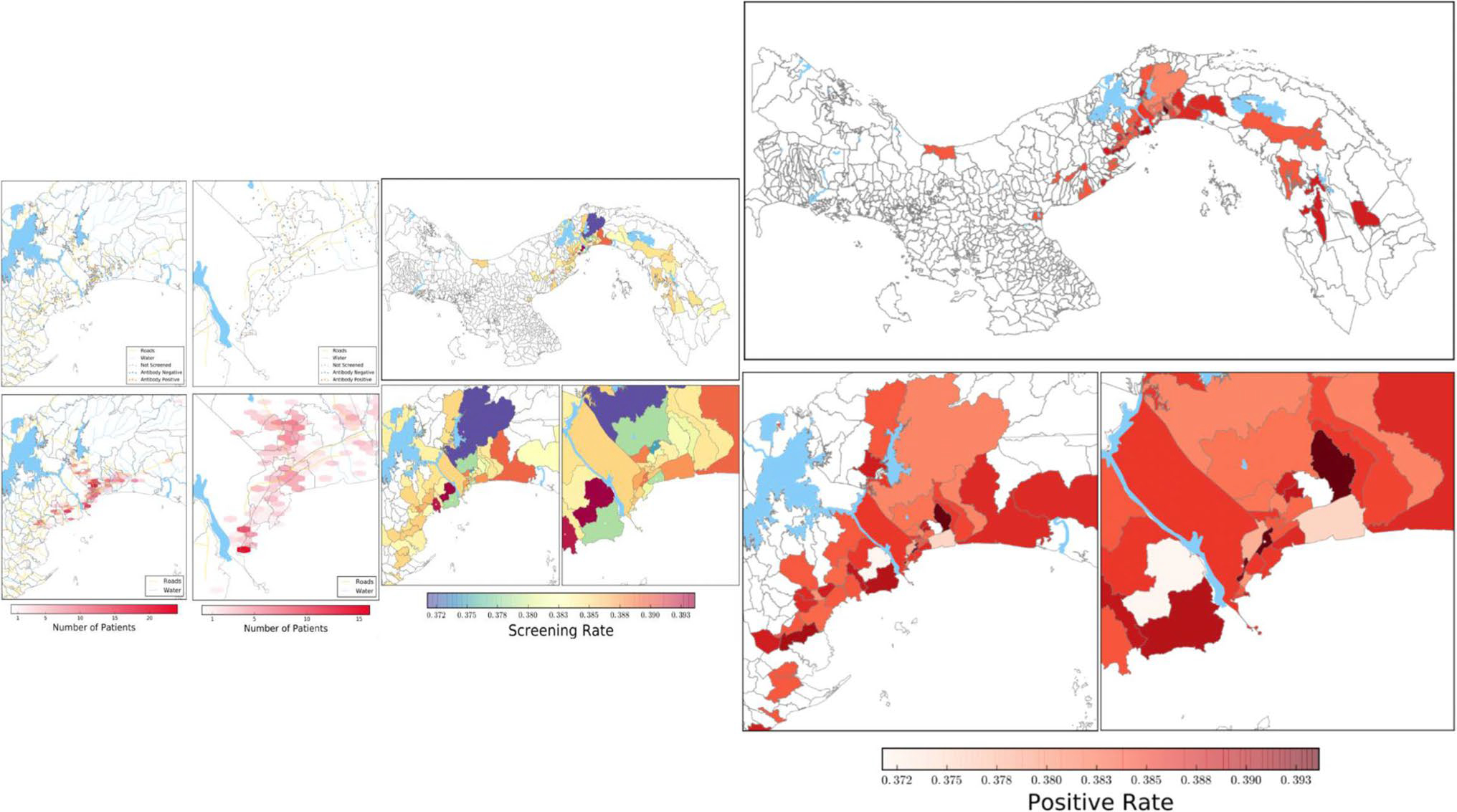
Summary of maps from Figs. 2 and 3, along with a map of toxoplasma seroprevalence by *corregimiento* (township) at three different scales. Prevalence rates were calculated using a Bayesian prior of Beta(63, 191). Average prevalence of toxoplasmosis among the townships represented by the women with available data was 24.8%. The highest rates of toxoplasmosis were observed in three provinces, shown in very dark red in the highest-resolution map, from west to east: Curundú, Pueblo Nuevo, Pedregal

Logistic regression analysis showed statistically significant longitudinal trends in positive antibody test results, with residents farther to the east of Panama having higher infection rates. Seropositivity rates trended upward around roads and waterways, with three positive cases near the Curundú River. With respect to screening rates, residents to the South had significantly higher chances of being screened than their northern, rural counterparts (Figs. 2, 3, and 4; Supplement: Wang et al.).

**Fig. 5:**
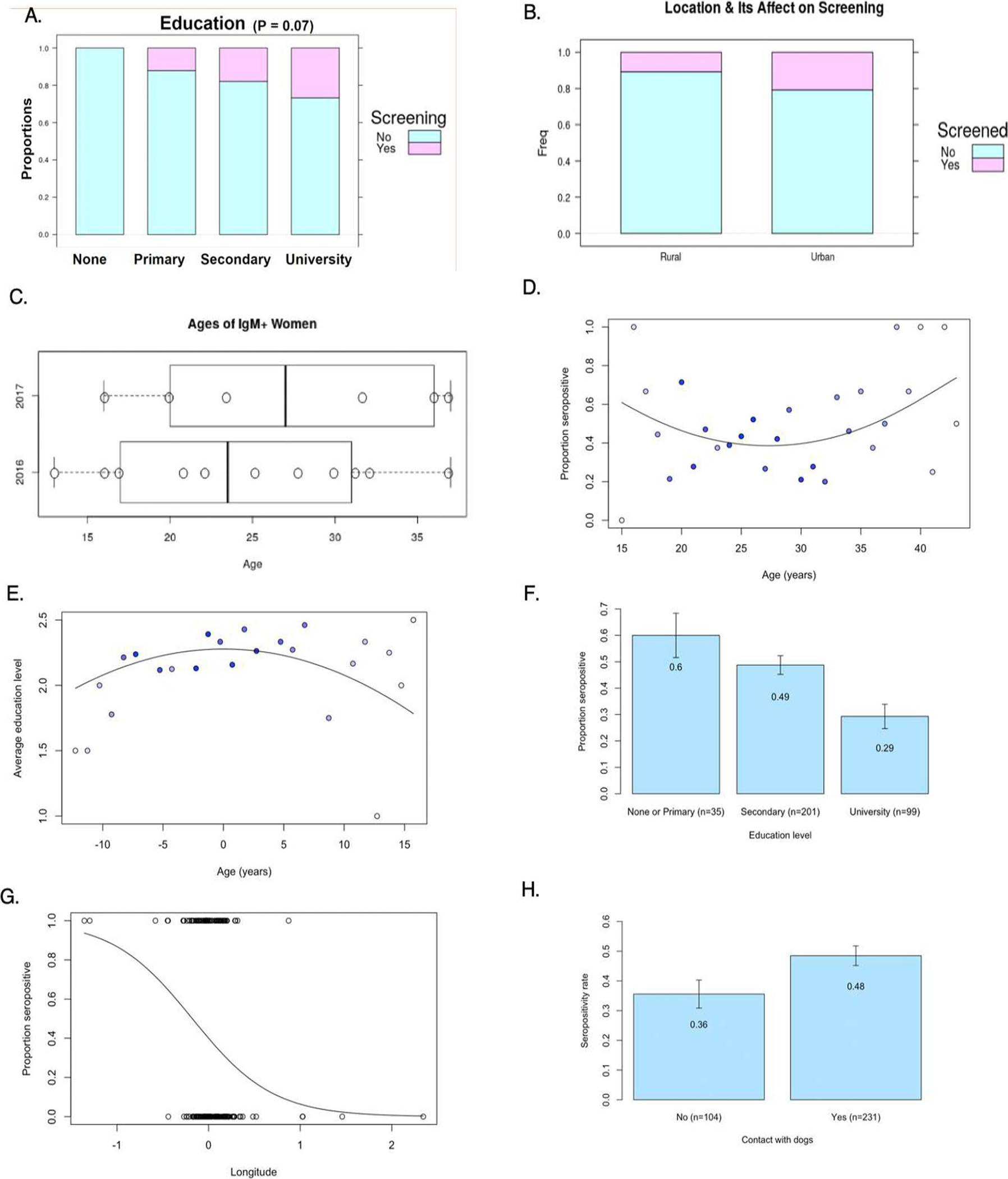
Summary of findings from Moossazadeh’s study of risk factors and development of a mathematical model for predicting highest-risk areas of *Toxoplasma* infection in Panama **A** A strong, but not significant, inverse relationship between highest level attained and screening compliance was found. **B** Rural-urban comparison showed that pregnant women in urban areas were more likely to be screened; the relationship was not significant. **C** Ages of IgM + women show that average age of educational; IgM positive women in both 2016–2017 studies was lower than the mean age of their cohorts (25.57 ± 9.03 cf. 27.28 ± 6.23 and 24.43 ± 7.24 cf. 26.59 ± 7.15, respectively). **D** *T. gondii* IgG seropositivity rates among pregnant women by maternal age, plotted with age centered around the mean. The curve represents the fit from the logistic regression of IgG status against age squared, with age centered around its mean. The points are shaded according to the number of women at each age, where darker colors signify a greater number of women. **E** Average education level among pregnant women by maternal age, plotted with age centered around the mean. **F** *T. gondii* IgG seropositivity rates among pregnant women by education level. Error bars represent the standard error of the mean. **G** *T. gondii* IgG seropositivity rates among pregnant women by longitude. Zero degree represents the longitude of Hospital Santo Tomás. **H** IgG seropositivity rate based on contact or no contact with dogs (both in the street or as pets). Error bars represent the standard error of the mean

Moossazadeh et al. and Ramirez et al. found that—among 343 pregnant women at HST—19.8% had received screening, and, of those screened, 44% were seropositive for IgG and 2.0% for IgM. Location (longitude, east, Curundu), race (Caucasian), and age (older) were all significantly associated with screening. Chi-square analysis on screening rates and seropositivity in pregnant women in Panama revealed a trend between higher education and screening rates (*p* = 0.07). However, seropositive women spanned the education level spectrum (Fig. 5A). Figure 5B indicates that pregnant women in urban areas were more likely to be screened, although the difference was not significant.

In both of the studies above, average age of acutely infected women was lower than the average of their respective cohorts (25.57 ± 9.03 cf. 27.28 ± 6.23 and 24.43 ± 7.24 cf. 26.59 ± 7.15, respectively). Figure 5C compares the age distribution of acutely infected women from our 2016 and 2017 studies.

### Risk Prediction Model for T. gondii Infection in Panama (2017)

A plot of the proportion of anti-*Toxoplasma* IgG-positive patients against age exhibits a quadratic curve (Fig. 5D). Education levels were highest at the extreme ends of the age spectrum, while patients around the mean age had a lower mean education level (Fig. 5E). Univariate analyses found (age squared) insignificant when controlling for effects of education (age squared becomes *p* = 0.07 from *p* = 0.018). A *χ*2 analysis of IgG status against education level yielded a significant negative correlation (*p* = 0.0008) between the two (Fig. 5F). This yielded the following formula, where the coefficient on education is significant (*p* = 0.0003).

logit(p)=1.3−0.7e

A χ2 analysis of IgG status versus home location (urban or rural) did not yield significant results (*p* = 0.82). Distance from the hospital, distance from water, and latitude were also not significant in predicting IgG seropositivity (p = 0.98, 0.22, and 0.62, respectively). Longitude was significant (*p* = 0.004), with seroprevalence decreasing from east to west (Fig. 5G). A logistic regression of IgG status on longitude yields the following formula:

logit(p)=−0.41−2.29( long+79.5).

Contact with dogs was the only animal-related factor that showed a positive significant correlation (*p* = 0.037) with seropositivity (Fig. 5H).

When variables related to diet were analyzed, a logistic regression of pig on seafood yielded an odds ratio of 9.7, and ordinal regressions of raw meat on both pig and seafood yielded odds ratios of 2.2 and 2.4, respectively. A log-linear model of the three-way interaction between pig, seafood, and raw meat was significant (*p* = 0.012).

While a significant positive correlation was found between hand washing and produce washing (*p* = 5.7 × 10 − 7), neither variable was significantly associated with IgG seropositivity (*p* = 0.41 and 0.57, respectively).

Most patients obtained their water from an aqueduct (plumbing), while a few obtained their water from wells, rivers, and rainwater, grouped together as “other.” Water source was not significantly associated with IgG seropositivity (Fisher’s exact test, *p* = 0.56).

Overall, single logistic regressions found only two variables, education (χ2 test, *p* = 0.0008) and longitude (logistic regression, *p* = 0.004), had significant and independent correlation with IgG seropositivity. A multiple logistic regression of IgG status against these two variables was run on the 200 patients for whom there was no missing data. Of these 200 patients, 119 were seronegative and 81 were seropositive. Logistic regression yielded the following equation, where both variable coefficients were significant (*p* = 0.001 and 0.005, respectively):

logit(p)=202.9−0.9e+2.5long.

This equation was used to generate the predicted probability of seropositivity (*p*) for each of the 200 patients, rounded to the nearest integer (0 or 1) and compared to actual seropositivity status. The model yielded a root mean square error (RMSE) of 0.46, a margin of error (ME) of 0.43, and accuracy of 0.68, as well as a false positive rate of 0.11 and a false negative rate of 0.64.

### Spatial Analysis of Toxoplasmosis Screening and Seroprevalence in Panama (2019)

**Fig. 6:**
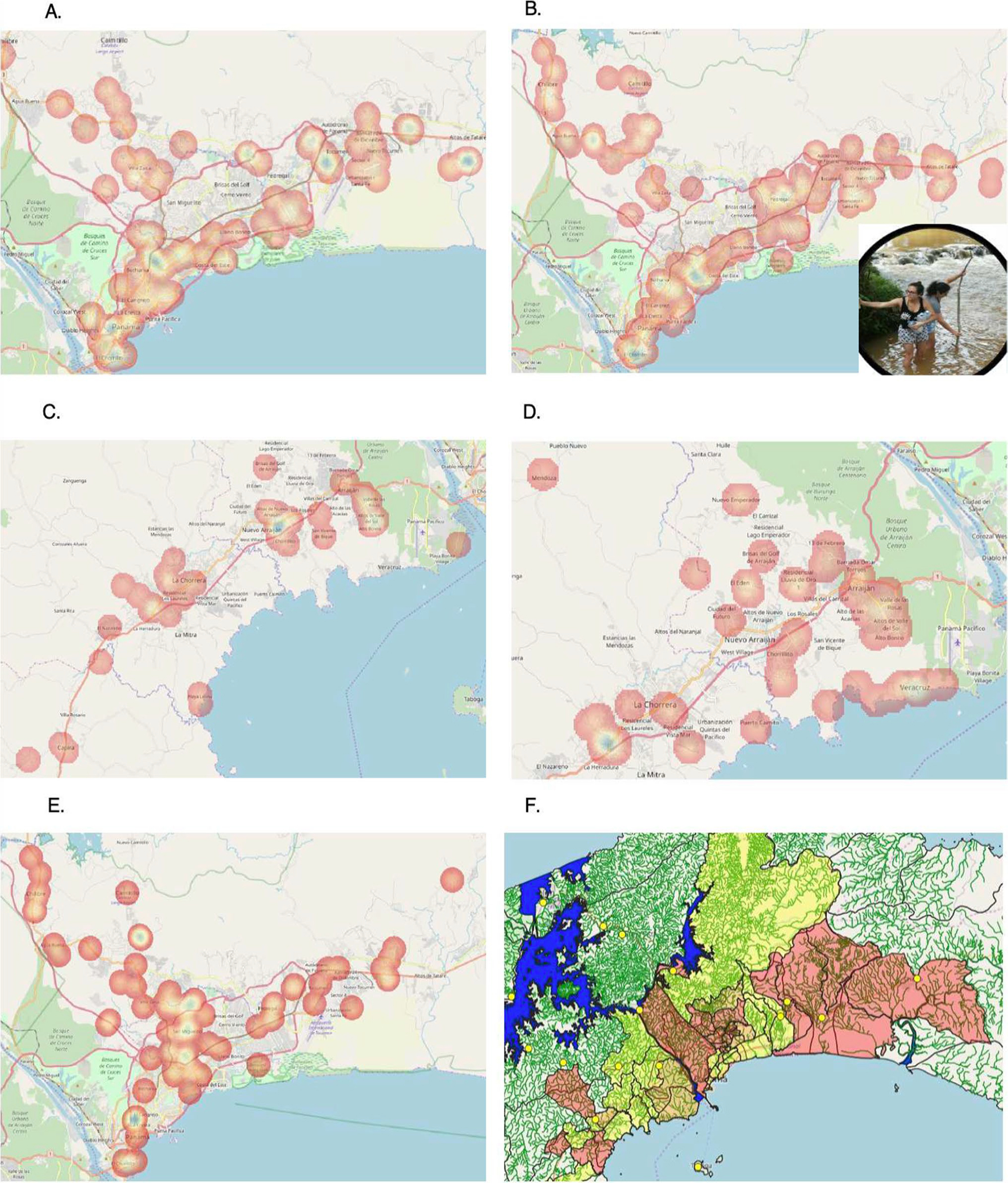
Summary of Raggi’s spatial epidemiological study of toxoplasma screening and seroprevalence rates in Panama. **A** KDE of previously screened individuals in the province of Panamá (*n* = 88). **B** KDE of previously unscreened individuals in the province of Panamá (*n* = 190). **C** KDE of previously screened individuals in Panamá Oeste (*n* = 60). **D** KDE of previously unscreened individuals in Panamá Oeste (*n* = 70). **E** KDE of positive IgG points in the province of Panamá (*n* = 83). **F** Aggregated toxoplasmosis prevalence rates based on Panama’s *corregimientos*, overlaid on Panama’s water systems

Figure 6A shows the distribution and concentration of pregnant women who had been previously screened for toxoplasmosis, and Fig. 6B shows those who had not been screened. The two maps showed distinct distributions, with Fig. 6B having a larger amount of points to the east and the north. The KDE for unscreened individuals also showed clusters around the Pedregal township, with roughly 11 points.

Figures 6C and D show the distribution of pregnant women who had been screened and not screened, respectively, in the province of Panamá Oeste. A cluster of screened individuals was found in the town of Nuevo Arraiján. In addition, almost every point in the township of Veracruz region, and on much of the coast in general, was marked as being from an unscreened individual. The distribution of screened individuals also closely followed the location of the Arraiján-Chorrera Freeway (the red line displayed on the map), while the unscreened distribution was more scattered (Fig. 6E).

The natural break map focusing on water sources in Panamá identified clusters of seropositive patients around water sources, as can be seen by the two large red sections both immediately to the right of the Panama Canal and at the far right of the map (Fig. 6F). Seroprevalence also trended upward from west to east.

### Screening Rates, Prevalence, and Risk Factors Related to T. gondii in Colombia (2019)

**Fig. 7:**
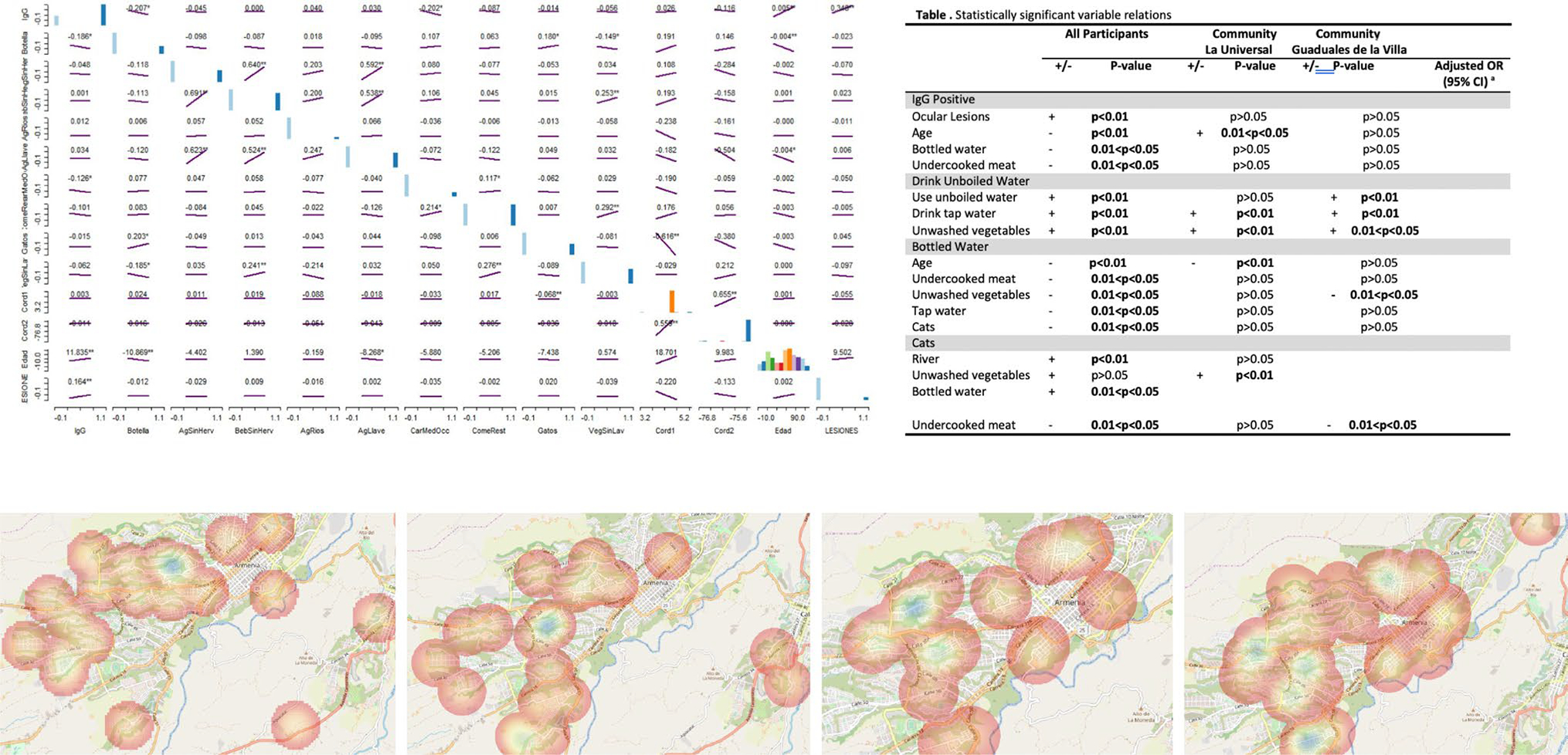
Summary of mapping studies from Colombia. **A** Scatterplot matrix of all variables used in the Armenia study. **B** Statistically significant variable relationships and their *p* value ranges for the entire population surveyed. **C** Heat map showing the distribution of all cases of CT that went untreated in Quindío (*n* = 23). **D** Heat map showing cases of CT that received prenatal treatment in Quindío (*n* = 21). **E** Heat map of relative frequencies of cats negative for *Toxoplasma*, as determined by the University of Quindío (*n* = 109). **F** Heat map of relative frequency of cats positive for *Toxoplasma*, as determined by the University of Quindío (*n* = 25)

The only notable association among the variables in the Armenia study was that between *Toxoplasma* seropositivity and use of unboiled water/drinking tap water (Fig. 7A).

Among participants from La Universal, no variables except for age were significantly associated with IgG antibodies (Fig. 7B), and this significant relationship was lost in the multivariable linear regression. In Guaduales de la Villa, the habits of drinking bottled water and eating undercooked meat were negatively associated with infection. Variable representing eating undercooked meat was just coded “yes” as 1 and “no” as 0. The outcome variable was 1 if they were marked as positive in the IgG column of the dataset. This analyisis used a logistic regression in R with seropositivity as the outcome and *all* variables used as covariates. In this model, undercooked meat had a *p* value of 0.034. In an analysis where undercooked meat is the *only* predictor and other variables are not accounted for. However, it was not significant with a *p* value of 0.06. The total model as better adjusted for the effects of the other variables and the analyses in context of each other suggest that oocyst contamination of water is the most important source of infection, with both variables perhaps influenced by socioeconomic status Additionally, ocular lesions associated with *Toxoplasma* only occurred in people with IgG antibodies.

CT cases were mapped based on whether mothers had received prenatal treatment (Fig. 7C and D). Guaduales de la Villa showed relatively high rates of prenatal treatment within Armenia. Of the 8 points around that community, 7 had been treated (87.5%); meanwhile, only 21 of the 44 points with known treatment status in Armenia had received treatment (47.7%).

Figures 7E and F show the distribution of all cats in Armenia and distribution of cats based on *Toxoplasma* infection status. No significant relationship was found between the spatial distributions of IgG-negative and IgG-positive cats in Armenia.

## Discussion

Identifying specific higher-risk areas for toxoplasmosis enables more comprehensive prevention strategies that are tailored to sources of this disease. In Panama, our efforts to gather data for this end began with the 2014 HdN report, which estimated that seroprevalence of toxoplasmosis in Ciudad de Panamá could be as high as 50%. Later studies in 2016 and 2017 found high overall prevalence among pregnant women (about 22% and 44% IgG seropositivity, respectively), with consistent patterns of regional variability. For example, within the capital metropolitan area, townships with considerable poverty (such as Curundú) reached up to 24 times the average prevalence in Ciudad de Panamá. These findings were consistent with previous research that had associated greater seropositivity with living conditions that included dirt floors, open doors, and insect exposure [4•]. Both an increase in prevalence from western to eastern Panama and higher rates of disease in indigenous populations (although not to a statistically significant degree) were found in an initial retrospective study, a follow-up prospective study, and a longer-term aggregate study of 3500 women that used more sophisticated spatial epidemiology methods. More recent research, some of which has also examined regional seroprevalence in cats and dogs, has corroborated the trends found in our initial studies [11•, 12, 13•].

Out of several other findings, we noticed a strong association between seropositivity and proximity to water sources such as the Panama Canal; we are still investigating this correlation. Additionally, prevalence is high at the confluence of the Bayano and Mamoni rivers in the area of the Chepo water processing plant. From many possible risk factors, proximity to water would not have come to our attention if not for spatial epidemiological work in Colombia, a country where treatment for CT has reduced severe disease over the past 5 years and where water has been identified as a major route of *Toxoplasma* transmission [14•, 15]. Our study of risk factors in Quindío emphasized the correlation between proximity to bodies of water and issues such as congenital infection, retinal disease, and rates of lymphadenopathy related to toxoplasmosis. Recent studies have confirmed the importance of water to transmission of *Toxoplasma* in Colombia [16•]. Future studies in either Panama or Colombia should also investigate well water, which was found to be a risk factor in a recent study of agrarian populations in Morocco [17•].

## Conclusion

Overall, these spatial epidemiological studies have helped identify populations and geographic areas in Panama and Colombia where efforts to screen pregnant women for *Toxoplasma* and to provide prompt treatment to prevent CT might have the greatest relative impact. Furthermore, this work has also informed Moossazadeh’s mathematical models of infection risk, which could guide future interventions in cases where detailed epidemiological data are not available. Although our goal is to make our screening, treatment, and education programs available throughout both countries of focus, identifying the highest-risk areas is an important, practical step in this process.

## Supplementary Material

1832240-Sup_Material
