# Supplementary material for: Building Programs to Eradicate Toxoplasmosis Part III: Epidemiology and Risk Factors
Source: Curr Pediatr Rep. Author manuscript; Available in PMC 2023 Sep 22. (PMC10516319; doi:10.1007/s40124-022-00265-0)
Supplement: 1832240-Sup_Material [file NIHMS1832240-supplement-1832240-Sup_Material.pdf]

Building Programs to Eradicate Toxoplasmosis

Mariangela Soberón Felín, JD,[1*] Kanix Wang, PhD,[2*] Andrew Grose,[3*] Zuleima Caballero, PhD,[4*] Ximena Norero, MD,[5*] Dora Estripeaut, MD,[5*] David Ellis, MD,[5] Catalina Raggi,[6,7] Catherine Castro, MD,[3,6*] Davina Moossazadeh,[6-8,10*] Margarita Ramirez,[6,7,9*] Abhinav Pandey,[6,7,9*] Aliya Moreira,[6,7,9*] Kevin Ashi,[3] Samantha Dovgin,[6,7] Ashtyn Dixon,[6*] Xuan Li, MD,[10*] Fatima Alibana Clouser, MD,[6] Ian Begeman,[3,6] Sharon Heichmann, MD,[3,6*] Joseph Lykins, MD,[3,6] Delba Villavos,[4] Lorena Fabrega,[4] José Sanchez,[6,7] Connie Mendivil,[4] Mario L. Quijada, BSc.,[4] Silvia Fernández-Pirla,[1,11] Digna Wong, MD,[4] Mayrenis Ladrón de Guevara, MD,[4] Carlos Flores,[12], Jovanna Borace,[12] Anabel García, DVM,[4] Natividad Caballero, MD,[13] Claudia Rengifo-Herrera, DVM,[14] Stephanie Ross,[10] Mimansa Dogra,[6] Vishan Dhamsania, MD,[3,15] Nicholas Graves, MD,[3,15] Marci Kirchberg, MS,[15,16] Kopal Mathur, MS,[15,16] Ashley Aue,[16] Arturo Rebollon, MD,[17] Kenneth Boyer, MD,[10] Peter Heydemann, MD,[10] A. Gwendolyn Noble, MD, PhD,[18] Peter Rabiah, MD,[19] Shawn Withers,[15] Chunlei Su, PhD,[20] Daniel Vitor Vasconcuelos Santos, MD,[21] Alcibiades Villareal,[4] Ambar Perez,[4] Carlos Andrés Naranjo Galvis,[22] Mónica Vargas,[23] Laura Lorena Lopez,[23] Guillermo Padrieu,[24] Alejandra de la Torre, MD, PhD,[25] Daniel Nicolae PhD,[8,10] Andrey Rzhetsky, PhD,[2] Eileen Stillwaggon, PhD,[26] Larry Sawers, PhD,[27] Francois Peyon, MD, PhD,[28] Gabrielle Britton, PhD,[4] Jorge Motta, MD, MPH,[29] Jorge Gómez-Marín, MD, PhD,[23] Jagantha Rao, PhD,[4] Xavier Sáez Llorenz, MD,[5+] Osvaldo Reyes, MD[12+], Rima McLeod, MD, FACP, FIDSA[1-3,6,30,31+]

[1] Toxoplasmosis Programs and Initiatives in Panama, Ciudad de Panamá, Panama
[2] Institute for Genomics and Systems Biology, The University of Chicago, Chicago, Illinois
[3] BSD,Pritzker School of Medicine, The University of Chicago, Chicago, Illinois
[4] Instituto de Investigaciones Científicas y Servicios de Alta Tecnología AIP (INDICASAT-AIP), Ciudad de Panamá, Panama
[5] Department of Pediatrics Infectious Diseases/Department of Neonatology, Hospital del Niño, Ciudad de Panamá, Panama
[6] Department of Ophthalmology and Visual Sciences, The University of Chicago, Chicago, Illinois
[7] The College, The University of Chicago, Chicago, Illinois
[8] Department of Statistics, The University of Chicago, Chicago, Illinois
[9] The Global Health Center, The University of Chicago, Chicago, Illinois
[10] Rush University Medical School/Rush University Medical Center, Chicago, Illinois
[11] Academia Interamericana de Panamá, Ciudad de Panamá, Panama
[12] Hospital Santo Tomás, Ciudad de Panamá, Panama
[13] Hospital San Miguel Arcángel, Ciudad de Panamá, Panama

[14] Universidad de Panamá, Ciudad de Panamá, Panama
[15] Capstone Program,Center for Global Health, ,The University of Chicago, Chicago, Illinois
[16] Harris School of Public Policy, The University of Chicago, Chicago, Illinois
[17] Sanofi Aventis de Panamá S.A., Ciudad de Panamá, Panama
[18] Northwestern University Feinberg School of Medicine, Chicago, Illinois
[19] NorthShore Evanston Hospital, Evanston, Illinois
[20] Department of Microbiology, The University of Tennessee, Knoxville, Tennessee
[21] Universidade Federal de Minas Gerais, Minas Gerais, Brazil
[22] Universidad Autónoma de Manizales, Manizales, Colombia
[23] Universidad del Quindío, Armenia, Colombia
[24] The University of South Florida College of Public Health, Tampa, Florida
[25] Grupo de Investigación en Neurociencias, Universidad del Rosario, Bogotá, Colombia
[26] Department of Economics, Gettysburg College, Gettysburg, Pennsylvania
[27] Department of Economics, American University, Washington, D.C.
[28] Institut des agents infectieux, Hôpital de la Croix-Rousse, Lyon, France
[29] Secretaría Nacional de Ciencia, Tecnología e Innovación (SENACYT), Ciudad de Panamá, Panama
[30] Toxoplasmosis Center, The University of Chicago, Chicago, Illinois
[31] Department of Pediatrics (Infectious Diseases), The University of Chicago, Chicago, Illinois

[+]To Whom Correspondence Should be Addressed

rmcleod@uchicago.edu

xsaezll@cwpanama.net

oreyespanama@yahoo.es

jrao@indicasat.org.pa

Supplement: compilation of contributors' original research, writing, and presentations

<u>Authors' note:</u> Most of the materials in this supplement come from independent investigations conducted by students who were affiliated with global health research programs at the University of Chicago. As more student contributors were graciously invited to work with individuals and institutions in Panama and Colombia, the research that these students completed and presented became part of a truly international public health initiative, one that quickly involved more institutions and collaborators than many of us had originally conceived. None of these projects would have been possible without the collaboration of numerous U.S. and in-country partners. As such, each contributor's principal partners are highlighted in the title page of each section in this supplement.

WANG, KANIX ET AL. 1
Screening and seroprevalence maps for Panama
Developed in 2015-16 through the University of Chicago 2

MOREIRA & PANDEY ET AL. 5
Investigating Social and Infrastructural Parameters (poster)
Presented in 2016 to the University of Chicago Center for Global Health 6
Excerpt of manuscript (same title) 7
Excerpt of presentation (same title) 20

MOOSSAZADEH, DAVINA ET AL. 30
Risk Factors for *Toxoplasma gondii* IgG Seropositivity in Panama (poster)
Presented in 2017 to the University of Chicago Center for Global Health 31
A Model for Predicting *Toxoplasma gondii* IgG Seropositivity (manuscript) 32
Risk Factors for *Toxoplasma gondii* IgG Seropositivity (presentation) 60

RAMIREZ, MARGARITA ET AL. 78
An Analysis of Gestational Screening for Toxoplasmosis in Panama (poster)
Presented in 2017 to the University of Chicago Center for Global Health 79
Manuscript (same title) 80
Presentation (same title) 93

RAGGI, CATALINA ET AL. 107
A Spatial and Statistical Review and Comparison (poster)
Presented in 2019 to the University of Chicago Center for Global Health 108
Manuscript (same title) 109
Presentation (same title) 149

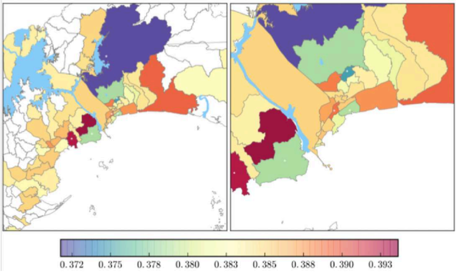

Kanix Wang, PhD
with Mariángela Soberón Felín, JD; Oswaldo Reyes, MD; Claudia Rengifo, PhD; Rima McLeod, MD

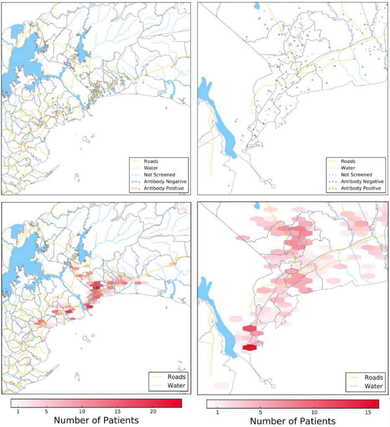

Figure 6: Scatter hexbin map of our data collection pattern. We identified a large portion of patients located along major roads. The grey dots represents patients not screened for toxoplasmosis, orange dots represent patients positive for infection and blue dots represent patient free of infection. Each hexbin summarizes the number of patient in the region. The maps also show water lines and major roads.

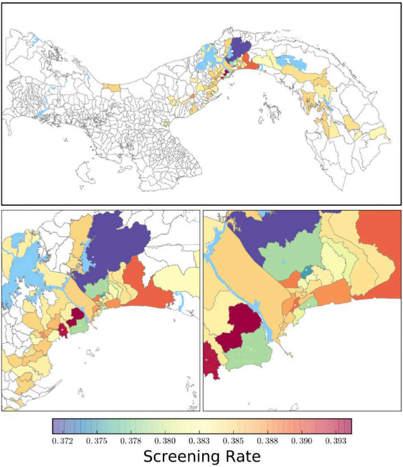

Figure 7: Screening map by Corregimientos. The screening rate is calculated using a Bayesian prior of Beta(254, 411) with mean at national average rate of 38.2% and updated based on data from each Corregimiento. Significant Corregimientos are marked with white asterisks.

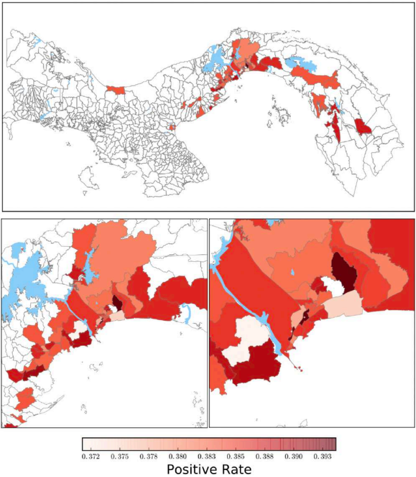

Figure 8: We generated infection rate map by Corregimientos. The positive rate is calculated using a Bayesian prior of Beta(63, 191) with mean at national average rate of 24.8% and updated based on data from each Corregimiento. Significant Corregimientos are marked with white asterisks.

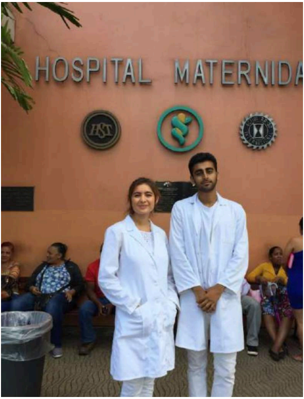

Aliya Moreira and Abhinav Pandey

with Rima McLeod, MD; Mariángela Soberón Felín, JD; Kanix Wang, PhD; Oswaldo Reyes, MD; and Hospital Santo Tomás – Panama; Hospital San Miguel – Panama

Investigating Social and Infrastructural Parameters Concerning Toxoplasmosis in Panama

Aliya Moreira, Abhi Pandey
The College at the University of Chicago

Introduction

Background

- Toxoplasmosis is a disease caused by the protozoan Toxoplasmosis gondii. It is the most prevalent parasite in the world, lying dormant in over 2,000,000,000 people.
- Cat oocysts are the major source of transmission, but humans can come into contact with them inadvertently through ingestion of contaminated water, dirt floors, undercooked meats, and improperly washed produce.
- Congenital infection can occur if a mother is infected for the first time during her term of pregnancy. This can cause serious problems for the fetus including brain and eye damage, and sometimes death.
- In Panama, factors like the tropical weather and high prevalence of feral cats contribute to the high incidence of toxoplasmosis in the country
- Panama's toxoplasmosis seroprevalence rate is 50%, one of the highest rates in the world.
- As a result of the high prevalence, in 2014 Panama passed a law requiring the screening of all pregnant women for toxoplasmosis as part of the standard prenatal care protocol.

Goal

- To differentiate areas of high incidence in order to examine potential contributing environmental differences.
- To determine which health centers and private clinics are and are not providing mandatory screening.

Objectives

- To measure the toxoplasmosis screening rates at public health centers and private clinics.
- To map incidence rates using data from those tested for the disease.
- To map screening rates using data from both those tested for the disease and those not tested for toxoplasmosis.

Methods

- Collected screening data, IgG/IgM test results, demographic data, and addresses from prenatal control charts (Figure 3) of pregnant women recruited from the maternity ward of Hospital Santo Tomas, the largest public hospital in Panama.
- Data was collected from a total of 665 pregnant women, of which 253 had been screened.
- Latitude and longitude was then determined from patient addresses in order to create both an incidence map and a screening map (Figures 1 and 2).
- Health center information collected from charts was used to investigate which health centers and private clinics were and were not providing mandatory screening.

Results

Incidence Map Results

- Indigenous people and people living east of the canal tend towards testing positive for toxoplasmosis.
- Certain townships (e.g. Curundu: 24x increase) have drastically greater rates of positivity.
- Number of acute women (14) is astonishingly high for a sample of 252.

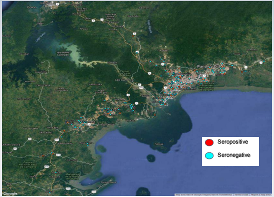

Figure 1. Incidence map

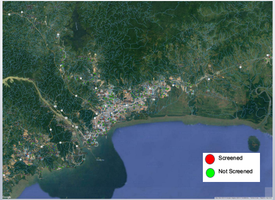

Figure 2. Screening map

Results (cont.)

Screening Map Results

- Location, race, and age are all significantly associated with screening.
- 30% of people are screened nationwide (individual townships vary between ~0% to ~60%).
- Pueblo Nuevo and Curundu have higher rates of screening, and also have greater incidence rates.

Health Center Investigation Results

- Private clinics screen their patients at a much higher rate than public health centers.
- Many doctors are unaware of mandatory screening laws.
- For some physicians toxoplasmosis is not a major health concern.
- There are inventory issues with toxoplasmosis test kits.

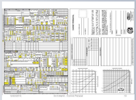

Figure 3. Sample Panamanian prenatal control chart.

Conclusions

- Incentivizes improvements in care for pregnant women and children
  - Only 30% of individuals are screened for toxoplasmosis.
- Provides useful information for the Panamanian Ministry of Health to enforce universal screening
  - Determined that the majority of health centers are not carrying out mandatory screening, and additionally that not all private clinics perform screening.
- Identifies key townships and demographics for future investigations
  - Curundu and Pueblo Nuevo have both high rates of screening and high rates of toxoplasmosis incidence.
- Identified 14 acute women who will receive immediate evaluation
  - This observed high rate of acute infection suggests that screening is not occurring until symptoms present during the pregnancy.

Ackowledgements

- Rima McLeod, M.D., Dept. of Ophthamology and Center for Toxoplasmosis, University of Chicago Hospitals
- Mariangela Soberon Felin, Coordinator of Toxoplasmosis Programs in Panama
- Oswaldo Reyes, M.D., Dept. of Obstetrics and Gynecology, Hospital Santo Tomas

University of Chicago Center for Global Health
Summer Research Fellowship Written Report
September 25th 2016

Investigating Social and Infrastructural Parameters Concerning Toxoplasmosis in Panama -Part 2

ALIYA F. MOREIRA and ABHI PANDEY
University of Chicago

We present Part 2 of this study In this supplement to PART III on Spatial epidemiology and risk factors.

Part 1 of the Wang, Moreira et al study is presented as Supplemental Part A in Part II -Educational initiatives of this series of four articles about Building Programs to eliminate toxoplasmosis.

Overview

This report summarizes a two-project research study that took place in July and August of 2016. Researchers Aliya Moreira and Abhi Pandey traveled on a University of Chicago Center for Global Health Summer Research Fellowship to Panama. In Panama City, they worked at the maternity ward of the largest public hospital, Hospital Santo Tomas, and in Hospital San Miguel, to administer surveys to and collect data from pregnant patients. In order to target the high rates of toxoplasmosis incidence in Panama, their projects seek to:

1) Determine cost and impact-effective educational strategies to inform Panamanians about the risks of toxoplasmosis and the methods of transmission.
2) Examine the rates of screening that take place in the country's public health centers in order to facilitate the enforcement of universal screening; additionally to examine areas of high incidence in order to determine possible methods of transmission of the disease.

Abstract 2

In our study, we hoped to accomplish two different tasks: to enforce Panama's mandatory prenatal screening, and to determine environmental sources of infection in the country. In order to do so, we hoped to create a data map of toxoplasmosis incidence rates, as well as to determine screening rates at public health centers and private clinics. To accomplish these tasks, we spent 5 weeks collecting screening data, IgG/IgM test results, demographic information, and addresses from patient prenatal control sheets at Panama's largest public hospital, Hospital Santo Tomas. Data was collected from a total of 665 pregnant women. We found that 38% had received screening, with individual townships varying from between 20-60% screened and private clinics having a greater rate of screening than public health centers. Of those screened, indigenous people and those living east of the canal were more likely to test positive for toxoplasmosis. Additionally, positive points are clustered around roads and waterways. The results of this study provide useful information for the Panamanian Ministry of Health to enforce universal screening within the country's public health centers. Additionally, it identifies areas of high incidence for future study that could provide clues to methods of transmission of the disease within Panama.

Introduction 2

The protozoan Toxoplasmosis gondii is one of the most prevalent parasites in the world (McLeod Toxoplasmosis Research Institute [TRI] website). Cat oocysts are the major source of transmission, but h umans can come into contact with them inadvertently through ingestion of contaminated water, dirt floors, undercooked meats, and improperly washed produce (McLeod TRI). Infection is generally asymptomatic in nature, with the parasite lying dormant in over 2,000,000,000 people worldwide (McLeod TRI). However, congenital infection can occur if a mother is infected for the first time during her term of pregnancy. This can cause serious problems for the fetus including brain and eye damage, and sometimes death (McLeod TRI).

In Panama, factors like the tropical weather and high prevalence of feral cats contribute to the high incidence of toxoplasmosis in the country (McLeod TRI). Panama's toxoplasmosis seroprevalence rate is 50%, one of the highest rates in the world (McLeod TRI). As a result of the high prevalence, in 2014 Panama passed a law requiring the screening of all pregnant women for toxoplasmosis as part of the standard prenatal care protocol (Li et al 2016).

As a result of this information, our project had two objectives:

1. The first is to determine which health centers and private clinics are and are not providing mandatory screening. While the 2014 screening law is in place, the extent to which different health centers and public clinics are carrying out the screening has not been measured. Our objective then is to measure and map the toxoplasmosis screening rates at public health centers and private clinics. This information could potentially be used by the Panamanian Ministerio de Salud to facilitate the enforcement of the screening law in the specific health centers and private clinics that are not carrying out the mandatory precaution. Additionally, the map could potentially show areas of high and low screening prevalence to help determine

regions that are particularly underserved with regards to toxoplasmosis screening.

2. The second objective is to differentiate areas of high prevalence in order to examine potential contributing environmental differences. By determining the areas of high prevalence, it may be possible to determine reasons for these fluctuations.. For example, regions with large numbers of feral cats may be associated with regions of high prevalence. Alternatively, we may discover differences in water supplies or the common presence of dirt floors in different regions to be linked with toxoplasmosis. In order to find this, we will map the prevalence rates we discover onto a map of the country in order to most clearly see and analyze these fluctuations.

Methods 2

Participants

Our participant sample was a convenience sample consisting of 665 pregnant women in attendance at the Hospital Santo Tomas and Hospital San Miguel Arcangel in Panama City, Panama. Patient prenatal control cards were requested from patients located in the External Consult room on the first floor of Hospital Santo Tomas, where patients come for check-ups and emergency visits. On other floors and in Hospital San Miguel Arcangel, which contained in-patients whose prenatal control cards were in files and not carried with them, data was collected anonymously from the floor's central record space without interaction with the patient. Demographic information including age, race, educational status, and literacy was obtained from subjects.

Procedure

At Hospital Santo Tomas and Hospital San Miguel Arcangel, patient prenatal control cards (Figure 2) were accessed. Information was collected anonymously from the files. IgG/IgM test results, addresses, health center/private clinic names, and demographic data was collected from each patient. After data collection, patient addresses were used to determine longitude and latitude coordinates using Google Maps. Coordinates were used with screening data in order to create both an incidence map, using only data from patients who had been screened to show areas of high and low seropositivity, and a screening map, which used data from all patients that we had collected to show areas of high and low screening. In the incidence map, patients that tested positive for at least one tes (<20 week IgG, >20 week IgG, or IgM) were considered "positive."

Health center and public clinic names were used in conjunction with screening data in order to determine screening rates for each. For example, if we collected data from 50 patients of the San Isidro Health Center, and 14 of those patients had been tested for toxoplasmosis, then 14/50 or 28% would be the screening rate.

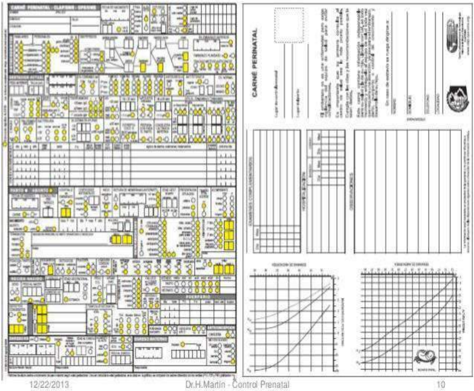

Figure 2. Sample prenatal control card. -larger representation is available in the presentations in the supplementary materials.

Results 2

Data was collected from a total of 665 pregnant women. Of that sample, 252 had been screened for toxoplasmosis. The overall screening rate that we determined was thus 38.9%.

Health Center Investigation Results

Screening rates in individual health centers and private clinics vary wildly from 0% to 100%. Overall, private clinics screen their patients at a significantly higher rate than public health centers.

Incidence Map Results

Based on statistical analysis of the data for our map (Figure 3), we found that people living to the east of the canal tend towards testing positive for toxoplasmosis. Additionally, certain townships have drastically increased rates of positivity. For example, the township of Curundu has a

twenty-four times greater rate of seropositivity. Furthermore, we identified from our demographic data that indigenous people were statistically more likely to test positive for *T.gondii*. Lastly, we observed a surprisingly high number of women who had serum IgM antibody specific to *T.gondii* with Roche tests—14 out of our 253 screened patients, or 5.6%.

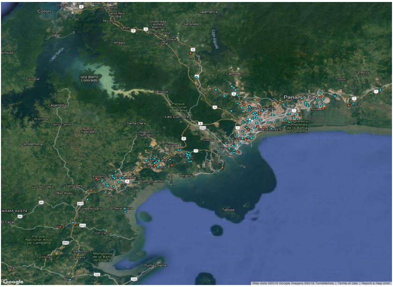

Figure 3. Incidence map, coordinates in red are seropositive and coordiantes in blue are seronegative.

Screening Map Results

Based on statistical analysis of the data for our map (Figure 4), we found that while 38.9% of people are screened nationwide, individual townships vary between 0% and about 60%. Specifically, the townships of Pueblo Nuevo and Curundu both have higher rates of screening. Additionally, from our demographic information we found that both race (caucasian) and age (older age) were significantly associated with screening. A variety of different IgG and IgM tests were used in community centers but at Hospital Santo Tomas testing was with the Roche automated test.

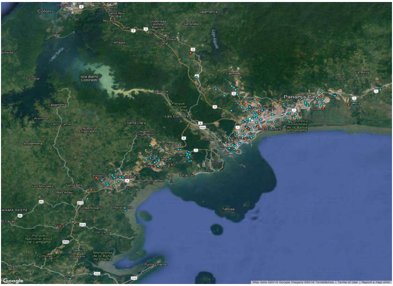

Figure 4. Screening map, coordinates in red have been screened and coordinates in green have not been screened for toxoplasmosis.

Discussion 2

The results of our study provide useful information to enforce improvements in the care of pregnant Panamanian patients and expose areas for future study. We hope to incentivize areas for improvement in care of pregnant women and children through exposing the lacking areas with our study. We found that only 38.9% of pregnant women are screened for toxoplasmosis, which certainly means that some acute infections go unnoticed and untreated. In a future study, we hope to take this data and run economic analysis to determine the cost-benefit ratio for the costs of the amount of babies that go undiagnosed compared to the cost of testing 100% of

pregnant mothers in order to incentivize improvements in care.

Additionally, our study provides useful information for the Panamanian Ministry of Health (MINSA) to aid in their enforcement of universal screening. We determined that the majority of health centers are not carrying out mandatory screening, and additionally that not even all private health clinics are carrying out testing in 100% of their patients. We have identified a huge socioeconomic disparity in access to care, and hope that MINSA will be able to utilize this information to work towards fixing this discrepancy. By identifying exactly which centers and clinics aren't following the 2014 screening law, we hope to aid MINSA in directing their enforcement efforts towards universal screening.

Furthermore, our study has identified key townships and demographics that are in need of future investigation. The indigenous community has a higher rate of seropositivity, and the reasons for this discrepancy could be revealing about potential methods of *T.gondii* transmission. such as exposure to soil and contaminated water transmission. Additionally, the township of Curundu specifically has both a higher screening rate and a higher prevalence rate. This may mean that Curundu specifically has environmental factors that predispose the area for high rates of toxoplasmosis, or that the statistics are more representative due to the higher rate of testing. Either way, the township of Curundu offers interesting potential for future investigation.

Lastly, we discovered 14 women with serum IgM antibody to *T. gondii* who will receive immediate evaluation. This observed very high rate of acute infection is unusual, but may suggest that screening is not occurring until symptoms present during pregnancy. From our data and conversations with Panamanian physicians at the hospitals and health centers, it was suggested that many doctors are unaware of mandatory screening laws, and that for some physicians toxoplasmosis is not a major health concern for their pregnant patients. Additionally, there are inventory issues with test regaents to detect *T.gondii* seropositivity.

test kits that may make physicians less likely to perform testing on all of their pregnant patients. Overall, we determined that the 2014 screening law is not being followed and identified empirical evidence for this lack in screening that can be presented to the Panamanian Ministry of Health to facilitate the enforcement of universal screening. Additionally, we identified key populations and townships—indigenous peoples and Curundu—that may provide useful information about toxoplasmosis transmission upon future study.

Appendix

FIGURE 5

Statistical analysis of prevalence map data (Figure

Coefficients:

	Estimate	Std. Error	z value	Pr(>|z|)	
(Intercept)	117.15351	54.17331	2.163	0.0306	*
Latitude	-0.64537	1.11384	-0.579	0.5623	
Longitude	1.42946	0.63725	2.243	0.0249	*
Age	0.01024	0.02536	0.404	0.6863	
RaceIndigena	1.87399	1.29802	1.444	0.1488	

FIGURE 6

Statistical analysis of screening map data (Figure 4).

	Estimate	Std. Error	z value	Pr(>|z|)	
(Intercept)	4.226e+00	6.557e+02	0.006	0.994858	
Latitude	-2.116e+00	6.105e-01	-3.465	0.000529	***
Longitude	5.119e-04	3.307e-01	0.002	0.998765	
Age	3.394e-02	1.531e-02	2.217	0.026653	*
RaceIndigena	-2.011e+00	6.243e-01	-3.221	0.001276	**
RaceMestiza	-5.491e-01	2.591e-01	-2.119	0.034103	*
RaceNegra	-5.373e-01	3.790e-01	-1.418	0.156331	
RaceOtra	-1.934e+00	8.111e-01	-2.385	0.017097	*

References

1. Li, Xuan, Mariangela Felin Soberon, Lillian Bodden MD, Kenneth Boyer M., Rima McLeod, and Osvaldo Reyes. "Toxoplasmosis Education for Pregnant Women in Panama." *Obstetrics & Gynecology* 127.5 (2016): 15S-6S. Web.

2. McLeod, Rima. "An Overview of Toxoplasmosis: Cause, Prevalence, and Consequences." *TRI: Information about Toxoplasmosis*. Toxoplasmosis Research Institute and Center, n.d. Web. 15 Sept. 2016.

3. Sousa, O.E., R.E. Saenz, J.K. Frenkel. "Toxoplasmosis in Panama: A 10-year study." *The American Journal of Tropical Medicine* 38 (1988), pp. 315-322

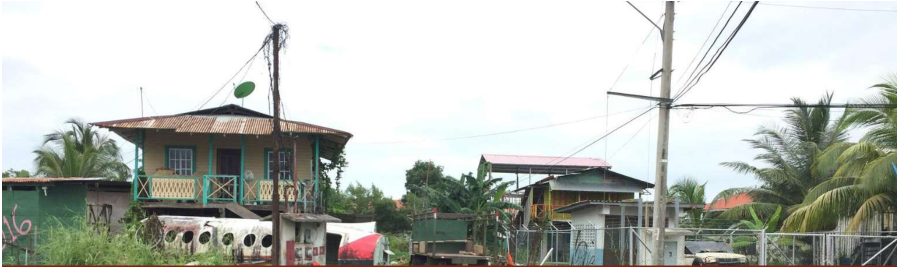

Investigating Social and Infrastructural Parameters Concerning Toxoplasmosis in Panama

Researchers: Abhinav Pandey, Fourth Year, Economics B.A. // Aliya Moreira, Fourth Year, Biology B.A. // Kanix Wang, Biology / Math B.A., PhD Candidate

Mentors: Rima McLeod, M.D., Dept. of Ophthalmology and Center for Toxoplasmosis, University of Chicago Hospitals, Oswaldo Reyes, M.D. Dept. of Obstetrics and Gynecology, Hospital Santo Tomas

THE UNIVERSITY OF CHICAGO | Center for Global Health

Toxoplasmosis Overview

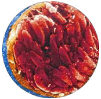

Spread by the protozoan *Toxoplasma gondii*

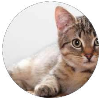

Cat oocysts: major source of transmission

33% Most prevalent parasite in the world, lying dormant in over 2,000,000,000 people.

50% Prevalence rate in Panama

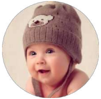

Congenital transmission can cause brain and eye damage

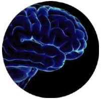

Seroprevalence associated with neurologic disease / seizures

Investigating Toxoplasmosis Screening Rates and Incidence

Objective: Map Seroprevalence Incidence and Screening Rate

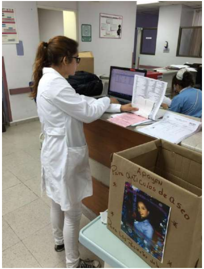

Collected screening data, IgG / IgM test results, demographic data, and addresses from prenatal control charts

Latitude and longitude determined from patient addresses to create both an incidence map and a screening map

Used health center information from charts to investigate which health centers were and were not providing mandatory screening

Incidence Maps

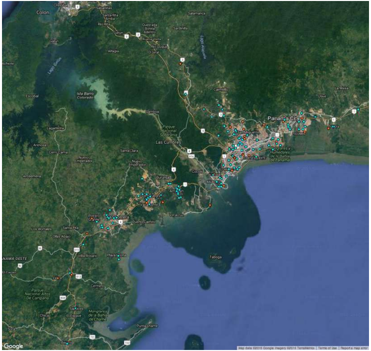

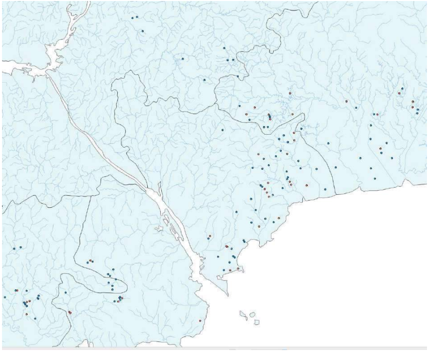

Key Findings

Indigenous people and people living east of the canal trend towards testing positive for toxoplasmosis

Certain townships (e.g. Curundu) have drastically greater rates of positivity

Number of acute women (14) is astonishingly high for a sample of 252

Screening Maps

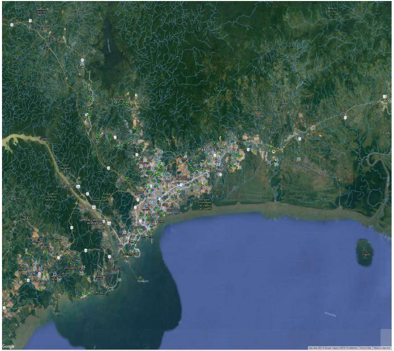

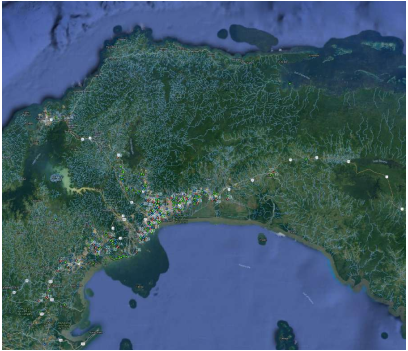

Key Findings

38% of people are screened nationwide (individual townships vary between ~20%-60%)

Private clinics have greater rates of screening than public clinics

Pueblo Nuevo, Curundu have higher rates of screening; also have greater incidence rates

Health Center Investigation Results

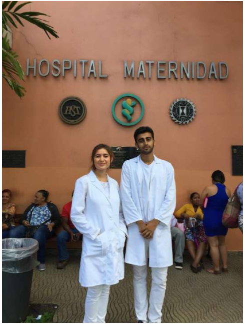

Many doctors unaware of mandatory screening laws

For some physicians toxoplasmosis is not a major health concern

There are inventory issues with toxoplasmosis test kits

Appendix B: Statistical Analysis - Screening

```
Deviance Residuals:
    Min       1Q   Median       3Q      Max
-1.8251  -0.9935  -0.7642   1.2188   2.1549

Coefficients:
                              Estimate Std. Error z value Pr(>|z|)
(Intercept)                  4.226e+00  6.557e+02   0.006 0.994858
Latitude                    -2.116e+00  6.105e-01  -3.465 0.000529 ***
Longitude                    5.119e-04  3.307e-01   0.002 0.998765
Age                          3.394e-02  1.531e-02   2.217 0.026653 *
RaceIndigena                -2.011e+00  6.243e-01  -3.221 0.001276 **
RaceMestiza                 -5.491e-01  2.591e-01  -2.119 0.034103 *
RaceNegra                   -5.373e-01  3.790e-01  -1.418 0.156331
RaceOtra                    -1.934e+00  8.111e-01  -2.385 0.017097 *
LiteracyTRUE                -1.128e-01  5.468e-01  -0.206 0.836608
EducationPrimaria            1.417e+01  6.551e+02   0.022 0.982738
EducationSecundaria          1.449e+01  6.551e+02   0.022 0.982347
EducationSegundo            -4.881e-01  1.213e+03   0.000 0.999679
EducationUniversidad         1.472e+01  6.551e+02   0.022 0.982068
Marital.StatusOtro           2.392e-01  1.445e+00   0.166 0.868475
Marital.StatusSoltera       -9.967e-02  3.635e-01  -0.274 0.783968
Marital.StatusUnion Estable -4.084e-01  2.871e-01  -1.423 0.154824
```

Appendix C: Statistical Analysis - Incidence

Deviance Residuals:

Min	1Q	Median	3Q	Max
-1.7405	-0.7937	-0.6650	0.8211	2.1158

Coefficients:

	Estimate	Std. Error	z value	Pr(>|z|)	
(Intercept)	117.15351	54.17331	2.163	0.0306	*
Latitude	-0.64537	1.11384	-0.579	0.5623	
Longitude	1.42946	0.63725	2.243	0.0249	*
Age	0.01024	0.02536	0.404	0.6863	
RaceIndigena	1.87399	1.29802	1.444	0.1488	
RaceMestiza	0.27771	0.43173	0.643	0.5201	
RaceNegra	-1.01647	0.80216	-1.267	0.2051	
RaceOtra	1.12857	1.49056	0.757	0.4490	
LiteracyTRUE	0.78921	1.14630	0.688	0.4911	
EducationSecundaria	-0.35971	0.70818	-0.508	0.6115	
EducationUniversidad	-0.81810	0.74239	-1.102	0.2705	
Marital.StatusOtro	15.93221	882.74353	0.018	0.9856	
Marital.StatusSoltera	0.74149	0.63882	1.161	0.2458	
Marital.StatusUnion Estable	0.67282	0.51354	1.310	0.1901	

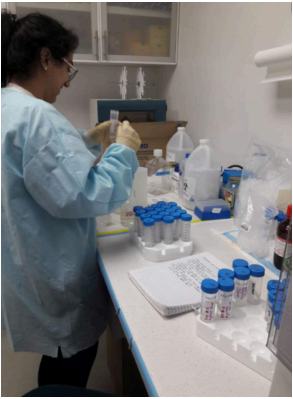

Davina Moossazadeh

with Margarita Ramirez; Zuleima Caballero, PhD; Claudia Rengifo, PhD; Catherine Castro; Mariángela Soberón Felín, JD; Rima McLeod, MD; and
Secretaría Nacional de Ciencia, Tecnología e Innovación (SENACYT) – Panama; Hospital Santo Tomás – Panama

Risk Factors for *Toxoplasma gondii* IgG Seropositivity in Panama

Davina Moossazadeh[1], Margarita Ramirez[1], Zuleima Caballero PHD[2], Mariangela Soberón Felin JD[2], and Rima McLeod MD [3,4]
[1] The University of Chicago, [2] INDICASAT AIP, [3] Department of Ophthalmology and Visual Sciences, [4] Section of Infectious Diseases, Department of Pediatrics, Institute of Genomics, Genetics, and Systems Biology, Global Health Center, Toxoplasmosis Center, the Center for Health and the Social Sciences, CHeSS, the College, University of Chicago, Chicago, Illinois

Background

- Toxoplasmosis is a disease caused by the protozoan parasite *Toxoplasma gondi*
- The disease may be transmitted to the fetus when a woman acquires it for the first time during pregnancy, in which case it may result in ocular and neurological damage, as well as miscarriage, in the fetus
- Infection before conception protects the fetus
- IgG persists for life, so seropositivity indicates prior infection
- An IgG test can be used to screen out pregnant women with minimal risk of vertical transmission
- There are no published results on the seroprevalence of T. gondii in Panama, although the neighboring countries of Colombia and Costa Rica have estimated seroprevalences of 43.1-66.7% and 49.2-60.8%, respectively
- This high regional seroprevalence suggests that *T. gondii* infection may pose a high risk to pregnant women in Panama

Specific Aims

- Assess risk factors for *T. gondii* IgG seropositivity among pregnant women in Panama

Methods

- Tested 343 pregnant women for *T. gondii* seropositivity at Hospital Santo Tomás
- Assessed pregnant women for risk factors via questionnaire
  - Demographics
  - Contact with animals
  - Food hygiene habits
  - Water source
- Pearson's chi-squared, Fisher's exact tests in R

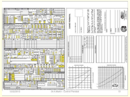

Figure 1: Medical control charts for pregnant women at Hospital Santo Tomás, including results of past laboratory tests.

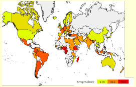

Figure 2: Map of global seropositivity for *T. gondii* adapted from data presented in Flegr *et al.*, 2014 [3]

Results

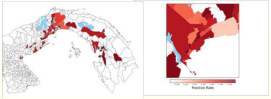

Figure 3: *T. gondii* IgG seropositivity rate among pregnant women in Panama the previous summer (2016). The Curundú township shows an odds ratio of 24 as compared to the total.

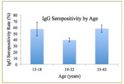

Figure 4: *T. gondii* IgG seropositivity rates among pregnant women by maternal age group ($p < 0.05$). Error bars represent the standard error of the mean.

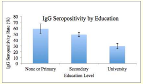

Figure 5: *T. gondii* IgG seropositivity rates among pregnant women by maternal education level ($p < 0.05$). Error bars represent the standard error of the mean.

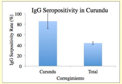

Figure 6: *T. gondii* IgG seropositivity rate among pregnant women in the Curundú township versus the overall seropositivity rate ($p < 0.05$). Error bars represent the standard error of the mean.

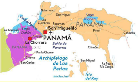

Figure 7: *T. gondii* IgG seropositivity rate among pregnant women increases from 35.0% in Panamá Oeste (West Panama) province to 36.7% in Panamá Province ($p = 0.09$).

Conclusions

- Concordance of young age and education with IgG seropositivity
- Lower socioeconomic status may be a risk factor
- However, IgG was found in all age groups and all education groups
- Absence of other significant risk factors suggests unidentified risk factors or insufficient sample size
- Therefore it is important to screen all women regardless of risk factor status

Limitations

- Self-reported data
- Multiple researchers obtaining data
- No centralized electronic medical record keeper
- No correcting for the autocorrelation between education and age

Acknowledgements

- Anabel García DVM, Connie Mendivil, Mario L Quijada R. BSc, Mayrene Ladrón de Guevara MD, Catherine Castro BA, Joseph Lykins MD, Kristen Wroblewski MS, Kanix Wang BA, Guillermo Pradieu BA
- National Secretary of Science, Technology and Innovation (SENACYT), Republic of Panama
- Hospital Santo Tomás
- The University of Chicago Center for Global Health, Jeff Metcalf Internship Program

References

[1] Barrera, Ana M., et al. "Toxoplasmosis adquirida durante el embarazo, en el Instituto Materno Infantil en Bogotá." Revista de Salud Pública 4.3 (2002): 286-293. [2] Castro, A. T., A. Congora, and M. E. Gonzalez. "Toxoplasma gondii antibody seroprevalence in pregnant women from Villavicencio, Colombia." Orinoquia 12.1 (2008): 91-100. [3] Flegr, Jaroslav, et al. "Toxoplasmosis–a global threat. Correlation of latent toxoplasmosis with specific disease burden in a set of 88 countries." *PloS one* 9.3 (2014): e90203. [4] Hill, D., and J. P. Dubey. "Toxoplasma gondii: transmission, diagnosis and prevention." Clinical microbiology and infection 8.10 (2002): 634-640. [5] Joynson, D. H. M., and E. C. Guy. "Laboratory diagnosis of toxoplasma infection." Toxoplasmosis. A Comprehensive Clinical Guide. Cambridge University Press, Cambridge, 2001. 296-318. [6] Pappas, Georgios, Nikos Roussos, and Matthew E. Falagas. "Toxoplasmosis snapshots: global status of Toxoplasma gondii seroprevalence and implications for pregnancy and congenital toxoplasmosis." International journal for parasitology 39.12 (2009): 1385-1394. [7] R Core Team (2015). R: A language and environment for statistical computing. R Foundation for Statistical Computing, Vienna, Austria. URL http://www.R-project.org/. [8] Rosso, Fernando, et al. "Prevalence of infection with Toxoplasma gondii among pregnant women in Cali, Colombia, South America." The American journal of tropical medicine and hygiene 78.3 (2008): 504-508. [9] Wilson, Christopher B., et al. "Development of adverse sequelae in children born with subclinical congenital Toxoplasma infection." Pediatrics 66.5 (1980): 767-774. [10] Zapata, M., L. Reyes, and I. Holst. "Decreased prevalence of Toxoplasma gondii antibodies in adults form the Central Valley in Costa Rica." Parasitol Latinoam 60 (2005): 32-7.

A Model for Predicting *Toxoplasma gondii* IgG Seropositivity Among Pregnant Women in Panama

A thesis submitted to the Department of Statistics in partial fulfillment of the requirements for a Bachelor's degree with Honors

Davina Moossazadeh

Advisor: Dan Nicolae, PhD

June 4, 2018

Abstract

The research objective of this study was to assess risk factors for *Toxoplasma gondii* IgG seropositivity among pregnant women seeking care at Hospital Santo Tomás in Panama, in order to develop a predictive model for risk of exposure to *T. gondii* among this population. Univariate analyses found age squared (logistic regression, $p = 0.018$), education level (χ^2 test, $p = 0.0008$), and longitude of home (logistic regression, $p = 0.004$) to be significantly associated with seropositivity; however, age squared is no longer significant when controlling for the effects of education. A Lasso regression predicted longitude, distance from water, education, pet dogs, wild animal contact, consumption at food stands and restaurants, street dogs, consumption of raw meat, age, and pet cats to be the most important factors in assessing for IgG status. Between a logistic regression (of IgG status on age and education) and the Lasso regression, both models have high accuracy rates (68% for logistic and 69% for Lasso regression) and low false positive rates (11%, 14%, respectively). The main source of error is in the false negative rate, since the majority of patients are IgG seronegative. In this respect, Lasso performs better than logistic regression, with a lower false negative rate (56% versus 64%), which also produces a lower mean error (31% versus 43%). Therefore, the Lasso model is more accurate than logistic regression in determining IgG status and is more sensitive in detecting patient seropositivity; however, this model needs to be fine-tuned for further increased sensitivity.

Introduction

Toxoplasmosis is a disease caused by the protozoan parasite *Toxoplasma gondii* (Hill & Dubey 2002). The parasite may be acquired by ingestion of tissue cysts found in raw or undercooked meat, or by ingestion of oocysts from cat feces found in contaminated food or water. While often asymptomatic, the disease can become activated in immunocompromised individuals, in whom it most commonly causes toxoplasmic encephalitis (Araujo & Remington 1987, Luft & Hafner 1990). Toxoplasmosis may also be acquired congenitally: the parasite tachyzoites may be transmitted transplacentally to the fetus when a woman contracts the parasite

for the first time during pregnancy (Tentner *et al.* 2001). In this case, infection may result in ocular and neurologic deficits in the fetus — such as chorioretinitis, loss of sight, psychomotor impediments, seizures, microcephaly, hydrocephalus, and intracranial calcifications, among other manifestations — as well as miscarriage (Wilson *et al.* 1980).

Infection of the mother before conception protects the fetus. Congenital toxoplasmosis is almost always contracted from a mother who has an acute infection during pregnancy, which is signalled by the presence of IgM antibodies. At about 8 weeks after infection, IgM antibody levels usually decrease, while IgG levels become high and stable, in a test called the Sabin Feldman dye test or certain other IgG tests. This signals subacute/chronic infection, indicating that the individual is now likely to be immune to subsequent infection. With established maternal immune response, the fetus is much less likely to be at risk of contracting congenital toxoplasmosis (Joynston & Guy 2001, Hall 1992). IgG antibodies persist for life, so their presence in serum is a marker of prior infection with the parasite. Therefore, a test for IgG seropositivity, without other markers of acute infection, is useful in identifying pregnant women with minimal risk of vertical transmission to the fetus, which is especially important in prenatal screening programs.

There are no published results on the seroprevalence of *T. gondii* in Panama, although unpublished data from 2014 have shown that 50% of Panamanian women of childbearing age cared for at Hospital Santo Tomás, Panama's largest public hospital, were IgG positive (Sáez Llorens *et al.*, unpublished). The neighboring countries of Colombia and Costa Rica have estimated seroprevalences of 43.1-66.7% and 49.2-60.8%, respectively (Pappas *et al.* 2009, Rosso *et al.* 2008, Castro *et al.* 2008, Barrera *et al.* 2002, Zapata *et al.* 2005). This high regional seroprevalence suggests the possibility of similarly high rates in Panama. The high environmental contamination and the sizable proportion of women who are IgG seronegative — and thus at risk — suggest that *T. gondii* infection may pose a high risk to pregnant women in Panama and their fetuses.

Previous studies have identified soil floors, dogs, and cats in the home as risk factors for *T. gondii* antibody seropositivity in specific populations in Panama (Etheredge *et al.*, 2004). Furthermore, a retrospective study similar to the present one, conducted in Panama in 2016, found that geography (e.g., residence in San Miguelito or in more eastward areas) was a significant risk factor for IgG seropositivity (Wang, Pandey, &

Moreira, unpublished). Certain townships (*corregimientos*) had significantly high seroprevalences; the two with the highest seroprevalences were Curundú and Pueblo Nuevo, which saw 24-fold and tenfold increases, respectively, from the average seroprevalence across all townships. Furthermore, in Curundú specifically, cases of IgG seropositivity were clustered around the river, indicating that water source may be a risk factor. This study also saw a significant general trend of increasing seropositivity from the western end to the eastern end of the country. This result has been corroborated by similar data in domestic dogs and cats in Panama, which also show a significant increasing trend in seroprevalence from west to east (Rengifo-Herrera *et al.*, 2017).

The research objective of this current study was to assess risk factors for *T. gondii* IgG seropositivity among pregnant women seeking care at Hospital Santo Tomás in Panama, in order to develop a predictive model for risk of exposure to *T. gondii* among this population.

Methods & Dataset

In this study, we prospectively assessed 341 pregnant women at Hospital Santo Tomás; this is Panama's largest public hospital, which treats patients from all provinces of the country and sees approximately 19,000 deliveries per year — or about 50 per day. Patients were selected as they came in to the hospital maternity ward to attend pre-delivery appointments for ultrasound, c-section consultation, and high risk management (for patients with diabetes, hypertension, and other complications). Patients who had been tested for *T. gondii* antibodies in the two weeks prior to their visit were excluded.

Sera from selected patients were tested for *T. gondii* IgG seropositivity with Rche assay. Additionally, we assessed both seronegative and seropositive patients for various risk factors, including demographic information, contact with wild animals and pets, food hygiene and cooking habits, and sources of food and water (Table 1). These questions were based on previously identified risk factors (Etheredge *et al.*, 2004). Additionally, any past laboratory test results were obtained from each woman's medical control chart (Figure 1). All statistical tests and analyses were performed in RStudio (Version 3.1.3, Vienna, 2015).

Category	Variable	Type	Outcomes
Response	IgG	Response, Binary	Negative: ≤ 9.0 IU/mL (n=191)<br>Positive: ≥ 12.0 IU/mL (n=150)
Demographics	Maternal Age	Integer	Range: 15, 43
	Education level completed	Ordinal	None (n=2)<br>Primary (n=33)<br>Secondary (n=201)<br>University (n=99)
Location	Home Location	Binary	Urban (n=302)<br>Rural (n=37)
	Home Coordinates	Continuous, two-dimensional	Latitude (range: 7.58, 9.36), longitude (range: -78.18, -81.88)
Animal contact	Pets	Binary	Dogs Cats Other<br>No n=178 n=299 n=308<br>Yes n=157 n=35 n=25
	Contact with wild animals	Binary	No (n=316)<br>Yes (n=21)
	Street animals near home or work	Binary	Dogs Cats Pigeons Other<br>No n=163 n=157 n=271 n=296<br>Yes n=142 n=147 n=31 n=8
Diet	Consumption of street food	Ordinal	Never (n=42)<br>Sometimes (n=232)<br>Frequently (n=40)<br>Always (n=16)
	Frequent* food sources	Binary	Home Restaurants Food stands Walking vendors<br>No n=35 n=241 n=297 n=325<br>Yes n=303 n=97 n=41 n=13
	Frequent* meat consumption	Ordinal	Pig Chicken Beef Seafood Other<br>No n=292 n=55 n=219 n=280 n=336<br>Yes n=47 n=284 n=120 n=59 n=4
	Consumption of raw meat	Ordinal	Never (n=250)<br>Sometimes (n=49)<br>Frequently (n=8)<br>Always (n=20)
Hygiene	Washing hands before eating	Ordinal	Never (n=1)<br>Sometimes (n=45)<br>Frequently (n=79)<br>Always (n=214)
	Washing produce before consumption	Ordinal	Never (n=2)<br>Sometimes (n=12)<br>Frequently (n=28)<br>Always (n=294)
Water	Water source	Binary	Aqueduct (n=329)<br>Other (n=12)

Table 1: Description of the variables in the dataset. *Frequent is defined as at least a couple of times per week.

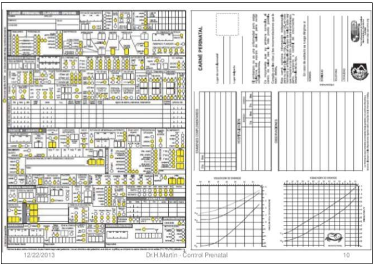

Figure 1: Prenatal medical control chart for pregnant woman at Hospital Santo Tomás, displaying results of past laboratory tests. Included are results of past *T. gondii* IgG and IgM antibody tests, along with avidity test results if the antibody test returned positive.

Results & Model

This section details the results of various tests used to determine association between *T. gondii* IgG seropositivity and the risk factors detailed in Table 1. From the results of these tests, a model was built to predict the probability of IgG seropositivity for a pregnant woman in Panama based on the relevant risk factors. In this section, 0 indicates seronegative and 1 indicates seropositive. The variables are defined as follows:

p = probability of seropositivity (defined by frequency)
a = age (years)
e = education level
lat = latitude
$long$ = longitude

Demographics

A plot of the proportion of IgG-positive patients against age exhibits a quadratic curve (Figure 2b), with the youngest and oldest age groups having highest seroprevalence. This is supported by the fact that mean age of the seronegative and seropositive groups do not differ significantly — 27.1 years for seronegative and 27.5 years for seropositive (t-test, $p = 0.63$) — while the variance of age for each of these groups does exhibit a slightly significant difference — 33.6 years^2 for seronegative and 45.6 years^2 for seropositive (F test, $p = 0.05$, Figure 2a).

A logistic regression of IgG status against age and age squared yields the following formula, where both variable coefficients are significant ($p = 0.024$, 0.020, respectively): $logit(p) = 4.1 - 0.3a + 0.006a^2$.

In order to correct for multicollinearity between age and age squared, the age variable was centered around its mean (27.3 years) before squaring, and these new centered variables for age and age squared were used instead for the regression. This yielded the following equation: $logit(p) = -0.5 - 0.0006(a - 27.3) + 0.006(a - 27.3)^2$. The coefficient for age squared is significant ($p = 0.020$), while the coefficient for age is not ($p = 0.97$).

Finally, age was removed from the regression model since it did not contribute significantly. This yielded the following formula, where the coefficient for age squared is significant ($p = 0.018$):

$$logit(p) = -0.5 + 0.006(a - 27.3)^2 \text{ (Figure 2b).}$$

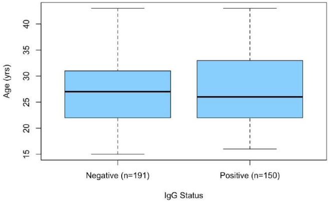

Figure 2a: Boxplot of age versus IgG status. The black bar represents the median, the blue box represents the 25th-75th percentiles, and the whiskers represent the minimum and maximum values for each group.

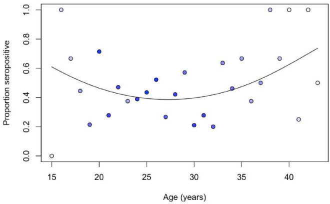

Figure 2b: *T. gondii* IgG seropositivity rates among pregnant women by maternal age, plotted with age centered around the mean. The curve represents the fit from the logistic regression of IgG status against age squared, with age centered around its mean. The points are shaded according to the number of women at each age, where darker colors signify a greater number of women, with white representing 1 woman and the darkest blue representing 23 women.

Combining the groups with no education and primary education for increased power, a χ^2 analysis of IgG status against education level yields a significant relationship between the two (p = 0.0008). We find that seroprevalence decreases with increasing education level (Figure 3).

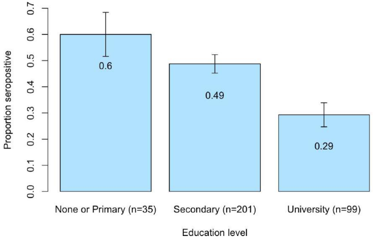

Figure 3: *T. gondii* IgG seropositivity rates among pregnant women by education level. Error bars represent the standard error of the mean.

A linear regression was used to assess for multicollinearity between the two demographic variables, age and education. The education levels were coded as follows: “none” or “primary” = 1, “secondary” = 2, “university” = 3. A plot of average education level against age exhibits a quadratic curve (Figure 4b), with the middle education level (secondary) having highest seroprevalence. This is supported by the fact that a one-way ANOVA shows no significant difference between the means of the three groups (although they are close: p = 0.072), while a

Brown-Forsythe test shows significant differences in the variances (p = 0.017, Figure 4a). The Brown-Forsythe test is a test for equality of variances, executed by the leveneTest function in the lawstat library; the test statistic is the F statistic from a one-way ANOVA on $|y_{ij} - \overline{y}_j|$.

A linear regression of education level against age and age squared yields the following formula: $E[e|a] = 0.4 + 0.1a - 0.002a^2$. The coefficients for both age and age squared are significant (p = 0.0020, 0.0028, respectively).

As with the regression of IgG seropositivity on age, age was then centered around its mean in order to correct for multicollinearity between age and age squared. This yielded the following formula: $E[e|a] = 2.3 + 0.009(a - 27.3) - 0.002(a - 27.3)^2$. The coefficient for age squared is significant (p = 0.0028), while the coefficient for age is not (p = 0.10).

Since, as with the regression of IgG seropositivity against age, the coefficient for age did not return significant, it was removed from the model, yielding the following equation, where the variable coefficient is significant (p = 0.0069, Figure 4b):

$$E[e|a] = 2.3 - 0.002(a - 27.3)^2.$$

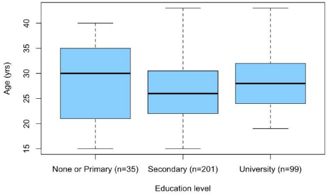

Figure 4a: Boxplot of age versus education level. The black bar represents the median, the blue box represents the 25th-75th percentiles, and the whiskers represent the minimum and maximum values for each group.

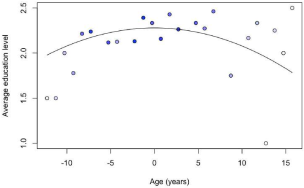

Figure 4b: Average education level among pregnant women by maternal age, plotted with age centered around the mean. The curve represents the fit from the linear regression of education against age squared. Education level 1 corresponds to no school or primary level school, level 2 to secondary, and level 3 to university. The points are shaded according to the number of women at each age, where darker colors signify a greater number of women, with white representing 1 woman and the darkest blue representing 23 women.

Finally, the age and education variables were combined into a single logistic regression with IgG status as the response variable. First, IgG status was regressed against age, age squared, and education. This yielded the following formula: $logit(p) = 4.6 - 0.3a + 0.005a^2 - 0.7e$, where the coefficient on education is significant (p = 0.0003), while the coefficients on age and age squared are close to significant but not quite (p = 0.09, 0.07, respectively). The age variable was removed from the regression as it had the highest p value. This yielded the following formula: $logit(p) = 1.2 + 0.0003a^2 - 0.8e$, where the coefficient on education is significant (p = 0.0001), while the coefficient on age squared is not (p = 0.4). A logistic regression of IgG status on the interaction term between age and education similarly found that this term is not significant (p = 0.1).

The same logistic regression was then performed on the age and education variables, with age centered around its mean to account for multicollinearity between age and age squared, as was done with the regressions of IgG status and education on the age variable. First, IgG status was regressed against the centered age and age squared variables, along with education, yielding the following formula: $logit(p) = 1.6 + 0.005(a - 27.3) + 0.005(a - 27.3)^2 - 0.7e$, where the coefficient on education is significant (p = 0.0003), while the coefficients on age and age squared are not, although age squared is close (p = 0.8, 0.07, respectively). Again, age was removed from the regression, as it had the highest p value, yielding the following formula: $logit(p) = 1.1 + 0.005(a - 27.3)^2 - 0.7e$, where the coefficient on education is significant (p = 0.0003), while the coefficient on age squared is closer to significant but still not quite there (p = 0.06). A regression of IgG status on the interaction term between age squared and education again found that this term is not significant (p = 0.2). Therefore, age and age squared were removed from the model altogether, yielding the following combined formula of IgG seroprevalence on demographic factors, where the variable coefficient is significant (p = 0.0003):

$$logit(p) = 1.3 - 0.7e.$$

Location

A χ^2 analysis of IgG status versus home location (urban or rural) did not yield significant results (p = 0.82).

To assess the effects of home coordinates on exposure risk, all latitude and longitude data were standardized using the tidyr package. The coordinates were then converted from degree minute second format to decimal degree format. This was done using the measurements::conv_unit function in R, which employs the following transformation: $decimal\ degrees = \frac{minutes}{60} + \frac{seconds}{3600}$. The coordinates were then normalized so that the (longitude, latitude) coordinate (0,0) represented the coordinates of Hospital Santo Tomás, actually (-79.53, 8.97), for ease of calculation. No scalar or sign transformation was applied, so a unit increase in the coordinates normalized around the hospital corresponds to an equal increase in the actual decimal degree coordinates. Because Panama is to the north of the Equator and west of the Prime Meridian, increases in these latitude and longitude values correspond to movements to the north and west, respectively. All distances between points were calculated by taking the Euclidean distance between the actual decimal degree coordinates, i.e., $distance = \sqrt{(lat_1 - lat_2)^2 + (long_1 - long_2)^2}$.

Three sets of explanatory variables were analyzed. First, distance from the hospital was analyzed in order to reduce the dimensionality of latitude and longitude to a single dimension; this is based on the assumption that farther distance from the hospital would represent farther distance from the city center, as the hospital is at the heart of the city. Second, distance from the nearest coast was analyzed for the same purposes of dimension reduction, based on the hypothesis that ocean water may be a vector for the parasite. The data points for Panama's coastal borders were retrieved from combining data points from the "world" database (Becker & Wilks 1995) with those from richardcondit.org (Index). Third, latitude and longitude were analyzed as two separate explanatory variables in order to account for directionality. Distance from the hospital, latitude, and longitude were first each analyzed separately with respect to IgG seropositivity by logistic regression.

Distance from the hospital, distance from water, and latitude proved not to be a significant factor in predicting IgG seropositivity (p = 0.98, 0.22, 0.62, respectively). Longitude

was significant (p = 0.004), indicating that movement from east to west (i.e., a decrease in longitude) significantly increases IgG seroprevalence (Figures 5-7). A logistic regression of IgG status on longitude yields the following formula:

$$logit(p) = -0.41 - 2.29(long + 79.5).$$

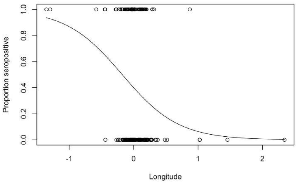

Figure 5: *T. gondii* IgG seropositivity rates among pregnant women by longitude. Longitude 0 represents the longitude of Hospital Santo Tomás. A one unit increase in this longitude represents a one unit increase in the actual decimal degree longitude unit system.

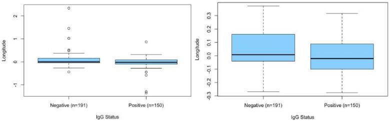

Figure 6: Boxplots of longitude versus IgG status, including outliers (left panel) and excluding outliers (right panel). Outliers are defined as points outside 1.5 times the interquartile range above the upper quartile and below the lower quartile. The black bar represents the median, the blue box represents the interquartile range, and the whiskers represent the minimum and maximum values for each group (excluding outliers). Longitude 0 represents the longitude of Hospital Santo Tomás.

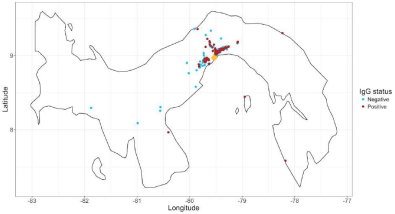

Figure 7: IgG status distribution by latitude and longitude. The yellow diamond at (-79.53, 8.97) marks the coordinates of Hospital Santo Tomás. The data points for Panama's coastal borders were drawn from the "world" database. This figure was generated using the ggplot function from the ggplot2 library. Patient coordinates, as well as the coordinates of Hospital Santo Tomás, were added using geom_point.

Animal contact

First, a principal component analysis was conducted in an attempt to reduce the dimensionality of the animal contact data. The analysis determined that of the eight animal contact variables, five principal components were required to explain at least 70% of the variance (Figure 8a). Therefore, this analysis was not useful in reducing the dimensionality of the data.

Combining street animals and pets of each species, contact with dogs proved to be the only significant animal factor at the $\alpha = 0.05$ confidence level (χ^2 test, $p = 0.037$). Contact with dogs increases the proportion of seropositivity (Figure 8b). However, after correcting for multiple testing, this feature is no longer significant. While contact with cats (either on the street or as pets) was not found to be a significant factor as hypothesized (χ^2 test, $p = 0.353$), groups with contact with cats did have higher IgG seropositivity rates (Table 2a). This variable was also significantly associated to contact with dogs (Table 2b, χ^2 test, $p = 2.0 \times 10^{-12}$). Since this

variable had a smaller sample size than the dogs variable, it is possible that cats are also associated with IgG risk, but the small sample size does not provide enough power for a significant result.

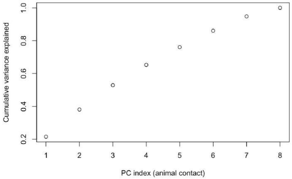

Figure 8a: Cumulative variance explained by each principal component of the animal contact variables.

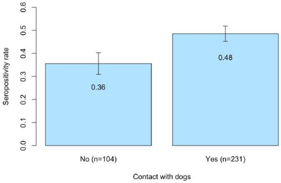

Figure 8b: IgG seropositivity rate based on contact or no contact with dogs (both in the street or as pets). Error bars represent the standard error of the mean.

Contact with cats	IgG negative	IgG positive	Proportion contact with cats
**No**	95	90	0.49
**Yes**	68	81	0.54

Table 2a: Association between contact with cats and IgG seropositivity.

Contact with dogs	No contact with cats	Contact with cats	Proportion contact with cats
**No**	81	23	0.22
**Yes**	82	148	0.64

Table 2b: Association between contact with dogs and cats.

Diet

As with the animal contact data, a principal component analysis was conducted in an attempt to reduce the dimensionality of the diet variables. The values for the ordinal (non-binary) variables were divided by 3 in order to normalize the distance between all variables. The analysis determined that of the eleven diet variables, five principal components were required to explain at least 70% of the variance (Figure 9). Therefore, this analysis was not useful in reducing the dimensionality of the data.

Pairwise χ^2 analyses were conducted between diet variables. The only cluster of three or more variables with significant associations between all pairs within the cluster was regular consumption of pig, regular consumption of seafood, and frequency of raw meat consumption (χ^2 test, pig - seafood p = 7.0×10^{-13} , pig - raw meat p = 0.05, seafood - raw meat p = 0.01). Regular consumption is defined as consumption at least a couple of times per week. A logistic regression of pig on seafood yields an odds ratio of 9.7, and ordinal regressions of raw meat on each of pig and seafood yields odds ratios of 2.2 and 2.4, respectively. An analysis of the three-way interaction between pig, seafood, and raw meat indicates yields a significant result (log-linear model, p = 0.012).

Raw meat, pig, and seafood were combined into a single variable, where patients who regularly consume pig or seafood, or who eat any raw meat at all, were grouped together, and patients who do not regularly consume pig or seafood and do not eat raw meat were grouped together. This variable representing the cluster of raw meat, pig, and seafood is almost significantly correlated with IgG seropositivity, but not quite (χ^2 test, p = 0.09).

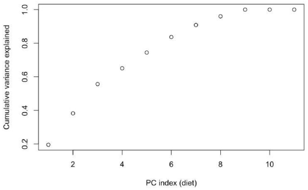

Figure 9: Cumulative variance explained by each principal component of the diet variables.

Hygiene

For each of the two hygiene variables (hand washing and produce washing) the categories for "Never" and "Sometimes" were combined for increased power. A χ^2 analysis found a significant positive association between the two variables (p = 5.7×10^{-7}). However, neither hand washing nor produce washing was found to be significantly associated with IgG seropositivity (χ^2 test, p = 0.41, 0.57, respectively).

Water source

Most patients obtained their water from an aqueduct (plumbing), while a few obtained their water from wells, rivers, and rainwater, grouped together as "Other". Water source was not

found to be a significant factor in determining risk of IgG seropositivity (Fisher's Test, p = 0.56), probably due to the low sample size of subjects in the "Other" category.

Prediction model

All models in this section were evaluated based on the following calculated values and definitions:

Root mean squared error (RMSE) = $\sqrt{\frac{1}{n}\sum_{i=1}^{n}(y_i - \widehat{y}_i)^2}$,

where $y_i = 0, 1$ is the true seropositivity status of individual *i* (seronegative, seropositive, respectively), and $\widehat{y}_i$ is the predicted seropositivity status of individual *i*

Mean error ($\overline{e}$) = $\frac{1}{n}\sum_{i=1}^{n}\left|y_i - \widehat{y}_i\right|$

False positive rate (FPR) = $\frac{\#FP}{\#FP + \#TN}$,

where FP are false positives ($\widehat{y}_i = 1$, $y_i = 0$) and TN are true negatives ($\widehat{y}_i = 0$, $y_i = 0$)

False negative rate (FNR) = $\frac{\#FN}{\#FN + \#TP}$,

where FN are false negatives ($\widehat{y}_i = 0$, $y_i = 1$) and TP are true positives ($\widehat{y}_i = 1$, $y_i = 1$)

Accuracy = $\frac{\#TN + \#TP}{\#FP + \#TN + \#FN + \#TP}$

The single logistic regressions found only two variables, education and longitude, to be significantly and independently correlated with IgG seropositivity. A multiple logistic regression of IgG status against these two variables was run on the 200 patients for whom there is no missing data. Of these 200 patients, 119 are seronegative and 81 are seropositive. The logistic regression yields the following equation, where both variable coefficients are significant (p = 0.001, p = 0.005, respectively): $logit(p) = 202.9 - 0.9e + 2.5long$. This equation was tested on the set of 200 patients, where *p* was rounded to the nearest integer (0 or 1) and compared to the actually seropositivity status of the patient. This model yields an RMSE of 0.46, ME of 0.43, and accuracy of 0.68, as well as a false positive rate of 0.11 and a false negative rate of 0.64.

Subsequently, a Lasso regression was run for more robust variable selection and regularization in a multivariate setting. The Lasso method aims to find β_0, β such that the following Lasso function is minimized:

$$L(\beta) = \frac{1}{N}\sum_{i=1}^{N}\left[y_i\left(\beta_0 + x_i^T\beta\right) - log\left(1 + e^{(\beta_0 + x_i^T\beta)}\right)\right] + \lambda\|\beta\|_1 ,$$

where the first term is the negative binomial log-likelihood of the logistic regression equation, and $\lambda \geq 0$ is the tuning, or penalty, parameter, which determines the extent of shrinkage of the coefficients (Reference 15). If $\lambda = 0$, this is equivalent to an ordinary least squares logistic regression. The aim of the Lasso model is to minimize the number of coefficients, which is useful in the case of multicollinearity, as with the present data.

The Lasso regression was performed using the glmnet function in the glmnet library. This model produces a binary response of either 0 (seronegative) or 1 (seropositive). The Lasso regression was run on the 200 patients for whom there is no missing data.

First, a training set was created by removing one of the patients. This was repeated 200 times for each permutation of possible training sets. Each of these 200 repetitions was run with a range of 1000 equidistant tuning parameters within [10^{-5}, 10]. For each repetition, the λ value that minimized the mean cross-validated error was found using the cv.glmnet function in the glmnet library. A histogram of the 200 λ values demonstrates that they are more or less symmetrically distributed within [0,0.08], with a mean at 0.04 (Figure 11).

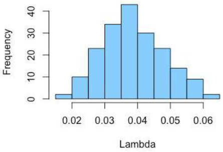

Figure 11: Histogram of λ values that minimize the mean cross-validated error of the Lasso regression for each of the 200 permutations of "leave one out" test sets.

A Lasso regression was then run on the entire dataset of 200 patients for each of the 200 λ values to evaluate the accuracy of each model tuning parameter. The RMSE, ME, and accuracy were assessed for each λ value (Figure 12).

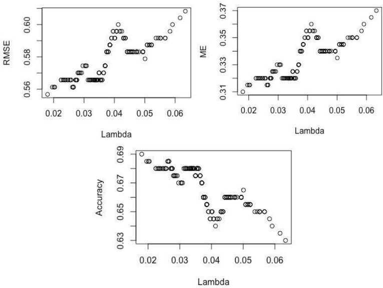

Figure 12: RMSE (top left), ME (top right), and accuracy (bottom) for each of the 200 λ values from the Lasso cross-validation step.

The minimum RMSE and ME, as well as the maximum accuracy, are found at λ = 0.017 . A narrower range of λ values was then selected around 0.017 in order to fine-tune the penalty parameter. The λ were chosen to be 100 equidistant values within [0.01,0.02]. Once again, the Lasso regression was repeated 200 times, once for each patient as an outgroup, on this new range of λ values, and the λ value that minimized the mean cross-validated error for each repetition was found. A histogram of these λ values indicates that the majority lie within [0.019,0.020] (Figure 13).

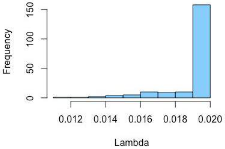

Figure 13: Histogram of λ values around 0.017 that minimize the mean cross-validated error of the Lasso regression for each of the 200 permutations of "leave one out" test sets.

Again, RMSE, ME, and accuracy were assessed for each of these λ values (Figure 14).

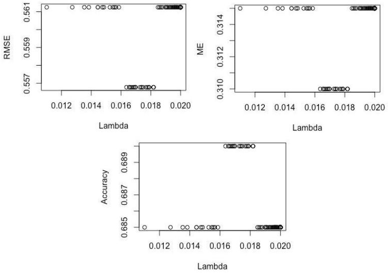

Figure 14: RMSE (top left), ME (top right), and accuracy (bottom) for each of the 100 λ values around 0.017.

The range of λ values that minimize the RMSE and ME, as well as maximize the accuracy, are found within [0.016,0.018], with a mean at 0.017. Therefore, the original optimal λ value of 0.017 was taken to be the optimal fine-tuned λ value.

The model using $\lambda = 0.017$ yields an RMSE of 0.56, ME of 0.31, and accuracy of 0.69. Additionally, the model has an FPR of 0.14 and FNR of 0.56 (Figure 15). The nonzero coefficients produced by this Lasso regression with $\lambda = 0.017$ can be seen in Table 3. As expected, longitude has the strongest influence on IgG status, with a positive association; education is also highly influential, ranking third in terms of influence among the features, with a negative association. Other influential variables are water distance, pet dogs, contact with wild animals, food stands, restaurants, street dogs, raw meat, age, and pet cats. All other coefficients are shrunk to zero.

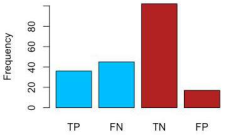

Figure 15: True positive, false negative, true negative, and false positive rates, respectively of the Lasso model with $\lambda = 0.017$. Blue represents seropositive and red represents seronegative. FPR is calculated as FP divided by the sum of the red bars, and FNR is calculated as FN divided by the sum of the blue bars. The sum of frequencies of all 4 bars adds up to 200.

Feature	Coefficient
Intercept	169.1
Longitude	2.1
Water distance	-0.9
Education	-0.8
Pet dogs	0.6
Contact with wild animals	-0.5
Food stands	0.3
Restaurants	0.3
Street dogs	0.2
Raw meat	-0.07
Age	0.004
Pet cats	-0.004

Table 3: Coefficients produced by Lasso regression, $\lambda = 0.017$, in decreasing order of magnitude.

The two models in this section, logistic and Lasso regressions, were compared to a model of random guessing, in which all 200 patients would be predicted to be seronegative, since that is the status of the majority of patients. In this case, the RMSE is 0.64 and ME is 0.41. The accuracy rate of this model is 0.60, since this is the proportion of seronegative individuals in the group. Additionally, FPR is 0 and FNR is 1, since all seropositive patients are found to be seronegative (Table 4).

Model	Logistic regression	Lasso	Random guessing
**RMSE**	*0.46*	0.56	0.64
**ME**	0.43	*0.31*	0.41
**Accuracy**	0.68	*0.69*	0.60
**FPR**	*0.11*	0.14	0
**FNR**	0.64	*0.56*	1

Table 4: Comparison of root mean squared error, mean error, accuracy, false positive rate, and false negative rate of the logistic regression and Lasso models versus random guessing. The “best” value for each metric is italicized and underlined; “best” is defined as minimum for RMSE, ME, FPR, and FNR, and maximum for accuracy.

Discussion

The purpose of this investigation was to determine risk factors for *Toxoplasma gondii* IgG seropositivity among pregnant women in Panama and to develop a model for predicting *T. gondii* IgG seropositivity in this population. Univariate analyses found age, education, and longitude to be significantly associated with seropositivity. However, neither age nor age squared is a significant feature when regressing out education. This is likely due to the strong collinearity between the age variables and education. Thus, age is likely a proxy for education, but not the main effect. Interestingly, education levels are highest at the extreme ends of the age spectrum, while patients around the mean age have a lower mean education level. Older age at childbirth likely reflects a more educated afluent population that defer childbearing for education but the lower age remains unexplained.The results of the longitude analysis are consistent with previous studies that have found significant general trends of increasing seropositivity from the western end to the eastern end of the country in both humans (Wang, Pandey, & Moreira, unpublished) and domestic dogs and cats (Rengifo-Herrera *et al.*, 2017).

Through the Lasso model with $\lambda = 0.017$, we can predict IgG seropositivity status with 69% accuracy, as opposed to 60% accuracy with random guessing and 68% with the logistic regression. However, because the number of seronegative women outweighs the number of seropositive, both the Lasso and logistic models yield low false positive rates (0.14, 0.11, respectively) but high false negative rates (0.56, 0.64, respectively). That is, if a patient is

seronegative, the Lasso model will likely detect this status accurately, but the model is not as accurate in detecting seropositivity. Overall, the Lasso model is more accurate than logistic regression in determining IgG status (high accuracy) and is almost as specific in its detection of patient seronegativity (low FPR). False negatives are the main source of error with both models, since the majority of patients are IgG seronegative. In that respect, the Lasso model performs the best, with the lowest FNR. However, this model still needs to be fine-tuned for increased sensitivity to seropositivity.

One surprising finding is that the Lasso model yielded a negative effect of raw meat, wild animal contact, and pet cats on IgG seropositivity. This is contrary to previous studies which have found raw meat consumption, wild animal contact, and exposure to cats to contribute to IgG seropositivity (Hill & Dubey 2002, Etheredge *et al.*, 2004). This result may be due to confounding between these variables and other features, either collected in this study or not. For example, the Lasso regression found exposure to dogs to have an influential positive effect on IgG seropositivity, and the univariate analyses found contact with cats and contact with dogs to be significantly negatively correlated. This might explain why the Lasso regression found pet cats to have a negative influence on IgG seropositivity.

Interestingly, water distance was found to be more influential than education using this Lasso regression, despite the fact that water distance was not found to be significantly associated with IgG status in the univariate analysis. Nevertheless, this result of negative association between water distance and IgG seropositivity is consistent with previous studies that have found proximity to water to be a risk factor for *T. gondii* exposure (Wang, Pandey, & Moreira, unpublished). Age was also found to be influential, although it exerts a relatively small effect, probably due to its confounding with the effects of education. Nonetheless, this nonzero coefficient of age suggests that it may play a small role independent of education level. Pet dogs were also found to exert an influential effect on IgG status, even though multiple testing corrections in the univariate analyses found pet dogs not to be significantly correlated with IgG status.

The remaining nonzero coefficients, those for food stands and restaurants, are positive, indicating that eating out increases chances of IgG seropositivity. This result is consistent with

those of previous studies that have found poor food hygiene to be associated with *T. gondii* exposure (Hill & Dubey 2002).

This analysis was limited in that there was missing data, a problem which could be solved by imputation. Furthermore, there are other informative variable that could have been collected, such as income level or country of origin, that may have confounded the results of the study. Additionally, the Lasso model only produced coefficients, not p-values, so while some features were found to have a large influence on IgG status, the significance of this effect is unknown.

Future studies may address these limitations and explore some of these questions deeper. For instance, this study treated IgG status as a binary response of either seropositive or seronegative, which was based on a cutoff value of the magnitude of antibody titer. Future studies could look at antibody titer as a continuous variable rather than a binary response in order to see if strength of seropositivity is associated with any of the risk factors assessed in this study.

References

1. Araujo, F. G., and J. S. Remington. "Toxoplasmosis in immunocompromised patients." *European Journal of Clinical Microbiology & Infectious Diseases* 6.1 (1987): 1-2.
2. Barrera, Ana M., et al. "Toxoplasmosis adquirida durante el embarazo, en el Instituto Materno Infantil en Bogotá." *Revista de Salud Pública* 4.3 (2002): 286-293.
3. Becker, Richard A., and Allan R. Wilks. "Constructing a geographical database." *AT\&T Bell Laboratories Statistics Research Report [95.2]* (1995).
4. Castro, A. T., A. Congora, and M. E. Gonzalez. "Toxoplasma gondii antibody seroprevalence in pregnant women from Villavicencio, Colombia." *Orinoquia* 12.1 (2008): 91-100.
5. Etheredge GD, Michael G, Muehlenbein MP, Frenkel JK. The roles of cats and dogs in the transmission of *Toxoplasma* infection in Kuna and Embera children in eastern Panama. Rev Panam Salud Publica. 2004;16(3):176–86.
6. Hall, Susan M. "Congenital toxoplasmosis." *BMJ: British Medical Journal* 305.6848 (1992): 291.
7. Hill, D., and J. P. Dubey. "Toxoplasma gondii: transmission, diagnosis and prevention." *Clinical microbiology and infection* 8.10 (2002): 634-640.
8. *Index of /Workshops/Rgis/Data*, www.richardcondit.org/workshops/Rgis/data/.
9. Joynson, D. H. M., and E. C. Guy. "Laboratory diagnosis of toxoplasma infection." *Toxoplasmosis. A Comprehensive Clinical Guide*. Cambridge University Press, Cambridge, 2001. 296-318.
10. Luft, Benjamin J., and Richard Hafner. "Toxoplasmic encephalitis." *Aids* 4.6 (1990): 593-596.

11. Pappas, Georgios, Nikos Roussos, and Matthew E. Falagas. "Toxoplasmosis snapshots: global status of Toxoplasma gondii seroprevalence and implications for pregnancy and congenital toxoplasmosis." *International journal for parasitology* 39.12 (2009): 1385-1394.
12. R Core Team (2015). R: A language and environment for statistical computing. R Foundation for Statistical Computing, Vienna, Austria. URL http://www.R-project.org/.
13. Rengifo-Herrera, Claudia, et al. "Seroprevalence of Toxoplasma gondii in domestic pets from metropolitan regions of Panama." *Parasite* 24 (2017).
14. Rosso, Fernando, et al. "Prevalence of infection with *Toxoplasma gondii* among pregnant women in Cali, Colombia, South America." *The American journal of tropical medicine and hygiene* 78.3 (2008): 504-508.
15. *Stanford Statistics Technical Report Glmnet Vignette* https://web.stanford.edu/~hastie/glmnet/glmnet_alpha.html.
16. Tenter, Astrid M., Anja R. Heckeroth, and Louis M. Weiss. "Toxoplasma gondii: from animals to humans." *International journal for parasitology* 30.12-13 (2000): 1217-1258.
17. Wilson, Christopher B., et al. "Development of adverse sequelae in children born with subclinical congenital Toxoplasma infection." *Pediatrics* 66.5 (1980): 767-774.
18. Zapata, M., L. Reyes, and I. Holst. "Decreased prevalence of Toxoplasma gondii antibodies in adults form the Central Valley in Costa Rica." *Parasitol Latinoam* 60 (2005): 32-7.

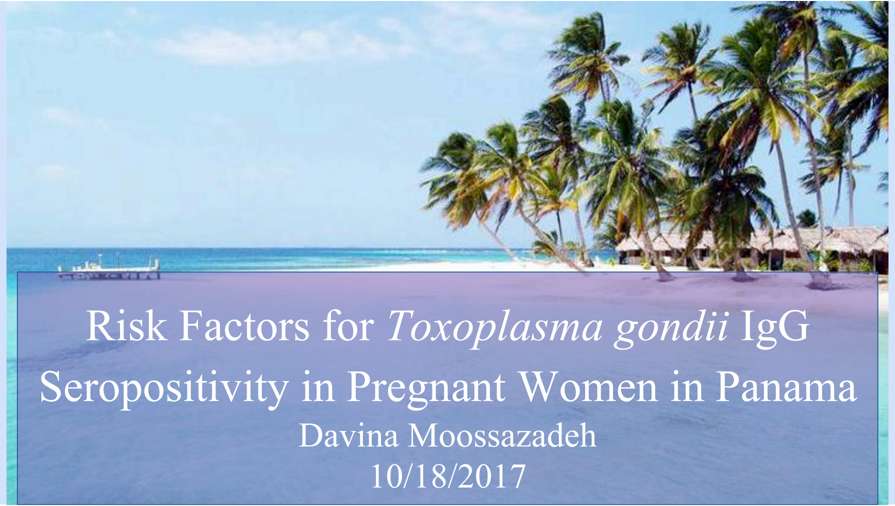
Risk Factors for *Toxoplasma gondii* IgG
Seropositivity in Pregnant Women in Panama
Davina Moossazadeh
10/18/2017

Toxoplasmosis

- Protozoan parasite *Toxoplasma gondii*
  - Definitive host is the cat
- May be transmitted to the fetus when a woman acquires it for the first time during pregnancy
  - Ocular
  - Neurologic
  - Miscarriage

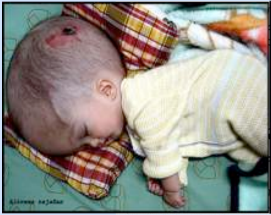

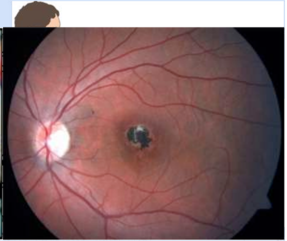

Wilson *et al.*, 1980

IgG Seropositivity

- Infection before conception protects the fetus
- IgG persists for life
  - Seropositivity = prior infection
- IgG test: screen out pregnant women with minimal risk

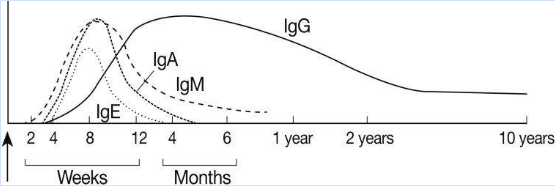

Joynson & Guy, 2001

IgG seroprevalence in Panama

- No published studies
- 50% of women of childbearing age IgG+ (Sáez Llorens *et al.*, 2014, unpublished)
- Neighboring countries (Georgios *et al.*, 2009)
  - Colombia: 43.1-66.7%
  - Costa Rica: 49.2-60.8%
- Previously identified risk factors (Etheredge *et al.*, 2004)
  - Soil floors
  - Dogs and cats

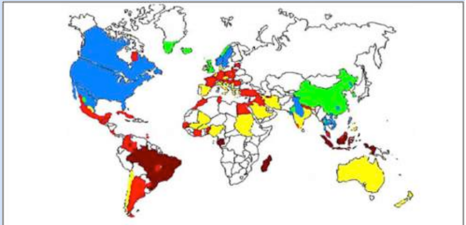

Global status of *Toxoplasma gondii* seroprevalence. Dark red equals prevalence above 60%, light red equals 40–60%, yellow 20–40%, blue 10–20% and green equals prevalence <10%. White equals absence of data. Adapted from [52].

Research Objective

To assess risk factors for *T. gondii* IgG seropositivity among pregnant women in Panama

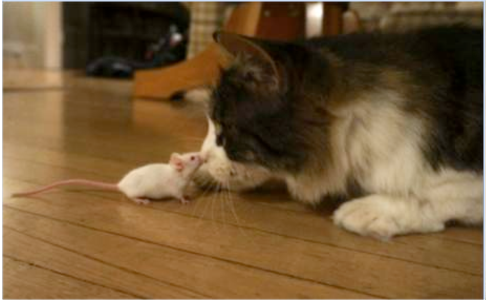

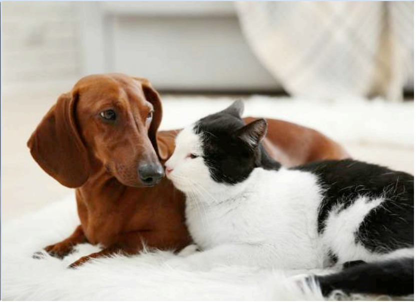

Methods

- Tested 343 pregnant women for *T. gondii* seropositivity at Hospital Santo Tomás
- Assessed pregnant women for risk factors via questionnaire
  - Demographics
  - Contact with animals
  - Food hygiene
  - Water source
- Pearson’s chi-squared, Fisher’s exact tests in R

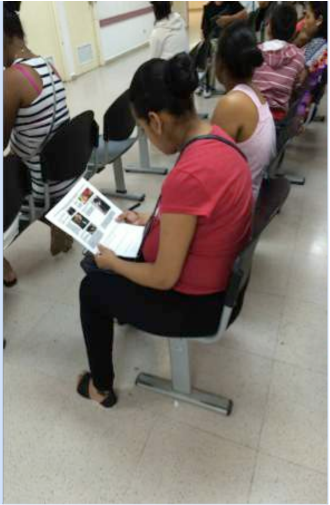

2016: Geography

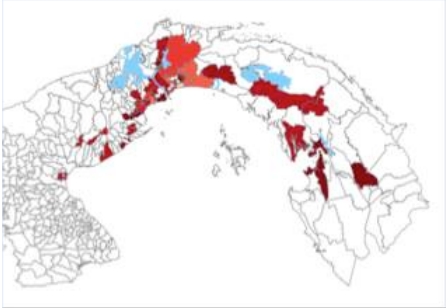

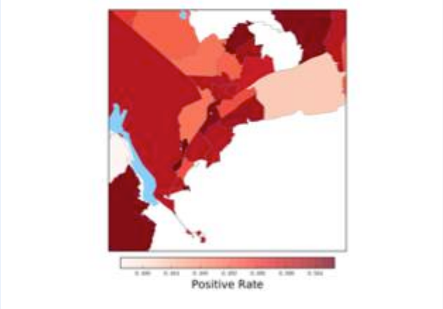

Curundú 24x, Pueblo Nuevo 10x increase

West to east increase

Wang, Pandey, & Moreira 2016

2016: Corregimiento Curundú

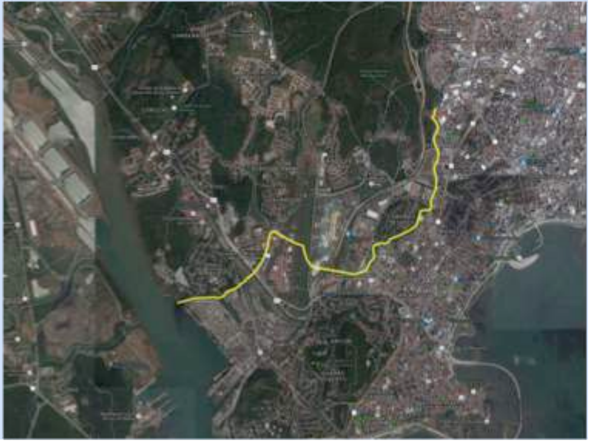

IgG positive

IgG Negative

Not screened

2017: Geography

- West to east
  - Humans ($p = 0.09$)
  - Domestic animals (Rengifo *et al.*, 2017)

Panamá Oeste: 35.0%

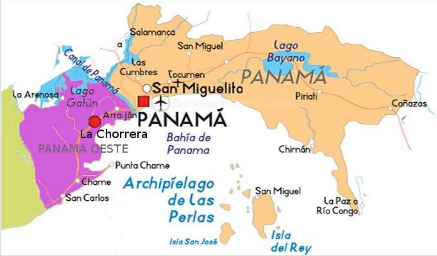

Panamá: 46.7%

(Lykins & Wroblewski, 2017)

Significantly High Seroprevalence in Curundú* (*p = 0.025)

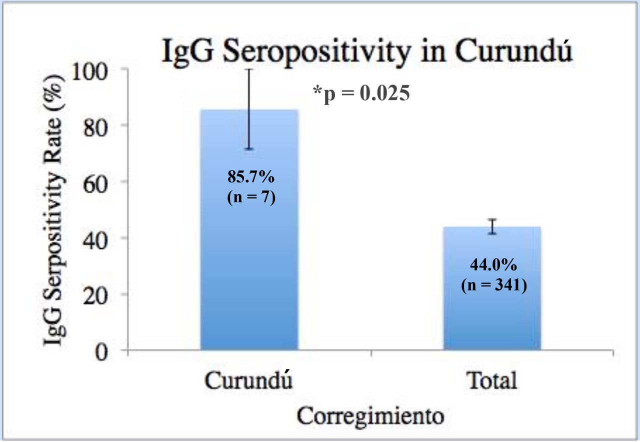

Significant Associations with Maternal Age* (*$p < 0.05$)

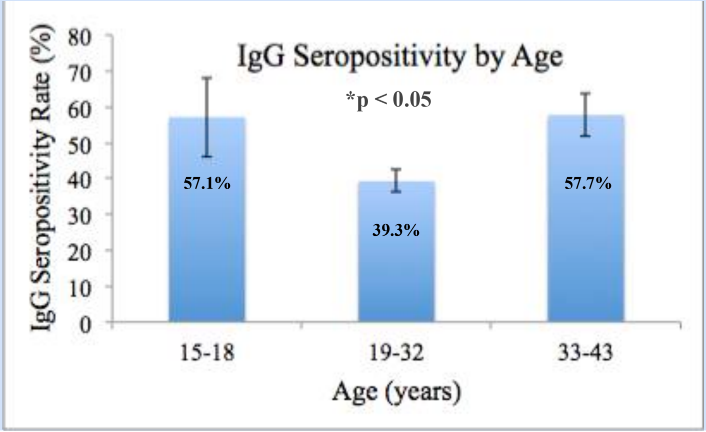

Significant Associations with Education (*p < 0.05)

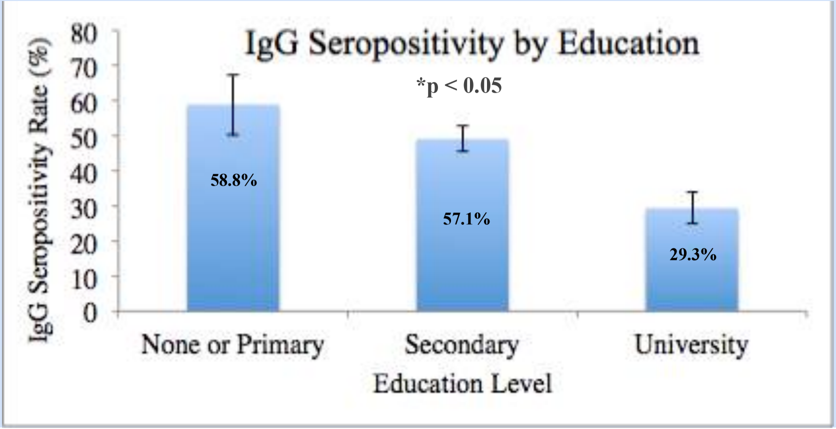

Insignificant Factors ($p \geq 0.05$)

- Pets
- Contact with wild animals
- Presence of street animals
- Street food
- Origin of food consumed
- Washing hands
- Washing fruits and vegetables

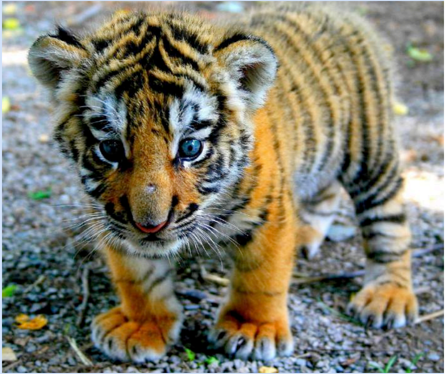

Limitations

- Self-reported data
- Multiple researchers obtaining data
- No centralized electronic medical record keeper
- No correcting of education for age

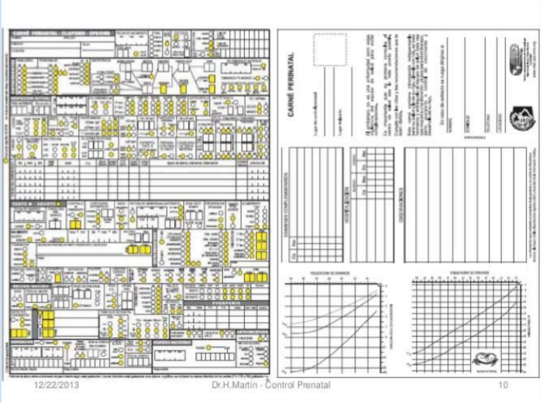

Conclusions

- Concordance of young age and education with IgG seropositivity
  - Lower socioeconomic status may be a risk factor
- <u>However, IgG was found in all age groups and all education groups</u>

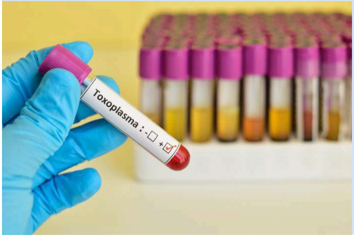

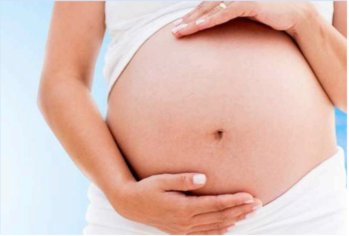

Future Studies

- Addressing absence of other significant risk factors
  - Unidentified risk factors
  - Insufficient sample size
- Water and soil studies
  - Subinoculation of mice
- Latitude and longitude studies
- Point-of-care testing

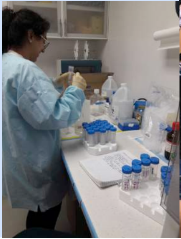

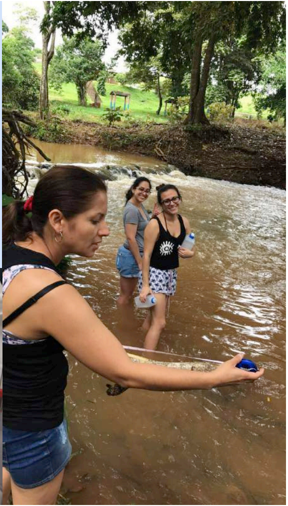

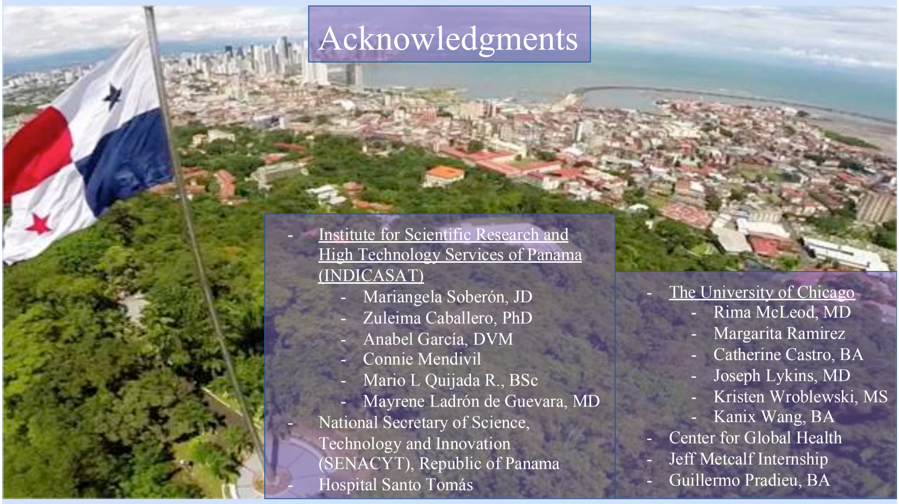

Acknowledgments

- Institute for Scientific Research and High Technology Services of Panama (INDICASAT)
  - Mariangela Soberón, JD
  - Zuleima Caballero, PhD
  - Anabel García, DVM
  - Connie Mendivil
  - Mario L Quijada R., BSc
  - Mayrene Ladrón de Guevara, MD
- National Secretary of Science, Technology and Innovation (SENACYT), Republic of Panama
- Hospital Santo Tomás
- The University of Chicago
  - Rima McLeod, MD
  - Margarita Ramirez
  - Catherine Castro, BA
  - Joseph Lykins, MD
  - Kristen Wroblewski, MS
  - Kanix Wang, BA
- Center for Global Health
- Jeff Metcalf Internship
- Guillermo Pradieu, BA

Many Thanks!

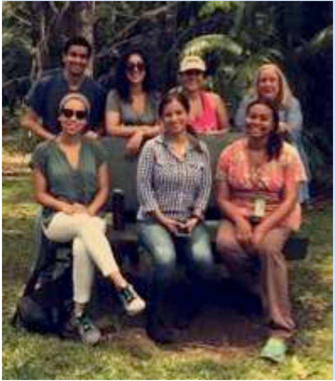

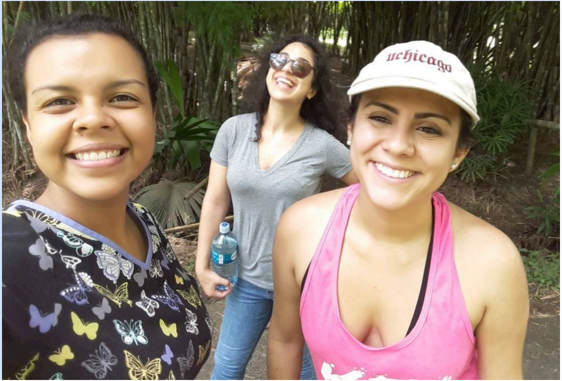

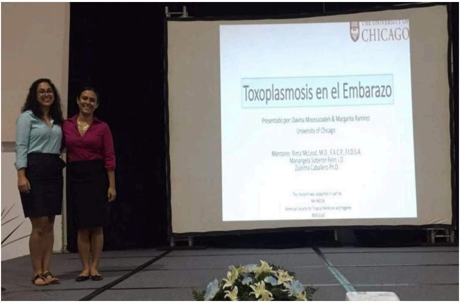

Margarita Ramírez
with Joseph Lykins, MD; Kanix Wang, PhD; Mariángela Soberón Felín; Davina Moossazadeh; Zuleima Caballero, PhD; Ximena Norero, MD; Dora Estripaut, MD; Xavier Sáez Loorens, MD; Rima McLeod, MD; and Instituto de Investigaciones Científicas (INDICASAT) – Panama; Hospital Santo Tomás – Panama; Hospital del Niño – Panama

THE UNIVERSITY OF CHICAGO
Center for Global Health
INDICASAT AIP

An Analysis of Gestational Screening for Toxoplasmosis in Panama:
Non-systematic screening & its Effect on Maternal-Fetal Health

M.Ramirez[1], J. Lykins[2], K. Wroblewski[2], K. Wang[2], M. Soberón Felin[3], D. Moossazadeh[1], Z. Caballero[4], R. McLeod[2]; [1]Chicago, IL/US, [2]Chicago/US, [3]Panama/PA, [4]Panama City/PA

Background

Toxoplasmosis is a disease caused by the parasite T. gondii. Congenital infection results when a previously seronegative pregnant woman becomes acutely infected, and transmits the infection to her baby. An acute infection is typically asymptomatic and may go unnoticed. Because of this, screening is vital for diagnosis. In Panama, two screenings during pregnancy are mandated; however, a retrospective chart review conducted by the Center for Global Health in 2016 found that 70% of women were never screened. Moreover, no studies exist looking at the clinical manifestations of the congenital disease in Panama.

Objectives

Overall objective: characterization of acute & congenital Toxoplasma gondii infection in Panama.

Specifically, characterize:

I. Differences between screened and unscreened women
II. Clinical manifestations of congenitally infected babies
III. Acutely infected mothers

Methods

I. 343 pregnant women were sampled throughout the course of 10 weeks at the Maternity Hall of Hospital Santo Tomas, the largest public hospital in Panama

- All women were given an educational toxoplasmosis pamphlet *(figure 1)*
- Following this, a questionnaire (figure 2) was administered to record:
  - Demographics
  - Exposure to known risk factors
  - Prior serology results
- 5 mL blood samples drawn to measure T.gondii specific IgG and IgM antibodies.

II. Retrospective chart review of all congenital toxoplasmosis cases in 2016-2017 at Hospital del Niño

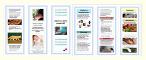

Figure 1. (left) Pamphlet on toxoplasmosis distributed to pregnant women

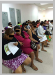

Results

I. Screened vs Unscreened Women

- 80.2 % had yet to be screened, despite being far along in their pregnancies.
- A higher proportion of women from urban settings were previously screened; however, the difference was not significant enough to make a generalization.
- There exists a trend towards an association between education and screening (p = 0.07)

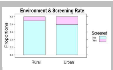

Figure 4 Residential Environment & its effect on screening rate

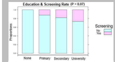

Figure 5 Education & its effect on screening rate

II. IgM+ Women

- 44.0% of women found to be chronically infected (IgG+)
- 2.0% found to be acutely infected (IgM+), in contrast to 5.5% in the summer of 2016
- IgM+ women span the education level spectrum
- For both summers, the average age of IgM+ women was lower than the average age of their cohort
- IgM+ women found in both rural and urban locations of

	IgM+	Cohort
Avg Age Summer 2017 (yrs)	25.57 ± 9.03	27.28 ± 6.23
Avg Age Summer 2016 (yrs)	24.43 ± 7.24	26.59 ± 7.15

Figure 4 (left) *Average age of acutely infected women vs average age of their cohort*
Figure 5 (below) Ages of acutely infected women

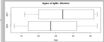

III. Clinical Manifestations of Congenitally Infected Children

- Of the 6 children diagnosed, the only child that was not severely affected received prenatal treatment (Figure 6)

Patient	*Gestational Age at Birth*	*Prenatal Treatment*	*Clinical Manifestations at Birth*	*Laboratory Testing*	*Treatment*	*Mortality*
1	38	Received spiramycin at 14 weeks	Asymptomatic	IgG Positive IgM Negative	Clindamycin, Pyrimethamine	No
2	41	None	Hydrocephalus, microcephaly; ≥ 3 intracranial calcifications; jaundice	IgE Positive	Patient lost to follow-up, but on folic acid, pyrimethamine, & clindamycin	Unknown
3	37	None	r/o sepsis, chorioretinitis (involving the macula) dx at 1 month; resolved with 8 months of tx; cardiopathy	IgE Positive	Clindamycin, Pyrimethamine	No
4	37	None	Hydrocephalus; < 3 intracranial calcifications; thin cortex; chorioretinitis (involving the macula); jaundice	IgE Positive	Clindamycin, Pyrimethamine	No
5	37	None	Microcephaly; diabetes insipidus; visual impairment with retinal lesion and R-sided intraocular hemorrhage; ventriculomegaly; expired on day 17	IgE Positive	Clindamycin, Pyrimethamine	Yes
6	37	None	r/o sepsis; microcephaly; diabetes insipidus; symptomatic epilepsy with seizure activity near birth; hypothyroidism and rhinopharyngitis	IgE Positive	Ampicillin, Cefotaxime, Clindamycin, Pyrimethamine (often not available); Multiple hospitalizations	No

Figure 6 Summary of Clinical Manifestations in Hospital del Niño

Discussion

Non-systematic screening remains a problem, as it delays accurate diagnosis and postpones and inhibits appropriate treatment.

- Infection risk is present irrespective of socioeconomic status, age, and residential environment.
- Our studies have not identified a set formula for indicating infection and the source of infection remains unrecognized in Panama.
- This emphasizes the need for systematic screening to track seroconversion and propels already initiated research to look for the parasite in water and soil, and genotype parasites found in the placenta of infected mothers and Panamanian wildlife.
- Given that education level was the only nearly significant indicator of whether a woman will complete the mandated screening, education initiatives should continue. This education component strives to bring awareness at all levels, from pregnant women to medical professionals.

Acknowledgements

I would like to thank Dr. Rima McLeod and all the members and donors at the Center for Global Health, along with the staff at INDICAST-AIP, Mariangela Soberón, and the doctors at Hospital Santo Tomas and Hospital del Niño, for making this project possible

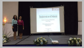

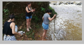

University of Chicago Center for Global Health
Summer Research Fellowship Written Report
October 27th 2017

An Exploration of Gestational Screening for Toxoplasmosis in Panama: Non-systematic Screening & its Effect on Maternal-Fetal Health

MARGARITA RAMIREZ
University of Chicago

Abstract

The objective of this study was to determine the differences between screened and unscreened women in Panama, along with the clinical manifestations of congenitally infected babies, and obtain a descriptive characterization of acutely infected mothers. To do so, surveys and blood samples to measure *T.gondii* specific IgG and IgM antibodies were taken over the course of ten weeks from 343 pregnant women in Hospital Santo Tomas. Moreover, the medical charts of 6 congenitally infected babies at Hospital del Niño were reviewed. 80.2 % of the women in the study had yet to be screened, despite being far along in their pregnancies. When analyzed through chi-squared analysis, there was a trend towards an association between education and screening ($p = 0.07$). Additionally,2.0% were IgM+, with nearly 42.7% of IgM+ women being 20 or younger. These IgM+ women span the education level spectrum, indicating that infection risk is present irrespective of socioeconomic status. Of the 6 children diagnosed at Hospital del Niño in 2016-2017, all but one were severely affected. These results point to problems with non-systematic screening, as it delays accurate diagnosis and appropriate treatment, leading to severely ill children, or children who are initially asymptomatic, but eventually develop symptoms. More importantly, these results provide important data for the Panamanian Ministry of Health about this problem, and thereby an opportunity to intervene and remedy it.

Introduction

Toxoplasmosis refers to the disease caused by the protozoan parasite Toxoplasma gondii, found in a third of the world's population[1,2]. Cats are the definitive hosts for this parasite, and the oocysts shed by cats are a major source of transmission as humans can inadvertently ingest them via contaminated water, undercooked meats and shellfish, and improperly washed produce[1]. Once infected with *T. gondii*, most immunocompetent humans will experience little to no symptoms, which is particularly dangerous when a previously unexposed pregnant women acquires the infection during gestation and unknowingly transmits *T. gondii* to her fetus. While infection in immunocompetent hosts is generally asymptomatic, the clinical manifestations of congenital toxoplasmosis can be severe, although this largely depends on when during gestation the infection was acquired. If the parasite is transmitted early in gestation, severe damage to the retina and central nervous system may occur, including blindness, encephalitis, and microcephaly. Moreover, even if a child is initially asymptomatic, untreated congenital infections are likely to result in chorioretinal lesions and visual impairment by adulthood[3,4].

Diagnosis for toxoplasmosis can be realized through taking blood samples to measure *T. gondii* specific IgG and IgM antibodies. *Figure 1* models the typical IgM and IgG antibody curve from the onset of infection, although this curve

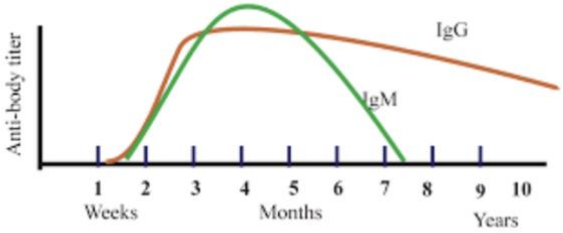

Figure 1 IgM & IgG Curve

may vary by individual. The model shows that when someone is infected, IgM antibodies will rise and fall shortly after, whereas IgG antibodies remain throughout life. Thus, IgG seropositivity is associated with chronic infection while IgM seropositivity is associated with

acute infection. If the patient in question is pregnant, it is important to conduct an additional avidity test to establish the time of acquisition as this data essential for determining the appropriate treatment. For example, if the test suggests that infection occurred earlier than 14-18 weeks of gestation and all ultrasounds are normal, Spiramycin can be administered to block transmission of the parasite. In the case that the ultrasound is not normal, or diagnosis is made after 18 weeks, sulfadiazine, pyrimethamine, and folinic acid are given [4].

Studies have shown that this treatment reduces the incidence and severity of congenital infection manifestations[5]. Thus, several countries with formerly high rates of toxoplasmosis, such as France and Belgium, have developed programs for regular, systematic serodiagnosis and treatment of primary infection during gestation and as a result, have seen a decrease in severe clinical manifestations in children. However, this is not the case for other countries, such as Panama, which has one of the highest rates of toxoplasmosis in the world. Despite needing regular screening to promptly catch seroconversion more than most countries in the world, Panama only requires two screenings for toxoplasmosis during pregnancy, a practice that began with the passing of Executive Order 1617 in October of 2014 [6,7]. In addition to this not being enough to catch seroconversion, a retrospective medical chart review done by the 2016 Panama Global Health Cohort found that 70% of pregnant women were never even screened twice as mandated. Furthermore, after a literature review, the study team found that no studies exist looking at the clinical manifestations and risk factors and demographics associated with acute infection in Panama. Given this information, this investigation had three specific objectives:

1. The first objective was to obtain a descriptive characterization of the demographics of acutely infected mothers and their exposure to known risk factors in a public hospital in Panama City.

2. The second objective was to characterize differences in demographics between screened and unscreened women in this same population.
3. Finally, the third objective was to characterize the clinical manifestations of congenitally infected babies in a public children's hospital in Panama City.

Methods

To carry out these objectives, surveys were completed and blood samples to measure T.gondii specific IgG and IgM antibodies were taken over the course of ten weeks from 343 pregnant women in Hospital Santo Tomas, Panama's largest public hospital, located in Panama City. Moreover, the medical charts of 6 congenitally infected babies were reviewed at Hospital del Niño, Hospital Santo Tomas's sister public pediatric hospital.

I. Testing & Surveys at Hospital Tomas

Our participant population was a convenience sample consisting of 343 pregnant women in the maternity hall of Hospital Santo Tomas. Upon entering the maternity hall, pregnant women were given an educational pamphlet about toxoplasmosis (*figure 2*), along with information about the study. If a pregnant woman agreed to participate, a questionnaire (*figure 3*) was administered to collect information regarding her demographics (address, hometown size, education level, and age) and exposure to known risk factors (contact with animals, water source, and food hygiene and habits), followed by collection of a 5 mL blood sample, conducted by a volunteer doctor, that was then sent to the hospital laboratory to measure T.gondii specific IgG and IgM antibodies. No avidity tests were conducted due to their absence in Panama. Moreover, patient prenatal control cards were requested from patients to record information about prior toxoplasmosis screening results.

After this data was collected and serological test results were acquired, data was analyzed through Pearson's Chi-squared and Fisher's Exact Tests in R. Moreover, patient addresses were used to determine longitude and latitude coordinates using Google Maps, to then create an acute infection incidence map and identify *corregimientos*, or townships, with lower screening rates.

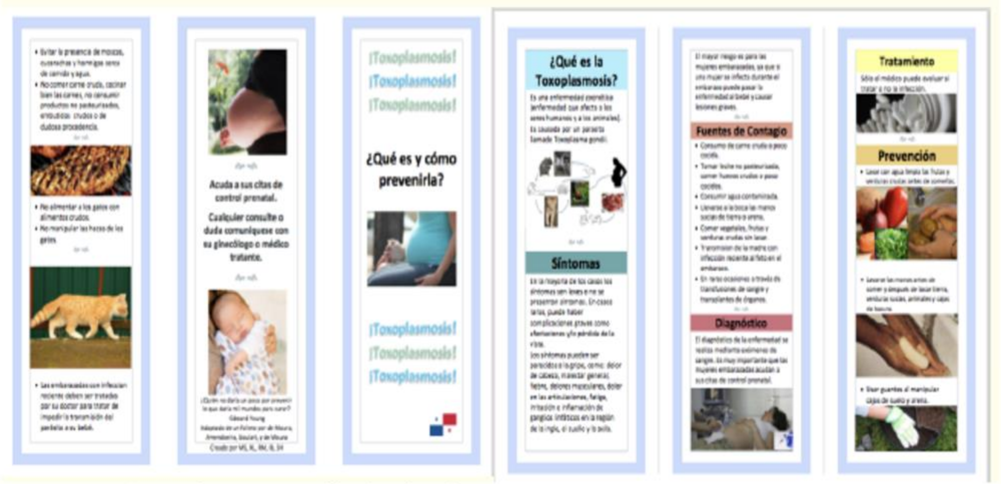

Figure 2 Toxoplasmosis pamphlet distributed to pregnant women

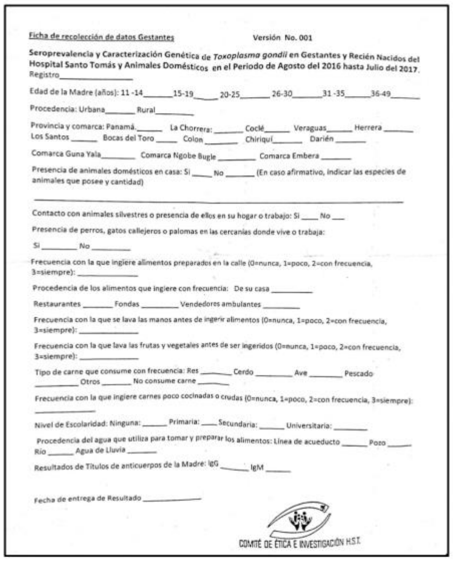

Ficha de recolección de datos Gestantes Versión No. 001

Seroprevalencia y Caracterización Genética de *Toxoplasma gondii* en Gestantes y Recién Nacidos del Hospital Santo Tomás y Animales Domésticos en el Periodo de Agosto del 2016 hasta Julio del 2017.

Registro________________

Edad de la Madre (años): 11 -14______15-19____ 20-25______ 26-30______31 -35______36-49______

Procedencia: Urbana______ Rural________

Provincia y comarca: Panamá.______ La Chorrera: ______ Coclé______ Veraguas______ Herrera ______ Los Santos ______ Bocas del Toro _____ Colon ______ Chiriquí______ Darién ______

Comarca Guna Yala________ Comarca Ngobe Bugle ________ Comarca Embera ______

Presencia de animales domésticos en casa: Si ____ No ______ (En caso afirmativo, indicar las especies de animales que posee y cantidad)

____

Contacto con animales silvestres o presencia de ellos en su hogar o trabajo: Si ____ No ___

Presencia de perros, gatos callejeros o palomas en las cercanías donde vive o trabaja:

Si ________ No _________

Frecuencia con la que ingiere alimentos preparados en la calle (0=nunca, 1=poco, 2=con frecuencia, 3=siempre): ______________

Procedencia de los alimentos que ingiere con frecuencia: De su casa _________

Restaurantes _______ Fondas ________ Vendedores ambulantes ________

Frecuencia con la que se lava las manos antes de ingerir alimentos (0=nunca, 1=poco, 2=con frecuencia, 3=siempre): ______________

Frecuencia con la que lava las frutas y vegetales antes de ser ingeridos (0=nunca, 1=poco, 2=con frecuencia, 3=siempre): ______________

Tipo de carne que consume con frecuencia: Res _______ Cerdo ________ Ave _______ Pescado _________ Otros ______ No consume carne ______

Frecuencia con la que ingiere carnes poco cocinadas o crudas (0=nunca, 1=poco, 2=con frecuencia, 3=siempre): _____________

Nivel de Escolaridad: Ninguna: _____ Primaria: ____ Secundaria: ______ Universitaria: ________

Procedencia del agua que utiliza para tomar y preparar los alimentos: Línea de acueducto ______ Pozo ______ Río ______ Agua de Lluvia _______

Resultados de Títulos de anticuerpos de la Madre: IgG ______ IgM _____

Fecha de entrega de Resultado ____________

COMITÉ DE ÉTICA E INVESTIGACIÓN H.S.T.

Figure 3 Questionnaire collecting patient demographics and exposure risks

II.Chart Reviews at Hospital del Niño

With the permission of the Hospital del Niño bioethics committee, a retrospective chart review of all congenital toxoplasmosis cases in 2016-2017 at Hospital del Niño was performed with the help of Dr. Ximena Norero. For all six cases, prenatal treatment, postnatal treatment, gestational age at birth, clinical manifestations, and laboratory tests were anonymously collected.

Results

<u>Descriptive Characterization of IgM+ women</u>

To begin with, seven women were found to be acutely infected (2.0%) in our study. Furthermore, only two of the acutely infected women had been previously screened for toxoplasmosis and both were only screened once prior to their participation in this study, despite all seven women being at 35 weeks gestation or later.

I. Location

As seen in *figure 4*, acutely infected women were mostly found in the urban center of Panama City. However, infected women in more rural areas were also found.

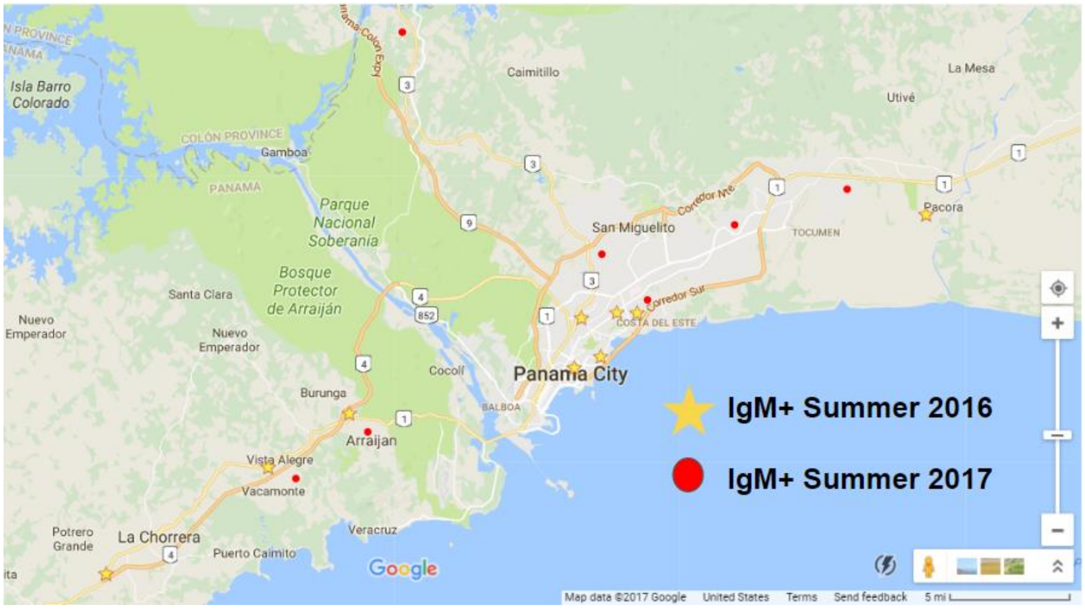

Figure 4 Map of acutely infected women found by the GHC interns in 2016 (marked by stars) and acutely infected women found in 2017

II. Age:

Moreover, this study found that the average age of acutely infected women (25.57 ± 9.03) is lower than the average of their cohort (27.28 ± 6.23). However, acutely infected women of all ages were found, as seen in *figure 5*.

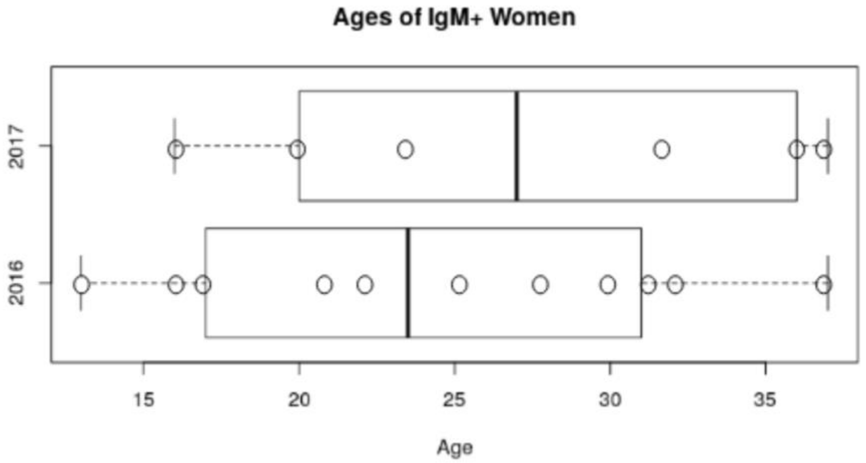

Figure 5 Ages of IgM+ Women in the Summer 2016 Global Health Scholars Study & the Summer 2017 Global Health Scholars Study

III. Education

Lastly, acutely infected women were found to span the education level spectrum - three were university educated, one completed secondary school, and three only completed primary school.

<u>Differences in demographics between screened and unscreened women</u>

I. Education

When analyzed through chi-squared analysis, there was a trend towards an association between education and screening (p=0.07), as seen in *figure 6*, with higher education being associated with a higher screening rate.

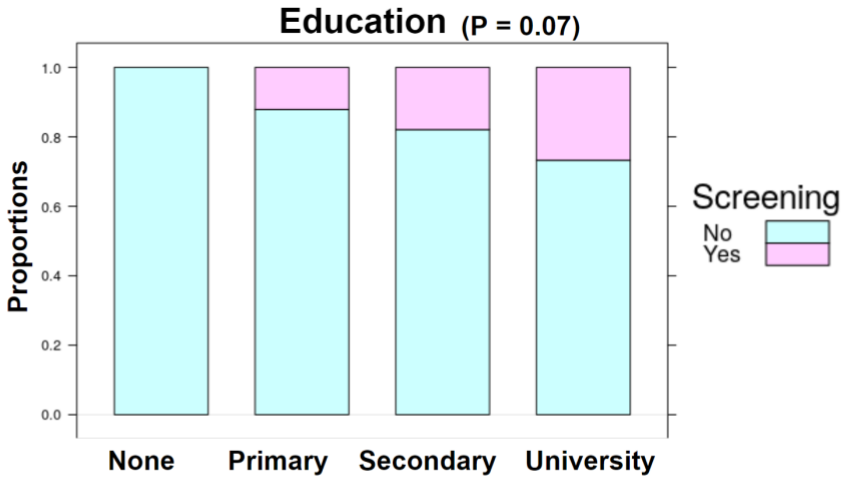

Figure 6 Education levels of previously screened women vs women who had not been screened

II. Location

This year, the township of Belisario Frias, an urban township located in Panama City, had the lowest screening rate. In contrast, a higher proportion of women from urban settings had been previously screened; however, this difference was not significant enough to make a generalization.

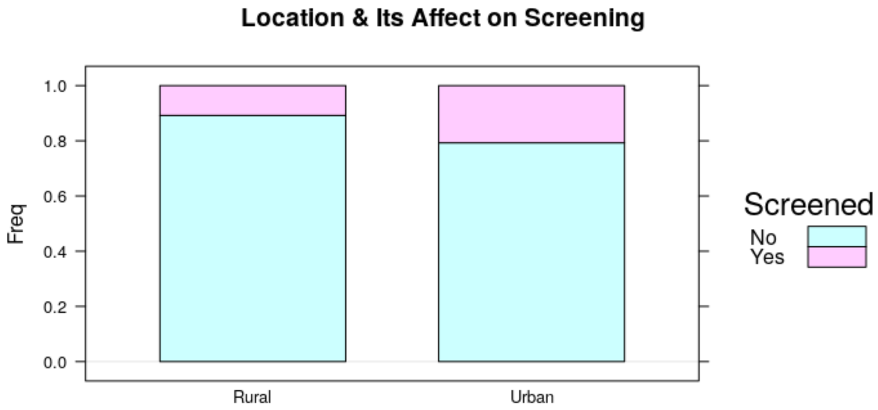

Figure 7 Location & Screening

<u>Clinical Manifestations of Congenitally Infection Children</u>

Table 1 provides a summary of the information from the medical files of congenitally infected children. Of note, only one mother was screened during gestation and treated with

spiramycin. This mother's child was the only child who was asymptomatic, while the rest of the children were found to have a range of clinical manifestations including severe manifestations such as hydrocephalus, microcephaly, diabetes insipidus, chorioretinitis involving the macula, and mild to moderate clinical manifestations such as jaundice, and less than 3 cerebral calcifications.

Patient	***Gestational Age at Birth***	***Prenatal Treatment***	***Clinical Manifestations at Birth***	***Laboratory Testing***	***Treatment***	***Mortality***
1	38	Received spiramycin at 14 weeks	Asymptomatic	IgG Positive IgM Negative	Clindamycin, Pyrimethamine	No
2	41	None	Hydrocephalus, microcephaly, >= 3 intracranial calcifications; jaundice	IgE Positive	VP shunt 12/1/17; Ventricular puncture 12/13/16; No records found after 04/07/2017- patient lost to follow-up; at this time, patient on folic acid, pyrimethamine, and clindamycin	Unknown
3	37	None	Sepsis, chorioretinitis (involving the macula) dx at 1 month; resolved with 8 months of tx; cardiopathy	IgE Positive	Treated with clindamycin and pyrimethamine; external consultant states patient still following this regimen	No

4	37	None	Hydrocephalus; < 3 intracranial calcifications; thin cortex; chorioretinitis (involving the macula); jaundice	IgE Positive	Clindamycin, Pyrimethamine	No
5	37	None	Microcephaly; diabetes insipidus; visual impairment with retinal lesion and R-sided intraocular hemorrhage; ventriculomegaly; expired on day 17; patient tested positive for HSV as well; testicles undescended; reactive lymph nodes appreciated in right inguinal chain	IgE Positive	Clindamycin, Pyrimethamine	Yes
6	37	None	Sepsis; hypothermia; microcephaly; diabetes insipidus; symptomatic epilepsy with seizure activity near birth; hypothyroidism and rhinopharyngitis	IgE Positive	Ampicillin, Cefotaxime, Clindamycin, Pyrimethamine (often not available); Multiple hospitalizations	No

Table 1 Summary of Clinical Manifestations

Discussion

Overall, this exploratory study was the beginning of a greater effort to build a robust program in Panama for detecting acute toxoplasmosis in pregnant women and treating them for the infection. Through this study we found that 7 of the 343 women who participated in the questionnaires and blood draw at Hospital Santo Tomas were acutely infected (IgM+) and of

these seven women, only 2 had been previously screened for toxoplasmosis. Thus, the rest of these women did not know they were infected and at risk of having a child with neurological or ocular disease. This is not uncommon however, as 80% of the women in our study had yet to be previously screened, despite being at 35 weeks gestation or further along. Moreover, these acutely infected women were mostly found in the urban center of Panama City, although this may also be due to a higher population density and proximity to Hospital Santo Tomas. However, acutely infected women were also found in rural areas, suggesting that this is a health problem affecting both urban and rural populations. Another interesting finding was that the average age of acutely infected women (25.57 ± 9.03) was lower than the average age of their cohort (27.28 ± 6.23), a result that is similar to that of the retrospective chart review done in summer 2016 by the 2016 Global Health Fellows (average age of acutely infected women was 24.43 ± 7.24, whereas the average age of their cohort was 26.59 ± 7.15). Nonetheless, it is important to note that in both years, acutely infected women of all ages were found. Lastly, when looking at the differences between screened and unscreened women in the cohort, education level was the only nearly significant indicator of whether a woman would complete the mandated government screening.

Furthermore, the retrospective chart review of six children treated for congenital toxoplasmosis at Hospital del Niño allowed us to begin to characterize the clinical manifestations of congenitally infected babies in Panama. Through this review we found that only one of the children's mothers were screened during gestation and treated for the infection; therefore, all other patients did not receive any prenatal treatment, presumably leading to the development of severe clinical manifestations such as hydrocephalus, microcephaly, diabetes insipidus, chorioretinitis, and even death. Additionally, the sole asymptomatic child in the cohort was the

only child whose mother was screened for toxoplasmosis and received prenatal treatment with spiramycin, highlighting the importance of screening to deliver prompt treatment.

In conclusion, toxoplasmosis is a problem that affects Panamanian families irrespective of socioeconomic status, place of residence, and age. Moreover, non-systematic screening remains a problem in Panama, as it delays accurate diagnosis and appropriate treatment, leading to severe clinical manifestations, and even death of congenitally infected babies. Given that education level was the only nearly significant indicator of whether a woman completed the mandated screening, more efforts should be placed on behalf of the Panamanian Ministry of Health to educate people about toxoplasmosis. Additionally, the study showed that, even if a woman were to be tested twice during gestation, this would not be enough to assure that she is not acutely infected, emphasizing the importance of mandating regular screening throughout pregnancy.

This study provides important data for the Panamanian Ministry of Health about toxoplasmosis, and thereby an opportunity to intervene and remedy it. Furthermore, the focus that has been given to toxoplasmosis and its impact on child and maternal health, has led to several accomplishments. To begin with, Hospital Santo Tomas now has the beginnings of a screening program, which will be complemented by a perinatal infectious disease program that will provide previously unavailable medicines to mothers and their babies and follow any congenitally infected children. Finally, there is an emergence of education and research initiatives that strive to bring awareness to toxoplasmosis at all levels - starting from the pregnant women themselves, through distributing informational pamphlets at maternity halls, to medical personnel such as doctors and nurses through educational talks.

References

1. McLeod, Rima. "An Overview of Toxoplasmosis: Cause, Prevalence, and Consequences." *TRI: Information about Toxoplasmosis*. Toxoplasmosis Research Institute and Center, n.d. Web. 15 Sept. 2016.

2. Torgerson, P, and Mastroiacovo, P. The global burden of congenital toxoplasmosis: a systematic review. World Health Organization. *Bull World Health Organ*. 2008;91(7):501-508.

3. Montoya JG, Liesenfeld O (2014) Toxoplasmosis. Lancet. 363(9425):1965–76. < https://www.ncbi.nlm.nih.gov/pubmed/15194258>

4. McLeod R, Boyer K. Toxoplasmosis (Toxoplasma gondii). Nelson Textbook of Pediatrics 20th ed, eds Kliegman R, Stanton B, St. Geme J (Elsevier), pp 494-504.

5. Fallahi S, Rostami A, Nourollahpour S, Behniafar H, Paktinat S (2018) An updated literature review on maternal-fetal and reproductive disorders of Toxoplasma gondii infection. J Gynecol Obstet Hum Reprod 47(3): 133-140. < https://www.ncbi.nlm.nih.gov/pubmed/29229361>

6. Pappas G, Roussos N, Falagas ME (2009) Toxoplasmosis snapshots: global status of toxoplasma gondii seroprevalence and implications for pregnancy and congenital toxoplasmosis. Int J Parasitol. 39(12):1385–94. <https://www.ncbi.nlm.nih.gov/pubmed/19433092 >

7. Decreto Ejecutivo No. 1617, 2014

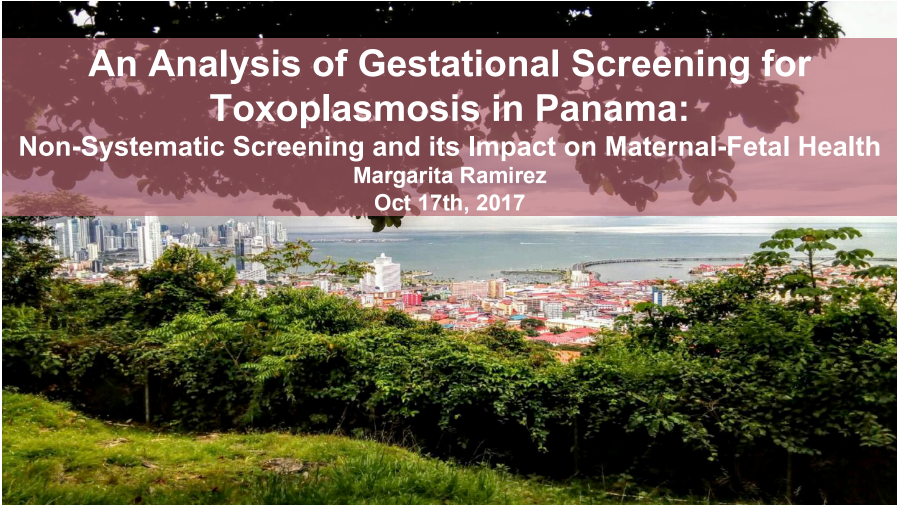
An Analysis of Gestational Screening for
Toxoplasmosis in Panama:
Non-Systematic Screening and its Impact on Maternal-Fetal Health
Margarita Ramirez
Oct 17th, 2017

Background

Acute & Congenital Infection

Screening, Serologies, & Treatment

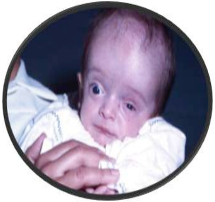

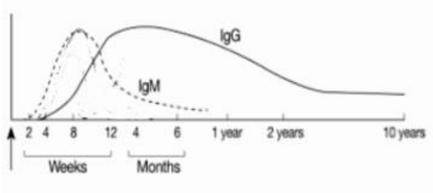

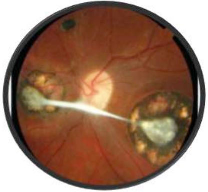

Research Objective

Overall objective: characterization of acute & congenital *Toxoplasma gondii* infection in Panama City

Specifically, characterize:

- Differences between screened & unscreened women
- Acutely infected mothers
- Clinical manifestations of congenitally infected babies

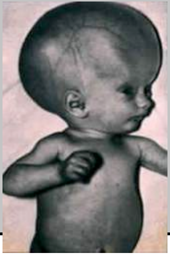

Methods

- Surveys and blood samples from 343 pregnant women
- Medical charts of 6 congenitally infected babies

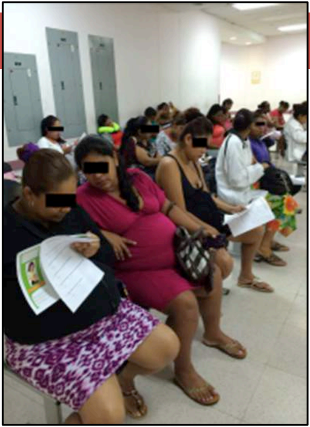

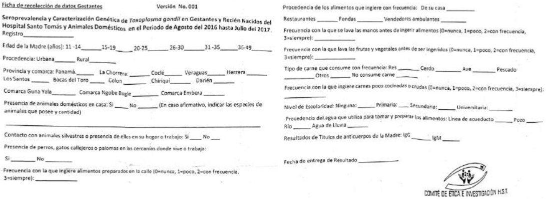

Ficha de recolección de datos Gestantes   Versión No. 001

Seroprevalencia y Caracterización Genética de *Toxoplasma gondii* en Gestantes y Recién Nacidos del Hospital Santo Tomás y Animales Domésticos en el Periodo de Agosto del 2016 hasta Julio del 2017.

Registro ____________

Edad de la Madre (años): 11 -14 ______ 15-19 ____ 20-25 ______ 26-30 ______ 31 -35 ______ 36-49 ______

Procedencia: Urbana ______ Rural ______

Provincia y comarca: Panamá ______ La Chorrera: ______ Coclé ______ Veraguas ______ Herrera ______ Los Santos ______ Bocas del Toro ______ Colon ______ Chiriquí ______ Darién ______

Comarca Guna Yala ______ Comarca Ngobe Bugle ______ Comarca Embera ______

Presencia de animales domésticos en casa: Si ____ No ______ (En caso afirmativo, indicar las especies de animales que posee y cantidad)

Contacto con animales silvestres o presencia de ellos en su hogar o trabajo: Si ____ No ___

Presencia de perros, gatos callejeros o palomas en las cercanías donde vive o trabaja:

Si ______ No ______

Frecuencia con la que ingiere alimentos preparados en la calle (0=nunca, 1=poco, 2=con frecuencia, 3=siempre): ____________

Procedencia de los alimentos que ingiere con frecuencia: De su casa ________

Restaurantes ______ Fondas ______ Vendedores ambulantes ______

Frecuencia con la que se lava las manos antes de ingerir alimentos (0=nunca, 1=poco, 2=con frecuencia, 3=siempre): ____________

Frecuencia con la que lava las frutas y vegetales antes de ser ingeridos (0=nunca, 1=poco, 2=con frecuencia, 3=siempre): ____________

Tipo de carne que consume con frecuencia: Res ______ Cerdo ______ Ave ______ Pescado ______ Otros ______ No consume carne ______

Frecuencia con la que ingiere carnes poco cocinadas o crudas (0=nunca, 1=poco, 2=con frecuencia, 3=siempre): ____________

Nivel de Escolaridad: Ninguna: ______ Primaria: ____ Secundaria: ______ Universitaria: ______

Procedencia del agua que utiliza para tomar y preparar los alimentos: Línea de acueducto ______ Pozo ______ Río ______ Agua de Lluvia ______

Resultados de Títulos de anticuerpos de la Madre: IgG ______ IgM ______

Fecha de entrega de Resultado ____________

COMITÉ DE ÉTICA E INVESTIGACIÓN H.S.T.

IgM+ Women

Age

	IgM+	Cohort
Avg Age 2017 (yrs)	25.57 ± 9.03	27.28 ± 6.23
Avg Age 2016 (yrs)	24.43 ± 7.24	26.59 ± 7.15

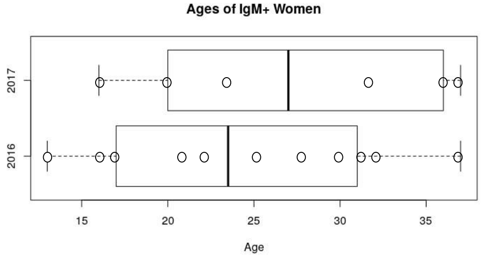

IgM+ Women

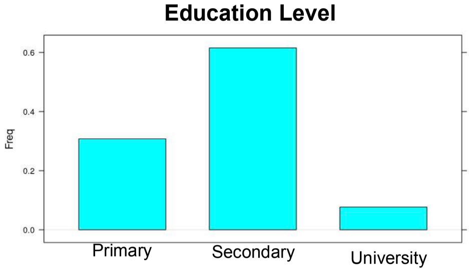

Other:

- Half had pets, but only 1 had a cat
- All have plumbing

Map of IgM+ Women

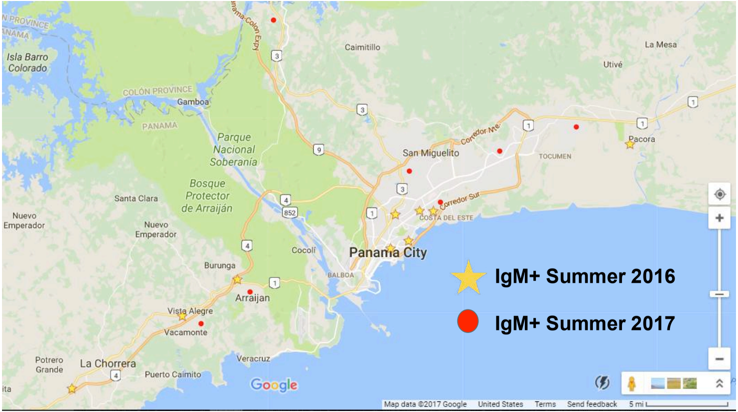

Results:

Differences between Screened & Unscreened

80% of the women had yet to be screened

Education **(P = 0.07)**

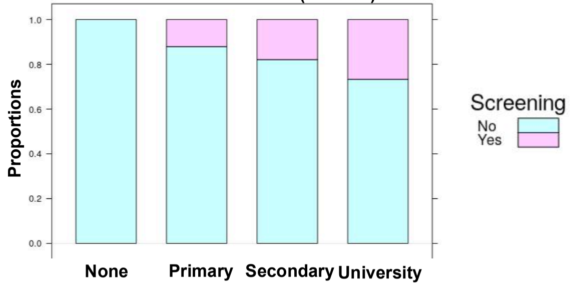

Screening Map

Summer 2017

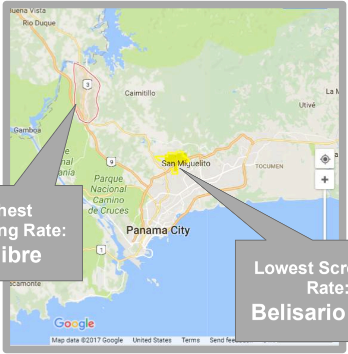

Highest Screening Rate:
Chilibre

Lowest Screening Rate:
Belisario Frias

Summer 2016

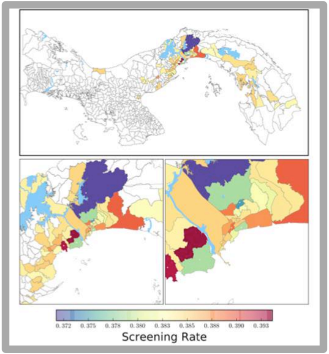

Nuances of Screening

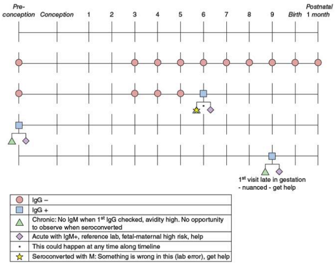

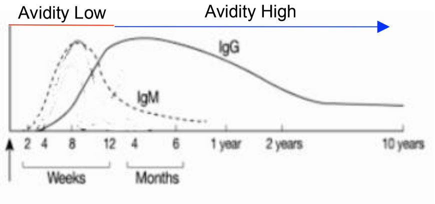

23 women were IgM -, IgM+ → IgG +, IgM-
Regular Screening is Important

Medical Charts

Pt No.	Gest. Age (wks)	Pre Rx	Clinical Manifestations				Post natal Treatment		
			Gen.	Eye	Brain	Other	Pyri	Clin	Fol Ac
1	38	Sp							
2	41				H; M; > 3 cal.	J			
3	37		R/O	Mac		cardio.			
4	37			Mac	HC; TC <3 cal	J			
5	37		DI	hem. Od	V	+ herp. Death			
6	37		DI		M; E	HT			

Key

Blue - Mild/Moderate
- R/O = R/O sepsis
- J = Jaundice
- < 3 cal = < 3 cerebral calcifications

Red - Severe
- DI =Diabetes Insipidus
- Mac = Macular Chorioretinitis
- H = Hydrocephalus
- M = Microcephalus
- V= Ventriculomegaly
- TC = Thin Cortex
- E = Epilepsy

Received Treatment

Not given/Not found

Conclusion

Accomplishments & Benefits

- Screening Program
- Perinatal ID Program
- Education & Research Initiatives

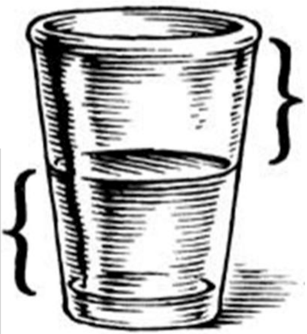

Non-systematic screening remains a problem

- Delays accurate diagnosis
- Delays/inhibits appropriate treatment

Other Programs & Research

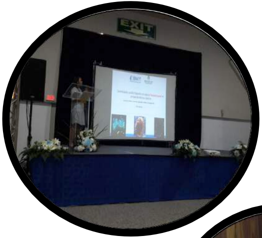

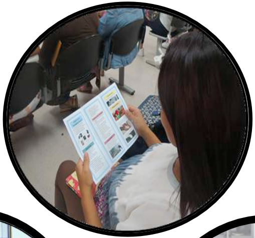

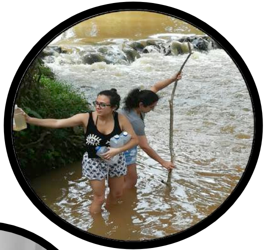

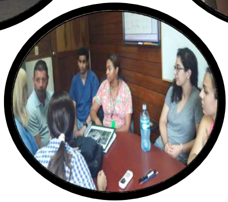

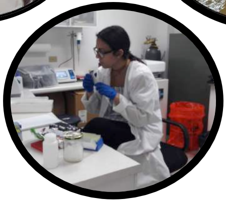

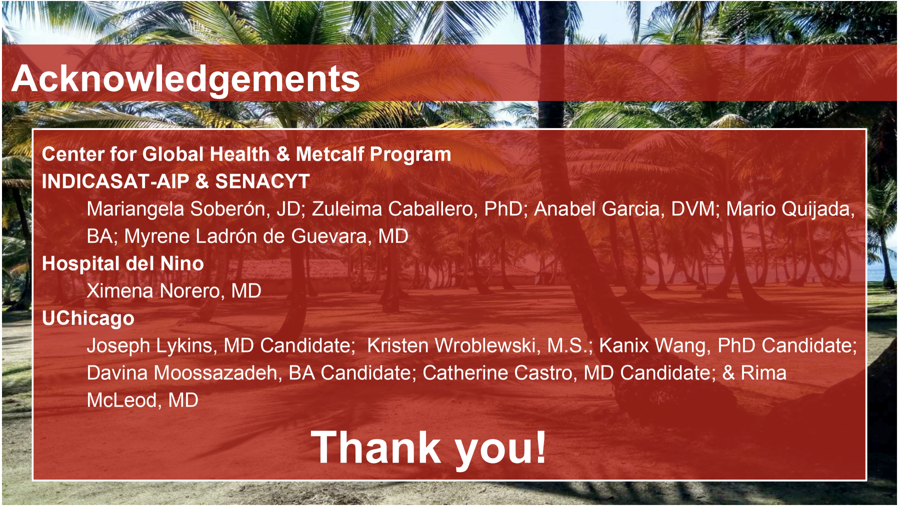
Acknowledgements
Center for Global Health & Metcalf Program
INDICASAT-AIP & SENACYT
Mariangela Soberón, JD; Zuleima Caballero, PhD; Anabel Garcia, DVM; Mario Quijada, BA; Myrene Ladrón de Guevara, MD
Hospital del Nino
Ximena Norero, MD
UChicago
Joseph Lykins, MD Candidate; Kristen Wroblewski, M.S.; Kanix Wang, PhD Candidate; Davina Moossazadeh, BA Candidate; Catherine Castro, MD Candidate; & Rima McLeod, MD
Thank you!

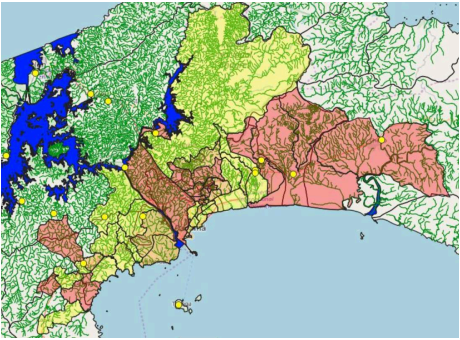

Catalina Raggi
with José Sánchez; Jorge Enrique Gómez Marín, MD; Zuleima Caballero, PhD; Rima McLeod, MD; and Instituto de Investigaciones Científicas (INDICASAT) – Panama; Grupo de Investigación en Parasitología Molecular (GEPAMOL), Universidad del Quindío – Colombia

A Spatial and Statistical Review and Analysis of Toxoplasmosis Screening and Seroprevalence in Colombia and Panama

Cat Raggi*
The College at the University of Chicago
*In collaboration with Jose Sanchez, Jorge Enrique Gómez Marin, Zuleima del Carmen Caballero, Alejandra de-la-Torre, Juliana Muñoz, Alejandro Acosta Dávila, Laura Lorena López, Elizabeth Torres, Juan David Valencia, Manuela Mejia Oquendo, Daniel Celis, Mónica Vargas, Delba Villalobos, María Cristina Bohórquez, José Yashin Artega Rivera, Nicolás Rivera Valdivia, Mariangela Soberon Felin, Abhi Pandya, Aliya Moreira, Kanix Wang, Stefany Velasco, Daniel Celis Giraldo, Catherine Castro, Margarita Ramirez, Davina Moosazadeh, Osvaldo Reyes, Mayrene Ladrón de Guevara, Connie Mendivil, Mario L. Quijada, Anabel García, Guillermo Pradieu, Dora Estripeaut, Ximena Norera, Carlos Flores, Jovanna Borace, Andrey Rzhetsky, Sharon Heichman, Rima McLeod

Introduction

Background

- Toxoplasmosis is a parasitic disease
- Breeds in feline intestinal tracts
- Can be found in soil, water, and near animal feces

Objectives

- Identify risk factors for toxoplasmosis in the general population in Armenia
- Identify risk factors for ocular lesions in patients with toxoplasmosis in Armenia
- Locate areas of congenital toxoplasmosis and see if the spatial distribution changes based on prenatal treatment
- Locate areas of lymph node cases related to toxoplasmosis and see if the spatial distribution changes based on sex.
- Locate areas of particularly high or low toxoplasmosis screening and seroprevalence in Panama's corregimientos and look for spatial clustering.

Methods

Armenia, Quindio, Colombia

- Worked with the University of Quindio
- Congenital and Lymphadenopathic toxoplasmosis from their medical records
- More recent survey done in two neighborhoods of the city, La Universal and Guaduales de la Villa
- Offered free toxoplasmosis blood tests, ocular exams, and a survey

Panama City, Panama

- Worked with INDICASAT
- Surveys and free blood tests and the hospitals of San Juan and Santo Tomas
- Free toxoplasmosis blood tests and surveys
- Information about water sources taken from Tommy Guardia

Data Analysis

- Multivariate logistic regression for risk factor analysis
- Kernel Density Estimator maps for spatial coordinates
- Polygon heat maps for Panamanian corregimientos with at least 5 individuals
  - Natural break
  - Standard deviation
- Moran's I Cluster analysis with a first order queen weight

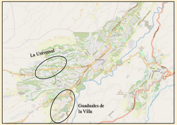

Results

Prenatal Treatment in Congenital Cases in Armenia

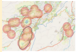

Cases of congenital toxoplasmosis with prenatal treatment found (n=21)

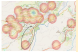

Cases of congenital toxoplasmosis without prenatal treatment found (n=23)

Prenatal treatment higher in Guaduales de la Villa (7/8)

Lymph Node Impairments by Sex in Armenia

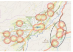

Lymph Node Impairments Related to Toxoplasmosis in Men (n=17)

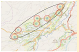

Lymph Node Impairments Related to Toxoplasmosis in Females (n=7)

Women mostly in North, all cases in Calarca were male

Toxoplasmosis Screening in Panama Oeste

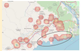

Previously Unscreened Individuals (n=70)

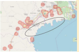

Previously Screened Individuals (n=60)

Screening closer to highway, unscreened coasts

Toxoplasmosis Screening in Panama

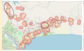

Previously Unscreened Individuals (n=190)

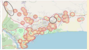

Previously Screened Individuals (n=88)

More unscreened up north, all screened in Pedregal

Toxoplasmosis Screening in Panamanian Corregimientos

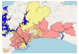

Natural Break Heatmap of Screening Rates

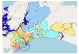

Standard Deviation Heatmap of Screening Rates

Veracruz as an outlier, high screening in Vista Allegre

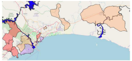

Significant Clusters in a Moran's I Analysis of Screening Rates in Panamanian Corregimientos with a 1st Order Queen Weight

Western cluster, potential outliers

Toxoplasmosis Seroprevalence in Panamanian Corregimientos

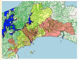

Natural Break Heatmap of Seroprevalence Rates with Hydrologic systems

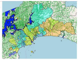

Standard Deviation Heatmap of Seroprevalence Rates with Hydrologic systems

Higher in Easternmost corregimientos

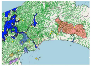

Significant Clusters in a Moran's I Analysis of Seroprevalence Rates in Panamanian Corregimientos with a 1st Order Queen Weight

Low clusters in west, high clusters in east

Risk Factors Analysis in La Universal

Variable	Est	Std	P
Intercept	0.046044	0.856228	0.9571
Age	0.019913	0.011751	0.0901
Bottled Water	-0.471155	0.574321	0.4120
Using Unboiled Water	-0.801122	0.696129	0.2498
Drinking Unboiled Water	0.239532	0.672934	0.7219
Drinking River Water	0.984534	1.142554	0.3889
Using Water from the Tap	0.468942	0.725362	0.5180
Eating Undercooked Meat	-0.491330	0.734809	0.5037
Eating at Restaurants	0.004399	0.522440	0.993
Cats in the Home	-0.305260	0.542255	0.5735
Eating Unwashed Vegetables	-0.367854	0.539691	0.4955

Risk Factors Analysis in Guaduales De La Villa

Var	Est	Std	P
Intercept	1.619585	1.085696	0.1358
**Age**	**0.047347**	**0.019742**	**0.0165**
**Bottled Water**	**-2.237384**	**0.903107**	**0.0132**
Using Unboiled Water	0.007577	1.148602	0.9947
Using Unboiled Water	0.600856	1.066240	0.5731
Drinking River Water	-2.878850	1.494916	0.0541
Using Water from the Tap	-0.257427	0.857117	0.7639
**Eating Undercooked Meat**	**-1.916415**	**0.901556**	**0.0335**
Eating at Restaurants	-0.052448	0.746456	0.9440
Cats in the Home	0.149600	0.776652	0.8473
Eating Unwashed Vegetables	-2.160195	1.127538	0.0554

Conclusion

- Negative correlation between eating undercooked meat and seroprevalence
- Negative correlation between drinking bottled water and seroprevalence
- Prenatal treatment appears to be higher in Guaduales de la Villa than anywhere else in Armenia
- The spatial distribution of screening in Panama Oeste appears to be more closely flowing the highway Arraijan than the unscreened distribution
- Individuals from coastal areas in Panama Oeste have mostly been previously unscreened
- Regions like Pedregal in Panama may not be accessing the same screening services as other regions
- The corregimiento of Veracruz may be a spatial outlier for screening
- Vista Alegre has a particularly high screening rate
- Screening rates may be higher in western Panama
- Seroprevalence is higher in eastern Panama

Acknowledgements

Thank you to everyone at the Center for Global Health, GEPAMOL at the University of Quindio and at INDICASAT as well as University of Chicago students for their past work!

ABSTRACT:

Goal: The purpose of this study was to identify areas where congenital, ocular, and lymphadenopathic toxoplasmosis were focused in Armenia, Colombia and any risk factors that may be associated. In addition, rates of seroprevalence and gestational screening in Panamanian corregimientos were mapped and observed in relation to their water sources.

Introduction: Toxoplasmosis is a parasitic infection that can cause a condition known as congenital toxoplasmosis in an infant if initial infection occurs during a woman's pregnancy. The infection, while often asymptomatic in the immunologically competent adult, can also manifest complications such as ocular lesions, lymphadenopathy, and severe disease in immunologically compromised persons.

Methods: Data for this study was taken from surveys and medical records from the University of Quindio, a survey of pregnant women from Panama's INDICASAT, and past studies done with University of Chicago and INDICASAT at Hospital Santo Tomas in Panama City. Water data was taken from digitized Tommy Guardia sources. The analysis was done using both qualitative and quantitative spatial methods, as well as log odds regression. If point data was available, kernel density estimator heatmaps were created and compared to look at spatial distributions. When possible, data points were also condensed and summarized into regional data for polygon based analyses such as natural break and standard deviation heatmaps, and a Moran's I cluster analysis to look for spatial clustering or outliers.

Results: The risk factor analysis in Armenia found that drinking from bottled water and eating undercooked meat were protective factors in one of the two communities tested. An exploration of the point data suggests various potential spatial differences in prenatal treatment for congenital toxoplasmosis in Armenia, as well as in screening in the Panamanian provinces of Panama and Panama Oeste. The analyses suggest a cluster of high screening rates in the westernmost corregimientos surveyed and a particularly high screening rate in Vista Alegre. Toxoplasmosis seroprevalence also appears to be highest in eastern corregimientos.

Conclusion: These statistical analyses and explorations can be used to identify geographic trends, advise future preventative measures and policies, and locate areas in which more localized studies may provide useful results.

GOALS:

- Identify risk factors for toxoplasmosis in the general population in Armenia, Colombia and compare it to past years results to risk factors for pregnant women in Panama City, Panama.
- Identify risk factors for ocular lesions in patients with toxoplasmosis in Armenia
- Locate areas of congenital toxoplasmosis in Armenia and see if the spatial distribution changes based on prenatal treatment.
- Locate areas of lymph node cases related to toxoplasmosis and see if the spatial distribution changes based on sex.
- Locate potential areas of high toxoplasmosis risk in Armenia and Panama City
- Locate areas of particularly high or low toxoplasmosis screening in Panama's corregimientos, compare it to past years, and look for spatial clustering.
- Locate areas of particularly high or low toxoplasmosis seroprevalence in Panama's corregimientos, compare it to past years, and look for spatial clustering.
- Investigate the relationship between toxoplasmosis seroprevalence in Panama and water sources.

BACKGROUND:

Toxoplasmosis is a disease caused by the parasite Toxoplasmosis gondii. The parasite grows in the intestinal system of cats, and can be spread through the ingestion of oocysts spread through contaminated food and water. Although often asymptomatic, toxoplasmosis can cause ocular lesions and impair eyesight. In addition, if made active during a woman's pregnancy, the fetus may contract what is known as congenital toxoplasmosis, facing potential developmental impairment throughout life. The effects of this can be mitigated through proper treatment that increases in efficacy the sooner it is administered. With this in mind, screening for the presence of Toxoplasmosis gondii, particularly early on in a woman's pregnancy, is vital. The parasite can be found through the presence of IgG and IgM antibodies, where IgM antibodies indicate a more recent or reactivated infection that poses a higher threat to the child. The prevalence of this

infection vary widely throughout the world, although it is believed to be relatively high in the areas surrounding Panama City, Panama, Panama and in Armenia, Quindio, Colombia.

METHODS:

This project involved 3 weeks in Armenia, Colombia with the University of Quindio's GEPAMOL group under Dr. Jorge Gomez followed by 10 days in Panama City, Panama with INDICASAT. While the limited duration of the stay allowed for little data collection, we worked with members of both parties for data analysis.

Much of this analysis focuses on the spatial aspect, namely using point and polygon vector data. Based on directions taken from survey participants, coordinate locations were found to best approximate the participant's location, typically with a coordinate representing the center of their block. For this point data, a Kernel Density Estimator (KDE) analysis was used through QGIS with a quartic distribution. Although this is a spatially extensive variable analysis, three heatmaps created in this manner, two showing the spread and clustering of opposing data (i.e. the distribution of IgG positive and IgG negative individuals in a population) and one showing the spread of all individuals, regardless of their status, were compared. While the KDE analysis in itself doesn't account for population clustering, one would assume that the groups being compared would have similar regions of clustering if the results were solely due to the spread of population density.

In cases where regional data was known, such as the corregimiento of an individual in Panama, the information was aggregated to collect general information for the region. Using this data, natural break and standard deviation choropleth maps were created to show regional clustering and areas of particularly high or low values. In cases where few people came from a particular corregimiento, regions with fewer than 5 participants were discarded for the aggregated spatial analysis.

In addition, a Moran's I analysis was done on much of the spatial data to identify any clusters or spatial outliers. For point data, a K-neighbors weight was used, typically with K set as 4 or 5, and a first order queen weight was used for polygon data. This clustering was considered

significant at a 0.05 pseudo p value. Both the choropleth maps and the Moran's I analysis were calculated through Geoda and then overlaid into QGis.

The risk factor analysis of Armenia was done through data taken a University of Quindio's study in which they set up temporary clinics in two locations in Armenia, la Universal and Guaduales de la Villa, and gave free eye exams, surveys, and testing for toxoplasmosis. As individuals waited to be tested and examined, questions were asked orally about their behaviour regarding suspected toxoplasmosis risk factors. These questions were taken as indicator variables and added to a spreadsheet which included their addresses, the results of the toxoplasmosis exam, and if any ocular lesions were found during the exam. This spreadsheet was then analyzed in RStudio using a log odds linear regression to identify risk factors in Armenia as a whole as well as in each community tested.

Table 1: Variables Used for the Risk Factor Analysis

**Variable Name**	**Coded Name**	**Description**	**Type of Variable**	**Range**	**Mean**	**SD**
IgG	IgG	Whether or not the individual is positive for IgG antibodies	Indicator	0-1	0.691824	0.464199
Use unboiled water	AgSinHerv	Whether the individual uses water without boiling it	Indicator	0-1	0.35625	0.480394
Drink unboiled water	BebSinHerv	Whether the individual uses water without boilng it	Indicator	0-1	0.45	0.499

Bottled water	Botella	Whether the individual frequently drinks bottled water	Indicator	0-1	0.25625	0.437932
Undercooked meat	CarMedOcc	Whether the individual eats undercooked meat	Indicator	0-1	0.1625	0.3701
Restaurants	ComeRest	Whether the individual frequently eats out	Indicator	0-1	0.475	0.500942
Age	Edad	Age of the individual in years	Numeric	1-91	46.7826	22.5787
Cats	Gatos	Whether the individual has cats in the home(although	Indicator	0-1	0.3125	0.4649
Lesions	Lesiones	Whether an ocular exam has shown the presence of lesions	Indicator	0-1	0.111801	0.316105

Tap water	AgLlave	Whether the individual drinks tap water not boiled	Indicator	0-1	0.40625	0.49267
River	AgRios	Whether the individual drinks water from a river	Indicator	0-1	0.06875	0.253823
Unwashed vegetables	VegSinLav	Whether the individual eats vegetables without washing them	Indicator	0-1	0.38125	0.487219

In Panama, data regarding toxoplasmosis seroprevalence and previous screening for the infection was taken from work done by University of Chicago students in previous years and by researchers in INDICASAT in recent months. Locations and outlines of Panama's hydraulic systems and water basins were found as digitized Tommy Guardia maps by the Smithsonian Tropical Research Institute. Similarly, water treatment plants run by Panama's Institute of Aqueducts and Sewage Systems (IDAAN) were copied from the IDAAN website. The seroprevalence and screening rates of each corregimiento were then used to make standard deviation, natural break, and Moran's I cluster maps in Geoda that were overlaid with the water system information in QGis. This was used to try to identify any potential overlap with toxoplasmosis rates and both screening and water sources.

RESULTS AND DISCUSSION:

A scatterplot matrix of the various potential risk factors investigated in the Armenian survey showed various interesting relationships, both in the city as a whole and in each of the two communities.

Figure 1: Scatterplot matrix of all of the variables used in the Armenian study using all participating individuals

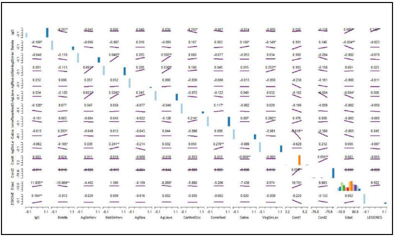

Table 2: Statistically significant variable relations and their p value ranges for the entire population surveyed

	Positive	Negative
$0.01 < p < 0.05$	• Bottled water + Cats<br>• Undercooked meat + Restaurants	• IgG + Bottled water<br>• IgG + Undercooked meat<br>• Bottled Water + Unwashed vegetables

		• Bottled water + Tap Water
P < 0.01	• IgG + Lesions<br>• Drink + Use unboiled water<br>• Drink unboiled water + Tap water<br>• Drink unboiled water + Unwashed vegetables<br>• River + Cats<br>• Restaurant + Unwashed vegetables<br>• IgG + Age	• Bottled Water + Age

Figure 2: Scatterplot matrix of all of the variables used in the Armenian study using individuals from community in La Universal

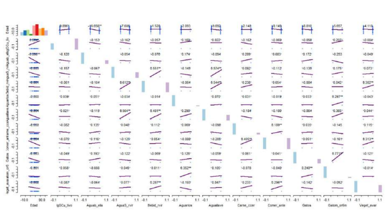

Table 3: Statistically significant variable relations and their p value ranges in La Universal

	Positive	Negative
0.01<p<0.05	• Age + IgG	
p< 0.01	• Tap water + Use unboiled water<br>• Cats + Unwashed vegetables<br>• Restaurants + Unwashed vegetables<br>• Restaurants + Undercooked meat<br>• Drink unboiled water + Unwashed vegetables<br>• Drink unboiled water + Tap water	• Age + Bottled Water

Figure 3: Scatterplot matrix of all of the variables used in the Armenian study using individuals from community in Guaduales de la Villa

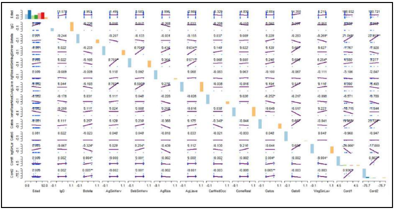

Table 4: Statistically significant variable relations and their p value ranges in Guaduales de la Villa

	Positive	Negative
0.01<p<0.05	• Drink unboiled water + Unwashed vegetables	• Bottled water + Unwashed vegetables<br>• Undercooked meat + Cats
p<0.01	• Use unboiled water + Tap water<br>• Use unboiled water + Drink unboiled water<br>• Drink unboiled water + Tap water	

These scatterplots show a variety of potentially useful or interesting information. One of the more notable relationships in this dataset is that between IgG seropositivity and the presence of ocular lesions, which only occurred alongside the presence of IgG antibodies. This contradicts certain literature that argues that acquired toxoplasmosis plays a minor role in the formation of ocular lesions.

A more predictable but still notable relationship is that between drinking and using unboiled water and also drinking tap water. Although this study did not pinpoint any of the three as significant risk factors given this data, water is a known risk factor for toxoplasmosis in many locations, and this suggested connection between these variables may be of interest for future education programs.

A chi square analysis on a contingency table with IgG positivity and the community failed to show a significant relationship between the two, showing a chi square value of 1.6099 an a p-value of 0.2045. A similar analysis between the presence of lesions in those found to be IgG positive and the community also showed similar results with a chi square value of 0.88567 and a p value of 0.3467.

To analyze the significance of the risk factors, a log odds regression was done using the data from both communities to see if there were any differences. This included both a single variable log odds regression with each variable, and multivariable linear regression using all of them as predicting variables.

For the community of La Universal, no variables except for age, were found to be significant under a 0.05 p value. As toxoplasmosis remains in the system indefinitely, it makes sense that as one gets older, their probability of being positive for IgG increases.

Table 5: Variable Estimates and P-Values in Each Simple Regression for La Universal

Var	Estimate	Std	P
**Edad**	**0.02493**	**0.01036**	**0.0161**
AgLlave	0.09531	0.46953	0.8391

AgRios	1.0361	1.1182	0.3542
AgSinHerv	-0.4678	0.4571	0.30608
BebSinHerv	-0.07551	0.43912	0.8635
Botella	-0.8473	0.4998	0.09005
CarMedOcc	-0.7309	0.6248	0.2420
ComeRest	-0.3934	0.4397	0.37095
Gatos	-0.4520	0.4769	-0.34324
VegSinLav	-0.3677	0.4475	0.41122

The multivariable linear regression showed similar results, although age no longer appears to be a significant factor. Given the results from the scatterplot matrix with La Universal's data, this may be at least partially due to the possible relationship between age and drinking bottled water.

Table 6: Variable Estimates and P-Values for a Multivariable Regression for La Universal

Var	Est	Std	P
Intercept	0.046044	0.856228	0.9571
Edad	0.019913	0.011751	0.0901
Botella	-0.471155	0.574321	0.4120
AgSinHerv	-0.801122	0.696129	0.2498
BebSinHerv	0.239532	0.672934	0.7219

AgRios	0.984534	1.142554	0.3889
AgLlave	0.468942	0.725362	0.5180
CarMedOcc	-0.491330	0.734809	0.5037
ComeRest	0.004399	0.522440	0.993
Gatos	-0.305260	0.542255	0.5735
VegSinLav	-0.367854	0.539691	0.4955

Guaduales de La Villa showed the opposite behavior, and while drinking bottled water and eating undercooked meat were deemed insignificant by the single variable regression with p values of 0.059 and 0.069 respectively, a multivariable analysis then shows drinking bottled water and eating undercooked meat as protective factors. This is a strange result as eating undercooked meat is typically found to increase ones chances for acquiring toxoplasmosis due to potentially consuming parasitic oocysts.

Table 7: Variable Estimates and P-Values in Each Simple Regression for Guaduales de La Villa

Var	Est	Std	P
**Edad**	**0.03369**	**0.01487**	**0.0235**
AgSinHerv	0.1494	0.5861	0.79883
AgRios	-0.7151	0.9601	0.456352
AgLlave	0.004819	0.563960	0.9932
BebSinHerv	0.1519	0.5704	0.79003
Botella	-1.1219	0.5944	0.059107

CarMedOcc	-1.1856	0.6513	0.0687
ComeRest	-0.4793	0.5699	0.40036
Gatos	0.4187	0.6077	0.4908
VegSinLav	-0.1001	0.5734	0.86145

Table 8: Variable Estimates and P-Values for a Multivariable Regression for Guaduales de la Villa

**Var**	**Est**	**Std**	**CI**	**Odds Ratio CI**	**P**
Intercept	1.619585	1.085696			0.1358
**Edad**	**0.047347**	**0.019742**	**(0.00865268, 0.08604)**	**(1.00869, 1.08985)**	**0.0165**
**Botella**	**-2.237384**	**0.903107**	**(-4.007, -0.4673)**	**(0.01819, 0.6267**	**0.0132**
AgSinHerv	0.007577	1.148602			0.9947
BebSinHerv	0.600856	1.066240			0.5731
AgRios	*-2.878850*	*1.494916*			*0.0541*
AgLlave	-0.257427	0.857117			0.7639
**CarMedOcc**	**-1.916415**	**0.901556**	**(-3.683, -0.1493)**	**(0.0251, 0.8612544)**	**0.0335**
ComeRest	-0.052448	0.746456			0.9440

Gatos	0.149600	0.776652			0.8473
VegSinLav	*-2.160195*	*1.127538*			*0.0554*

Data regarding the relationship between these risk factors and the presence of lesions given toxoplasmosis seropositivity was analyzed as well. No variables were found to be associated with lesions in any of the analyses.

Table 9: Variable Estimates and P-Values for Each Simple Regression for Ocular Lesions

Var	Est	Std	P
Edad	0.01393	0.01286	0.27873
Botella	0.2350	0.6292	0.709
AgSinHerv	-0.2469	0.5755	0.668
BebSinHerv	0.1005	0.5293	0.849
AgRios	-0.2758	1.1032	0.803
AgLlave	0.04994	0.53604	0.926
CarMedOcc	-0.01835	0.81845	0.982
ComeRest	0.1005	0.5293	0.849
Gatos	0.2205	0.5558	0.692
VegSinLavar	-0.3878	0.5740	0.499

Table 10: Variable Estimates and P-Values for a Multivariable Regression for Ocular Lesions

**Var**	**Est**	**Std**	**P**

Edad	0.01423	0.01435	0.321
Botella	0.25609	0.68292	0.708
AgSinHerv	-0.40880	0.90581	0.652
BebSinHerv	0.46789	0.83824	0.577
AgRios	-0.37506	1.14358	0.743
AgLlave	0.06574	0.82580	0.937
CarMedOcc	-0.09009	0.89146	0.92
ComeRest	0.24967	0.58669	0.670
Gatos	0.13346	0.59885	0.824
VegSinLav	-0.58250	0.65548	0.374

Congenital toxoplasmosis cases were collected through the span of multiple years and then mapped based on whether or not they had received prenatal treatment.

Figure 4: Kernel Density Map of Congenital Toxoplasmosis in Armenia

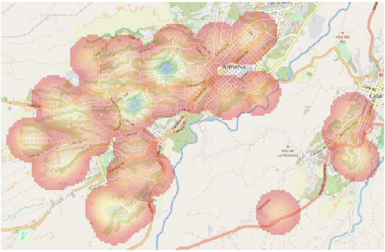

All cases of congenital toxoplasmosis found (n=47)

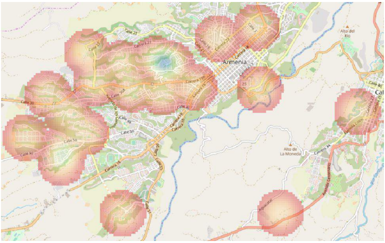

All cases of congenital toxoplasmosis without prenatal treatment found (n=23)

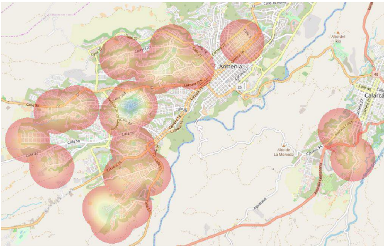

All cases of congenital toxoplasmosis with prenatal treatment found (n=21)

In the community of Guaduales de la Villa, there appears to be a much higher concentration of prenatal treatment in congenital toxoplasmosis than in the rest of Armenia. Of the 8 points around that community, 7 have been treated (87.5), which is markedly different from the 21 treated locations from the 44 points with known treatment statuses (47.7%). This difference may be due to the source of the data. As Guaduales de la Villa is further away from the University of Quindio, it may be that they are simply more likely to report to the university after the diagnosis has been made with the goal to seek treatment.

Figure 5: Kernel Density Map of Lymphadenopathic Toxoplasmosis in Armenia

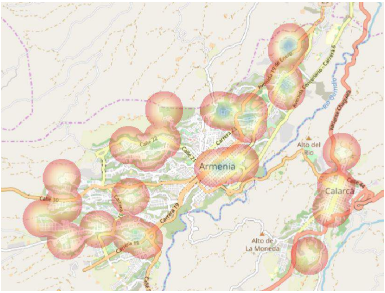

Map of Lymph Node Impairments Related to Toxoplasmosis in Armenia, Quindio, Colombia

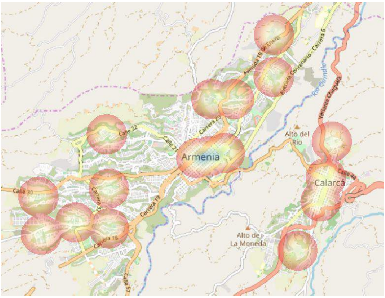

Map of Lymph Node Impairments Related to Toxoplasmosis in Men in Armenia, Quindio, Colombia (n=17)

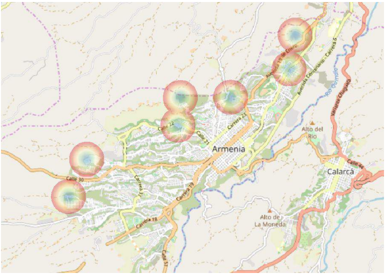

Map of Lymph Node Impairments Related to Toxoplasmosis in Females in Armenia, Quindio, Colombia (n=7)

Lymphadenopathy is a known complication of toxoplasmosis. Locations of cases of this known by the University of Quindio were mapped based on sex to look for any spatial disparities for this based on sex. Unfortunately, as the cases of toxoplasmosis without the presence of lymphadenopathy were not included in the records given, no analysis can be done comparing the seroprevalence between the two sexes.

In addition, the very limited data size means no serious conclusions can be drawn from this data. However, the very uneven distribution of cases between the sexes, with all of the cases from Calarca being male and all female cases being in Armenia’s north border, may warrant further study.

Figure 6: Kernel Density Map of Cats in Armenia Based on Toxoplasmosis Status

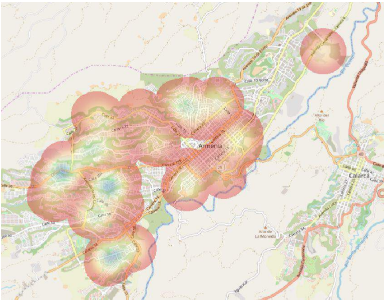

Map showing density of cats studied by the University of Quindio (n=134)

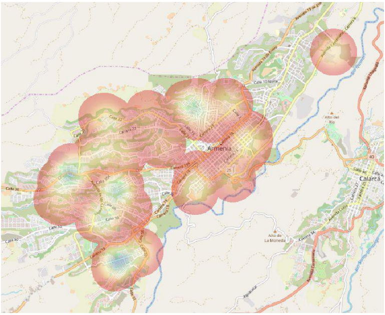

Map showing density of Toxo Negative cats studied by the University of Quindio (n=109)

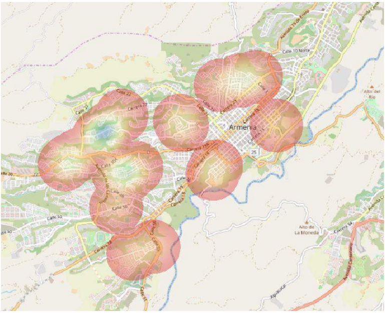

Map showing density of Toxo Positive cats studied by the University of Quindio (n=25)

In order to understand toxoplasmosis in Armenia, it may be useful to have an idea of the distribution of cats in the region as past studies in other locations have named them as a potential cause of infection. The above maps visualize the distribution of all cats in Armenia as well as these cats base on their toxoplasmosis infection status. Comparing the last two maps shown above, it does not appear that there is any serious difference between the spatial distributions of IgG negative and positive cats in Armenia.

Figure 7: Kernel Density Maps for Screening for Toxoplasmosis in the Province of Panama

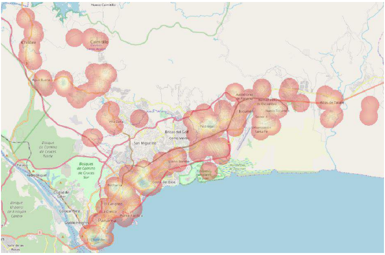

KDE of Previously Unscreened Individuals in the Panama Province (n=190)

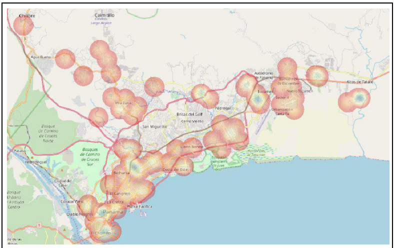

KDE of Previously Screened Individuals in the Panama Province (n=88)

The above figure shows a KDE heatmap of individuals who had previously been screened for toxoplasmosis in their pregnancy based on data collection done by University of Chicago students in 2016. The image on the left shows the distribution and concentration of the people in Panama who had not been screened for toxoplasmosis and the one of the right has those who had been previously screened. The two figures seem to have a different spread, with the one of the left having a larger amount of points on the east and more points up north. Meanwhile the image showing the people who had been previously screened is more focused on the west closer to the canal. The KDE for unscreened individuals also appears to have a cluster around the region of Pedregal with roughly 11 points, which is somewhat striking as there are no points in that region for any screened individuals, suggesting

Figure 8: Kernel Density Maps for Screening for Toxoplasmosis in the Province of Panama Oeste

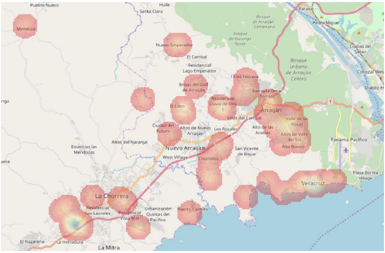

KDE of Previously Unscreened Individuals in the Province of Panama Oeste (n=70)

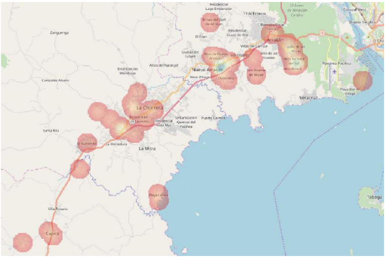

KDE of Previously Screened Individuals in the Province of Panama Oeste (n=60)

These maps show quite a few trends regarding screening rates in Panama Oeste. For one, there is a cluster of screened individuals in the center of a region called Nuevo Arraijan that appears to be partially surrounded by non screened individuals. This can be better seen in an image below. In addition, almost every point in the Veracruz region, and on much of the coast as a whole, is marked as being from an unscreened individual. This matches the results shown in the later aggregated data analysis. Lastly, the distribution of screened individuals seems to closely follow the location of the Autopista Arraijan-Chorrera, the red line displayed on the map while the unscreened distribution is more scattered and spread out. While the exact reason can currently only be guessed, it may be due to more urban centers being located near the highway and providing more accessible testing services.

Figure 9: Point Map for Toxoplasmosis Screening in the Province of Panama Oeste for Screened (Green) and Unscreened (Purple)

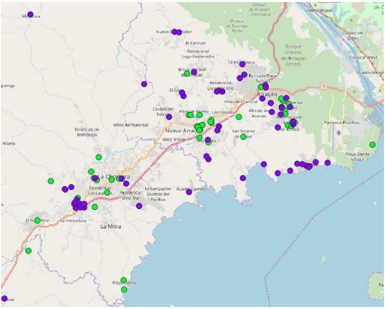

Figure 10: Point Map of Screened (green) and Unscreened (Purple) individuals by Nuevo Arraijan where Screened Individuals are in the Center and Unscreened Individuals on the Periphery of the Region

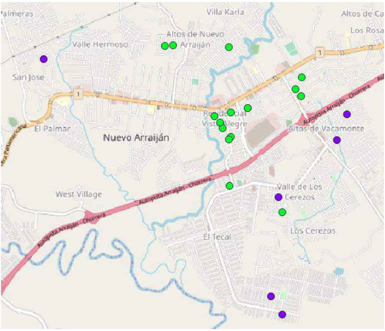

Screening information was also gathered on a national level, so this information was then used to make the following maps.

Figure 11 (a-c): Aggregated Toxoplasmosis Screening Rates Based on Panama's Corregimientos

The first figure shows a natural break heatmap with 3 categories: low, medium, and high. Most of the corregimientos are categorized as "high". A bit of clustering can be seen from this as well, with the lower and medium values being concentrated more in the center. Interestingly one corregimiento, Veracruz, is marked as being in the low category despite all regions surrounding it being high.	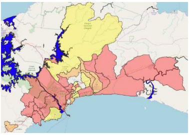
The second figure shows the standard deviation values of each corregimiento's screening rate, and can help identify any particularly high or low points. From this map it appears that Vista Alegre has a particularly high screening rate (2 standard deviations or higher).	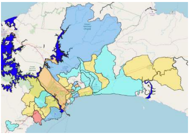
A Moran's I analysis of the data supports much of what can be seen from the natural break and standard deviation heatmap. There is a cluster of high points that can be seen from the western side of the map. The high-low outliers on both furthest sides of the map (Arraijan and Chepo from left to right) were initially slightly surprising based only on	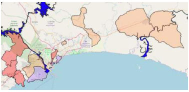

the natural break map. The standard deviation map, however, suggests that in the case of Arraijan, the corregimientos directly to the north and south influence it's result as an outlier. It is important to remember that in the case of Chepo, the high-low outlier furthest to the right, only has one neighbor under a first order queen weight, and should then be regarded with caution as a significant result.<br><br>As past studies have suggested that Curundu is a location of particularly high toxoplasmosis rates, its position on this map as the center of a high value cluster for screening rates makes it another notable result.	

From this analysis of the screening data, a myriad of questions follow. One of the more potentially interesting one is why Vista Alegre's screening rate (72%) is higher than the other corregimientos surveyed, as the following highest values are 67% and 60%. Exploring why this is the case may be helpful in increasing access to screening. Another aspect to investigate with this goal in mind is why Veracruz's screening rate is low (8.3%), especially when compared to the regions. around it. Given what was found in the earlier KDE of unscreened individuals in Panama Oeste, it is possible that is a reflection of lower screening rates in coastal communities.

One of the known risk factors for toxoplasmosis is access to clean, potable water. With this in mind, another analysis done was one mapping seroprevalence values measured through various recent years and investigating potential relationships to water sources.

Figure 12: Aggregated Toxoplasmosis Seroprevalence Rates Based on Panama's Corregimientos Overlaid on Panama's Water Systems

In order to complete this analysis, digitalized Tommy Guardia geographical data was taken from the Smithsonian Tropical Research Institute's website. Here, information regarding lakes, rivers, water basins, and Panama's hydraulic system was taken.	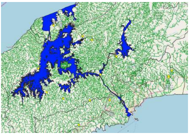
This is a natural break heat map showing IgG seroprevalence taken from INDICASAT's study. This map appears to have a fair bit of clustering, as can be seen by the two large red sections both immediately to the right of the Panama canal and then at the far right of the map.	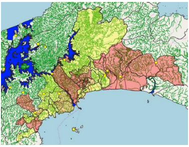
This standard deviation map shows much of the same information as the natural break heatmap. There is a section moving to the east that appears to have a higher seroprevalence than the west, which appears largely blue or yellow. This supports studies done by University of Chicago students in the past, which have suggested that the province of Panama Oeste, to the west of the canal, has a lower seroprevalence rate than Panama.	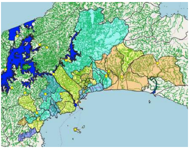

A Moran's I cluster analysis of this data shows both clusters and spatial outliers of both types. As both the natural break and standard deviation maps suggested, this analysis picks up on a high cluster by the eastern corregimientos of Chepo and San Martin. On the western side of the canal, Nuevo Emperador and Vista Alegre are the centers of low seroprevalence clusters. In regards to the two spatial outliers further to the left, their limited number of neighbors casts doubt on any interpretation drawn from their result.	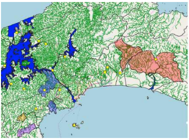

In regards to water, Chepo is divided by the Rio Bayano as well as the Rio Mamoni, the latter of which provides water to IDAAN's Chepo water plants. It may be interesting for future studies to investigate activity around this water, such as the frequency with which people bathe in it or drink from it.

Comparing the results from this analyze to those from the screening data shows noteworthy relationships. Both Vista Alegre and Nuevo Emperador were centers of high clusters for screening and low clusters for seroprevalence. Chepo, although the corregimiento in itself had a relatively high screening rate, was close to locations with low screening rates, and is in this analysis marked as possibly being the center of a cluster, all with high seroprevalence rates. That being said, as it was mentioned in the interpretation of the screening analysis, the results of the Moran's I analysis for Chepo's screening rate is dubious, although other regions in the section appear to have high rates as well.

Figure 13: Kernel Density Maps for Toxoplasmosis Seroprevalence in the Province of Panama

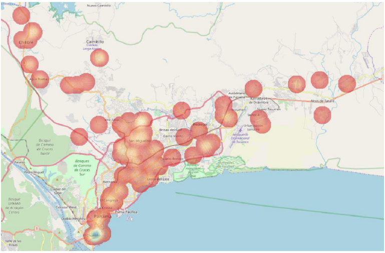

KDE of Negative IgG Points in the Province of Panama (n=97)

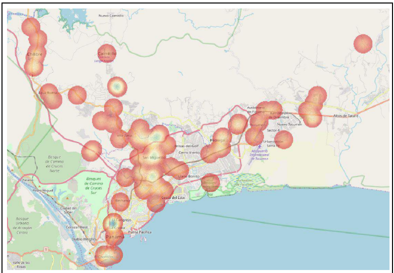

KDE of Positive IgG Points in the Province of Panama (n=83)

In these maps, it appears that the latter map, showing the distribution of IgG positive individuals in the province of Panama, there are fewer individuals west of Llano Bonito. This suggests that there may be a lower seroprevalence rate in this area, although due to the qualitative nature of this method, nothing can be said with certainty.

Figure 14: Kernel Density Maps for Toxoplasmosis Seroprevalence in the Province of Panama Oeste

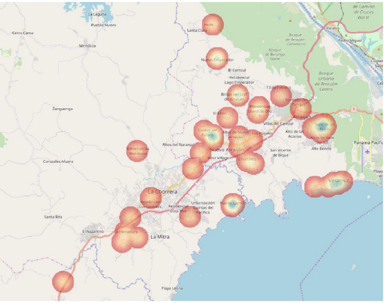

KDE of Negative IgG Points in the Province of Panama Oeste (n=42)

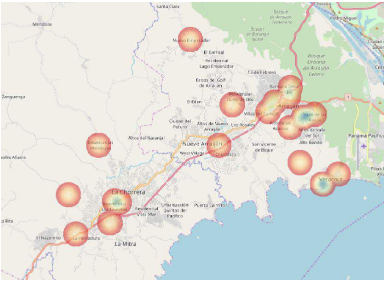

KDE of Positive IgG Points in the Province of Panama Oeste (n=21)

A comparison of these maps appear to show no major differences in the spatial distributions. Although the map of negative points has slightly more points in some locations, that can easily be attributed to the larger sample size in that category. One possible observation is that the points representing individuals positive for IgG are more clustered in the northern and southern areas around the Highway Arraijan, although that may also be attributed to the uneven sample size, making it a fairly weak claim.

LIMITATIONS:

One major limitation in much of these studies was that the information collected often came from one or two sources. For example, the data about congenital and lymphadenopathic

toxoplasmosis came specifically from the University of Quindio's medical records. Variables affecting why the patient chooses to go to this clinic in particular may also impact the data.

Another limitation is that for the risk factor analysis study in Armenia, a major drawing point for the participants was the free ocular exams and blood test. This means that it is possible that people who were already showing symptoms of sickness or who were experiencing ocular issues were more likely to participate in the study, thus falsely inflating the data.

For the Kernel Density Estimator and Moran's I analyses, it is important to remember that the parameters inputted for each analysis may impact the outcome. For example, each KDE map was created with a quartic function, while another sort of influential function may show slightly different locations. In addition, the Moran's I analyses all used a first order queen weight and not a K-Neighbors or distance based weight.

CONCLUSIONS AND FURTHER STUDIES:

Due to the nature of some of the methods used in this paper, it is difficult to make any strong claims regarding the spatial distributions of the variables investigated. That being said, this analysis provides insight as to potential future studies or focuses for targeted policies.

Exploring the relationship between the Highway Arraijan and Toxoplasmosis screening may provide interesting insight as to medical access in Panama Oeste. In the same region, further studies into the coastal communities and where, if anywhere, pregnant women are being screened, could help show gaps in screening accessibility. Another potential future study is one investigating water behaviors and water treatment near the Rio Bayano and the Chepo region of Panama.

The unusual negative correlation between eating undercooked meat and seropositivity in Armenia, Colombia is definitely something which merits further study. This relationship may be due to economic differences, which were not accounted for in the study.Perhaps more affluent persons with better hygiene more easily afford higher grades of meat. Armenia also showed an uneven spatial distribution of prenatal treatment in cases of congenital toxoplasmosis. Doing a more general study to see if this is due to a genuine difference in access to treatment or if it is more due to external factors controlling who goes to the university's clinic may provide useful information.

In all, the use of spatial analysis can be a potentially powerful tool in investigating the spatial nature of things like risk factors and screening rates, both of which frequently tend to have a geographical component due to environmental and physical factors.

A Spatial and Statistical Review and Comparison of Toxoplasmosis Screening and Seroprevalence in Colombia and Panama

Cat Raggi
Universiy of Chicago
catraggi@uchicago.edu

2019

Overview

- Armenia, Quindio, Colombia
  - Congenital
  - Lymphadenopathic
  - Risk factors
- Panama City
  - Seroprevalence
  - Screening rates

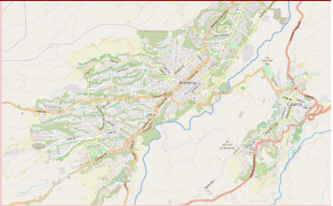

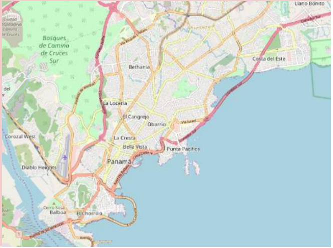

Methods - Colombia

- University of Quindio
- Medical records
- Free eye exams
- Survey

Methods - Panama

- INDICASAT
- Hospital Santo Tomas
  - Past and present
- Hospital San Juan
- Tommy Guardia
- Surveys with free toxo exams

Methods - Analysis

- Logistic regression
- Kernel density maps
- Polygon heat maps
  - Over 5 individuals
  - Natural breaks
  - Standard Deviation
  - Moran’s I Analysis

Colombia

Results - Risk and Protective Factors in Armenia

- Age
- Total population
  - None found
- La Universal
  - None found
- Guaduales de La Villa
  - Drinking bottled water
  - Eating undercooked meat

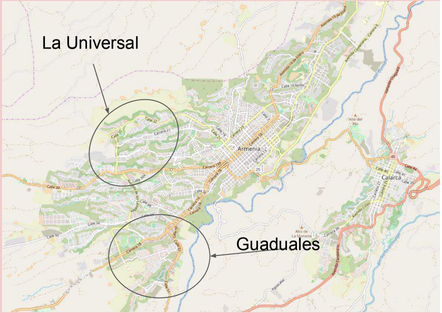

Results - Risk and Protective Factors in Armenia

- No factors found for lesions
- No difference in communities
- Link between bottled water and age

Prenatal Treatment in Congenital Cases in Armenia

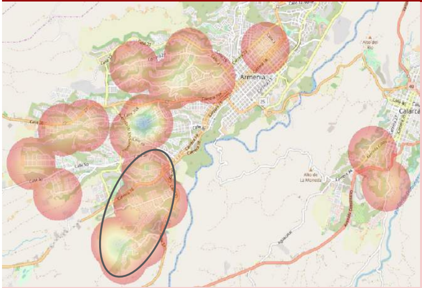

Cases of congenital toxoplasmosis with prenatal treatment found (n=21)

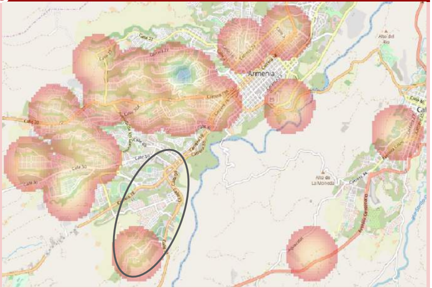

Cases of congenital toxoplasmosis without prenatal treatment found (n=23)

Prenatal treatment higher in Guaduales de la Villa (7/8)

Lymph Node Impairments by Sex in Armenia

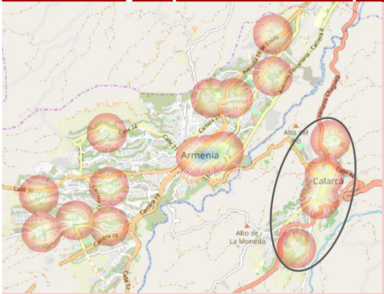

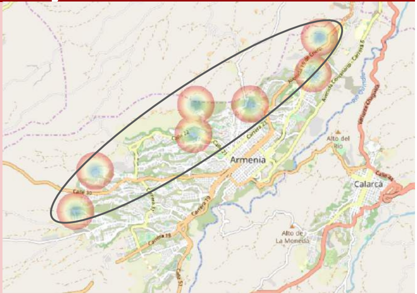

Lymph Node Impairments Related to Toxoplasmosis in Men (n=17)

Lymph Node Impairments Related to Toxoplasmosis in Females (n=7)

Women mostly in North, all cases in Calarca were male

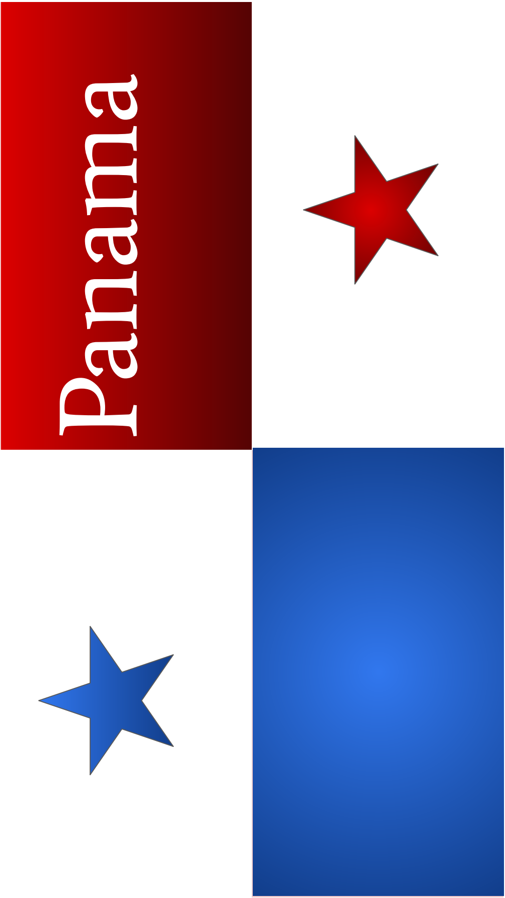
Panama

Toxoplasmosis Screening in Panama

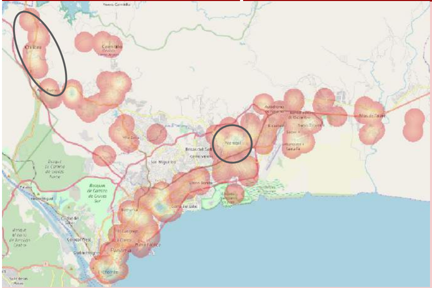

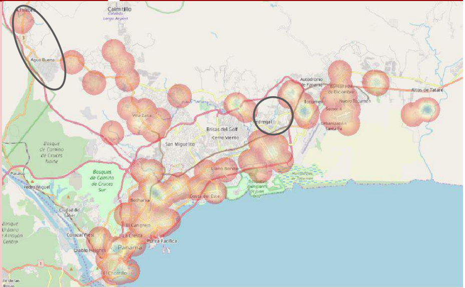

Previously Unscreened Individuals (n=190)

Previously Screened Individuals (n=88)

More unscreened up north, all screened in Pedregal

Toxoplasmosis Screening in Panama Oeste

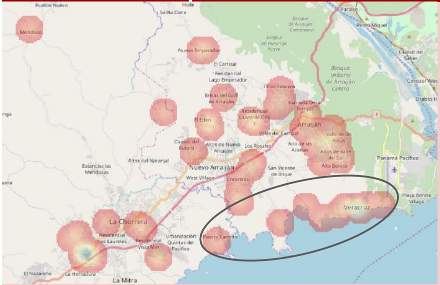

Previously Unscreened Individuals (n=70)

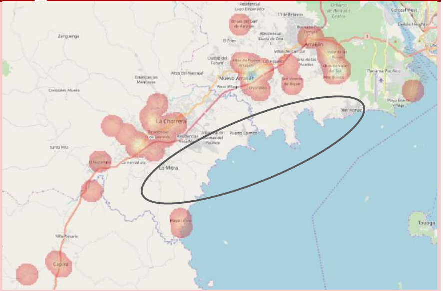

Previously Screened Individuals (n=60)

Screening closer to highway, unscreened coasts

Toxoplasmosis Screening in Panama Oeste

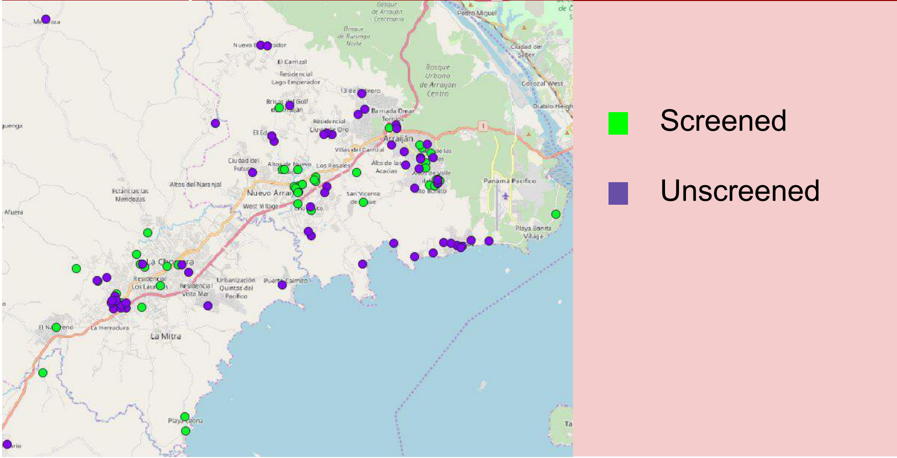

Toxoplasmosis Screening in Panama Corregimientos

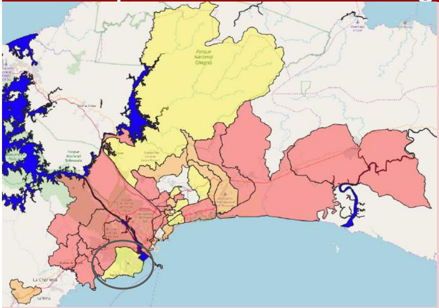

Natural Break Heatmap of Screening Rates

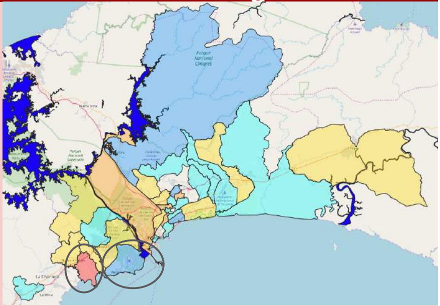

Standard Deviation Heatmap of Screening Rates

Veracruz as an outlier, high screening in Vista Allegre

Toxoplasmosis Screening in Panama Corregimientos

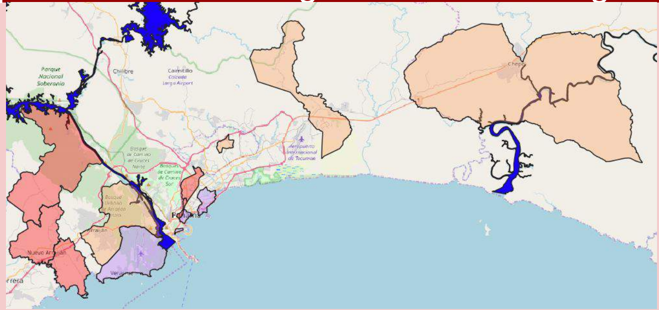

Significant Clusters in a Moran's I Analysis of Screening Rates in Panamanian Corregimientos with a 1st Order Queen Weight

Western cluster, potential outliers

Toxoplasmosis Seroprevalence

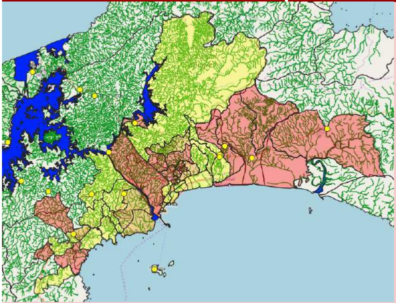

Natural Break Heatmap of Seroprevalence Rates with Hydrologic systems

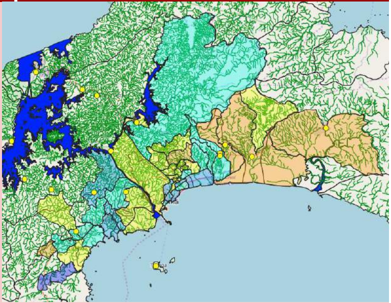

Standard Deviation Heatmap of Seroprevalence Rates with Hydrologic systems

Higher in Easternmost corregimientos and east of the canal

Toxoplasmosis Seroprevalence

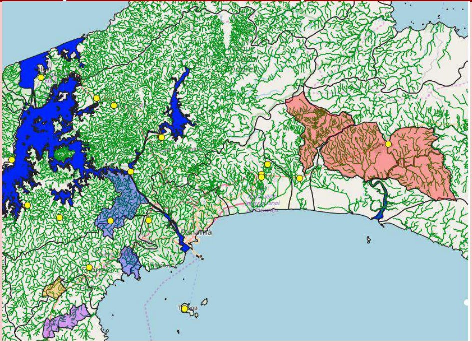

Significant Clusters in a Moran’s I Analysis of Seroprevalence Rates in Panamanian Corregimientos with a 1st Order Queen Weight

Low clusters in west, high clusters in east

Conclusion

- Uncooked meat / Bottled water
- Prenatal treatment in Guaduales de la Villa
- Highway and coastal screening
- Veracruz / Vista Allegre screening
- Seroprevalence higher in eastern Panama

Further Work

- Uncooked meat as a protective factor
- Prenatal treatment in Armenia
- Panama Oeste’s coastal communities
- Pedregal

Acknowledgements

Thank you to everyone at GEPAMOL at the University of Quindio and at INDICASAT as well as the University of Chicago students for their past work!

Credits

Jose Sanchez, Jorge Enrique Gómez Marín, Zuleima del Carmen Caballero, Alejandra de-la-Torre, Juliana Muñoz, Alejandro Acosta Dávila, Laura Lorena López, Elizabeth Torres, Juan David Valencia, Manuela Mejia Oquendo,Daniel Celis, Mónica Vargas, Delba Villalobos, María Cristina Bohórquez, José Yashin Artega Rivera, Nocolás Rivera Valdivia, Mariangela Soberon Felin, Abhi Pandya, Aliya Moreira, Kanix Wang, Stefany Velasco, Daniel Celis Giraldo, Catherine Castro, Margarita Ramirez, Davina Moosazadeh, Osvaldo Reyes, Mayrene Ladrón de Guevera, Connie Mendivil, Mario L. Quijada, Anabel Garcia, Guillermo Pradieu, Dora Estripeaut, Ximena Norera, Carlos Flores, Jovanna Borace, Andrey Rzhetsky, Sharon Heichman, Rima McLeod

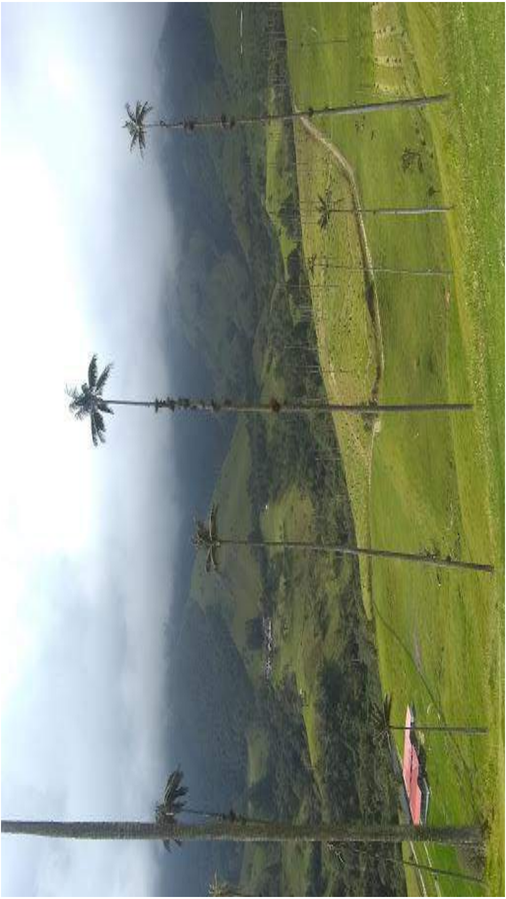

A Spatial and Statistical Review and Comparison of Toxoplasmosis Screening and Seroprevalence in Colombia and Panama

Authors: Cat Raggi, Jose Sanchez, Mariangela Soberon Felin, Jorge Enrique Gómez Marín, Kanix Wang, Mayrene Ladrón de Guevera, Laura Lorena López, Mónica Vargas, Zuleima del Carmen Caballero, Connie Mendivil, Mario L. Quijada, Anabel Garcia, Alejandra de-la-Torre, Juliana Muñoz, John Alejandro Acosta Dávila, Elizabeth Torres, Juan David Valencia, Manuela Mejía, Daniel Celis, Delba Villalobos, María Cristina Bohórquez, José Yashin Artega Rivera, Nocolás Rivera Valdivia, Abhi Pandya, Aliya Moreira, Catherine Castro, Margarita Ramirez, Davina Moosazadeh, Guillermo Pradieu, Dora Estri[eat, imena Norera, Carlos Flores, Jovanna Borace, Andrey Rzhetsky, Jagantha Rao, Xavier Saez-Llorenz, Osvaldo Reyes, Rima McLeod

Goal: The purpose of this study was to identify areas where congenital, ocular, and lymphadenopathic toxoplasmosis were focused in Armenia, Colombia and any risk factors that may be associated. In addition, rates of seroprevalence and gestational screening in Panamanian corregimientos were mapped and observed in relation to their water sources.

Introduction: *Toxoplasma* is a parasite that can cause congenital toxoplasmosis if initial infection occurs during a woman's pregnancy. The infection, while often asymptomatic in the immunologically competent adult, can also manifest complications such as ocular lesions, lymphadenopathy, and severe disease in immunologically compromised persons, and with hypervirulent parasites and human host genetic susceptibility..

Methods: Data for this study was taken from surveys and medical records from the University of Quindio, a survey of pregnant women from Panama's INDICASAT, and past studies done with University of Chicago and INDICASAT at Hospital Santo Tomas in Panama City. Water data was taken from digitized Tommy Guardia sources. The analysis was done using both qualitative and quantitative spatial methods, as well as log odds regression. If point data was available, kernel density estimator heatmaps were created and compared to look at spatial distributions. When possible, data points were also condensed and summarized into regional data for polygon based analyses such as natural break and standard deviation heatmaps, and a Moran's I cluster analysis to look for spatial clustering or outliers.

Results: The risk factor analysis in Armenia found that drinking from bottled water and eating undercooked meat were protective factors in one of the two communities tested. An exploration of the point data suggests various potential spatial differences in prenatal treatment for congenital toxoplasmosis in Armenia, as well as in screening in the Panamanian provinces of Panama and Panama Oeste. The analyses suggest a cluster of high screening rates in the westernmost corregimientos surveyed and a particularly high screening rate in Vista Allegre. Toxoplasmosis seroprevalence also appears to be highest in eastern corregimientos.

Conclusion: These statistical analyses and explorations can be used to identify geographic trends, advise future preventative measures and policies, and locate areas in which more localized studies may provide useful results.
